# Genome-Wide Identification and Salinity Response Analysis of the Germin-like Protein (*GLP*) Gene Family in *Puccinellia tenuiflora*

**DOI:** 10.3390/plants14152259

**Published:** 2025-07-22

**Authors:** Yueyue Li, Zhe Zhao, Bo Li, Hongxia Zheng, Zhen Wu, Ying Li, Meihong Sun, Shaojun Dai

**Affiliations:** 1 Key Laboratory of Saline-Alkali Vegetation Ecology Restoration, College of Life Sciences, Northeast Forestry University, Ministry of Education, Harbin 150040, China; 2021031283@nefu.edu.cn (Y.L.); lb19941016@163.com (B.L.); zhenghongxia2020@foxmail.com (H.Z.); 2 Development Center of Plant Germplasm Resources, College of Life Sciences, Shanghai Normal University, Shanghai 200234, China; lmy2121@126.com (Z.Z.); zhen_wu@shnu.edu.cn (Z.W.)

**Keywords:** *Puccinellia tenuiflora*, germin-like protein (*GLP*) gene family, expression pattern, salt tolerance

## Abstract

The germin-like protein (GLP) family plays vital roles for plant growth, stress adaptation, and defense; however, its evolutionary dynamics and functional diversity in halophytes remain poorly characterized. Here, we present the genome-wide analysis of the GLP family in the halophytic forage alkaligrass (*Puccinellia tenuiflora*), which identified 54 *PutGLPs* with a significant expansion compared to other plant species. Phylogenetic analysis revealed monocot-specific clustering, with 41.5% of *PutGLPs* densely localized to chromosome 7, suggesting tandem duplication as a key driver of family expansion. Collinearity analysis confirmed evolutionary conservation with monocot *GLPs*. Integrated gene structure and motif analysis revealed conserved cupin domains (BoxB and BoxC). Promoter *cis*-acting elements analysis revealed stress-responsive architectures dominated by ABRE, STRE, and G-box motifs. Tissue-/organ-specific expression profiling identified root- and flower-enriched *PutGLPs*, implying specialized roles in stress adaptation. Dynamic expression patterns under salt-dominated stresses revealed distinct regulatory pathways governing ionic and alkaline stress responses. Functional characterization of PutGLP37 demonstrated its cell wall localization, dual superoxide dismutase (SOD) and oxalate oxidase (OXO) enzymatic activities, and salt stress tolerance in *Escherichia coli*, yeast (*Saccharomyces cerevisiae INVSc*1), and transgenic *Arabidopsis*. This study provides critical insights into the evolutionary innovation and stress adaptive roles of GLPs in halophytes.

## 1. Introduction

Germin-like proteins (GLPs) were first identified during wheat seed germination and have since been characterized across diverse plant lineages including gymnosperms, dicots, monocots, and mosses [1,2,3,4]. As members of the versatile cupin superfamily, all GLPs possess conserved cupin domains characterized by a β-barrel structure that facilitates metal ion binding [5]. These proteins exhibit exceptional stability as oligomeric complexes, demonstrating remarkable resistance to thermal denaturation and proteolytic degradation [6]. In plants, GLPs contribute to multiple biological processes such as developmental regulation, osmoregulation, and pathogen defense through cell wall fortification mechanisms [5,7,8,9].

The majority of GLPs exhibit enzymatic activities, including oxalate oxidase (OXO), superoxide dismutase (SOD), and cysteine peptidase functions [10,11,12]. For example, rice (*Oryza sativa*) OsGLP3-7 demonstrates SOD activity and regulates H_2_O_2_ accumulation in transgenic plants [12]. Enzymatic assays of *Calotropis procera* latex fluids identified OXO activity in the latex-derived CpGLP1 and CpGLP2 proteins [13]. Similarly, the azalea *(Rhododendron mucronatum*) GLP (RmGLP2) exhibited OXO activity in both in vitro and in vivo assays, while negative staining further revealed its concurrent SOD activity [14]. Notably, a GLP purified from *Thevetia peruviana* demonstrated proteolytic activity characteristic of cysteine peptidase [15].

GLPs participate in plant developmental processes and mediate responses to biotic and abiotic stresses, potentially through enhanced enzymatic activity [4,16]. In rice, suppression of *OsGLP2-1* expression accelerated dormancy release in immature seeds, while its overexpression enhanced seed dormancy [8]. Notably, the rice GLP (OsGER4) acts as a negative regulator of gibberellin (GA)-mediated crown root development [17]. Furthermore, in *Arabidopsis*, plasmodesmata GLP (PDGLP1/2) regulate root architecture by controlling phloem-mediated resource allocation between primary and lateral root meristems [18]. Critically, GLPs also mediate biotic and abiotic stress responses. The *Craterostigma plantagineum* CpGLP1, which defines a novel GLP subfamily, demonstrates SOD activity and pectin-binding capacity, coordinating ROS homeostasis and cell wall remodeling during desiccation tolerance [19]. Likewise, transgenic expression of the sunflower *(Helianthus annuus*) HaGLP1 in *Arabidopsis* conferred significant tolerance to *Sclerotinia sclerotiorum* and protects against *Rhizoctonia solani* infection, likely through elevated ROS levels [20]. Recently, OsGLP8-7 in rice was shown to alleviate copper toxicity through lignin biosynthesis-mediated cell wall remodeling, which enhances Cu^2+^ retention, suppresses oxidative stress, and preserves cellular ultrastructure under excess Cu conditions [21].

Alkaligrass (*Puccinellia tenuiflora*), a halophytic forage species exhibiting exceptional saline-alkaline stress tolerance [22], serves as a model organism for studying stress resistance mechanisms with applications in saline soil remediation and functional genomics [23,24]. Although GLP gene family has been functionally characterized in soybean (*Glycine max*), longan (*Dimocarpus longan*), Citrus (*Citrus sinensis*), peanut (*Arachis hypogaea*), melon (*Cucumis melo*), rapeseed (*Brassica napus*), cotton (*Gossypium hirsutum*), maize (*Zea mays*), rice (*O*. *sativa*), barley (*Hordeum vulgare*), *Arabidopsis* (*Arabidopsis thaliana*), and moss (*Physcomitrella patens*) [3,4,16,25,26,27,28,29,30,31,32], research on the GLP family in *P. tenuiflora* remains limited.

In this study, we aim to comprehensively identify and annotate *PutGLP* members through genome-wide analysis. We will elucidate their evolutionary relationships, structural features, expression levels across tissues and organs, and salinity-responsive dynamics. On these bases, we further functionally validate the key *PutGLP37* via subcellular localization, enzymatic assays, and phenotypic analysis in transgenic plants. These objectives will establish a foundation for deciphering the molecular mechanisms by which *PutGLPs* mediate development and environmental resilience in halophytes.

## 2. Results

### 2.1. Identification of PutGLPs in P. tenuiflora

Using a Hidden Markov Model (HMM) scan and BLASTP searches, 53 *PutGLPs* were identified in the *P. tenuiflora* genome, designated as *PutGLP1*-*PutGLP36* and *PutGLP38*-*PutGLP54* (Table S1). PutGLP37 was subsequently isolated from the *P. tenuiflora* cDNA library but lacked a corresponding genomic position in the published genome assembly [22]. Therefore, a total of 54 family members encoding the cupin_1 domain (PF00190) were analyzed. The amino acid sequences varied in length from 171 residues (PutGLP33) to 280 residues (PutGLP4), with predicted theoretical isoelectric points (pI) ranging from 4.51 (PutGLP46) to 9.51 (PutGLP11) (Table S1). The predicted molecular weights (MW) of these proteins ranged from 18.33 kDa (PutGLP33) to 30.91 kDa (PutGLP4). Subcellular localization predictions indicated that most PutGLP proteins localize to the cell wall.

### 2.2. Phylogenetic and Evolutionary Analysis of the PutGLPs

**Figure 1:**
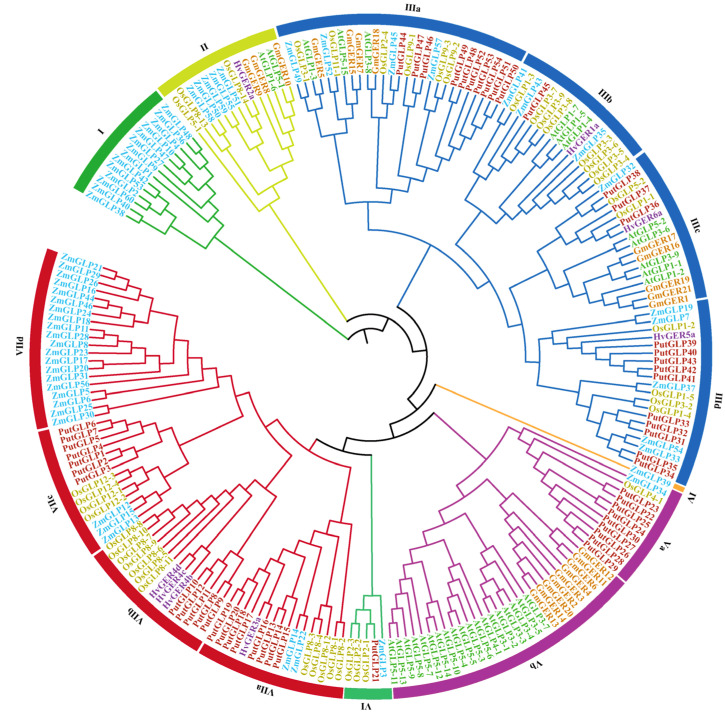
Phylogenetic tree constructed from GLP protein sequences of alkaligrass (*Puccinellia tenuiflora*), soybean (*Glycine max*), barley (*Hordeum vulgare*), *Arabidopsis* (*Arabidopsis thaliana*), rice (*Oryza sativa*), and maize (*Zea mays*). Different-colored circles represent distinct phylogenetic groups, whereas gene names are color-coded according to their respective species.

To investigate the functional and evolutionary relationships within the GLP gene family, an unrooted phylogenetic tree was constructed using sequences from six plant species: alkaligrass (*P. tenuiflora*), soybean *(G. max*), barley (*H. vulgare*), *Arabidopsis* (*A. thaliana*), rice (*O. sativa*), and maize (*Z. mays*) (Figure 1 and Table S2). The 218 *GLPs* analyzed were classified into seven major clades (I–VII). *PutGLPs* were distributed across clades III, V, VI, and VII, comprising 24, nine, one, and 20 members, respectively. Among all clades, clade III represented the largest group, encompassing 80 *GLP* members (36.7% of the total). This clade was further subdivided into four subgroups: IIIa (ten *PutGLPs*), IIIb (one *PutGLP*), IIIc (three *PutGLPs*), and IIId (ten *PutGLPs*). Similarly, clade VII was partitioned into subclades VIIa (eight *PutGLPs*), VIIb (five *PutGLPs*), VIIc (seven *PutGLPs*), and VIId (no *P. tenuiflora* representatives). Maize ZmGLPs in subclade VIId only contain cupin_1 domain and cupin_2 domain, while PutGLPs within subclade VIIc have cupin_1 domain, cupin_2 domain, and cupin_3 domain, suggesting potential functional divergence.

### 2.3. Chromosomal Distribution and Collinearity Analysis of the PutGLPs

**Figure 2:**
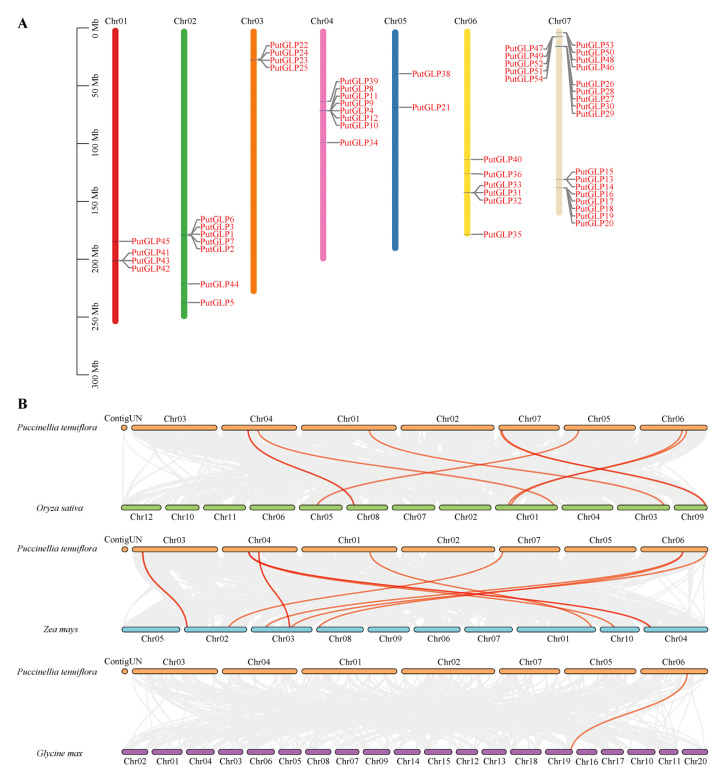
Chromosomal distribution and collinearity analysis of *PutGLP*s in *Puccinellia tenuiflora*. (**A**) Chromosomal localization of *PutGLPs* across the *P. tenuiflora* genome. (**B**) Comparative collinearity analysis of *GLPs* between alkaligrass (*P. tenuiflora*) and three angiosperm species, rice (*Oryza sativa*), maize (*Zea mays*), and soybean (*Glycine max*). Gray lines depict collinear genomic regions between *P. tenuiflora* and the reference species, while red lines specifically denote syntenic *GLP* gene pairs.

Chromosomal distribution analysis revealed that 53 *PutGLP*s were unevenly distributed across all seven chromosomes of *P. tenuiflora* (Figure 2A). Chromosomes 1 and 3 each harbored four *PutGLPs*, while chromosomes 2, 4, and 6 contained seven, eight, and six *PutGLPs*, respectively, while chromosome 5 carried only two *PutGLPs*. Notably, chromosome 7 exhibited the highest density with 22 *PutGLP*s, accounting for 41.5% of the total.

To elucidate evolutionary relationships, we performed collinearity analysis between *P. tenuiflora* and three related species (rice, maize, and soybean) (Figure 2B). The results identified nine orthologous *GLP* pairs between *P. tenuiflora* and rice, 11 pairs with maize, and one pair with soybean. Notably, *PutGLP54* showed homologous relationships with both *OsGLP9-1* and *ZmGLP57*, while *PutGLP36* exhibited homology with *OsGLP1-1* and *GmGER1*. The more homologous gene pairs from *PutGLPs* with rice and maize than with soybean indicate closer phylogenetic affinity of *PutGLPs* to monocotyledons than to dicotyledons.

### 2.4. Gene Structure, Conserved Domain, and Motif Analysis of PutGLPs

**Figure 3:**
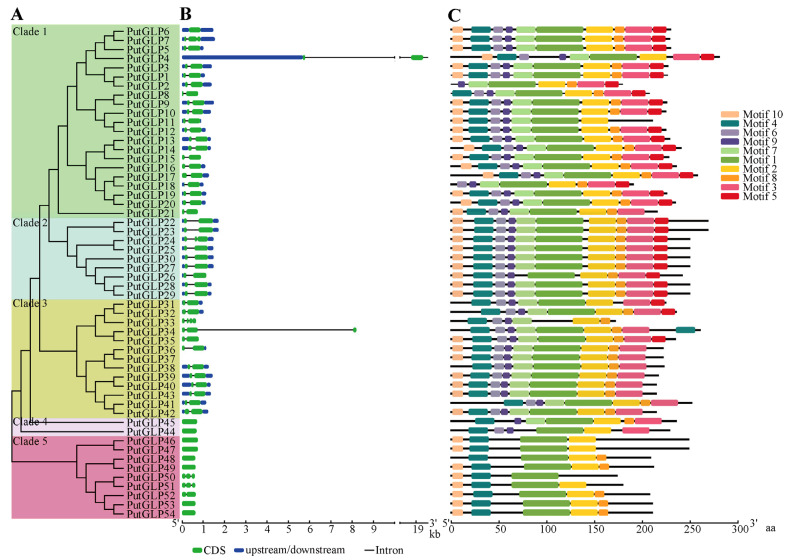
Phylogenetic, structural, and motif analysis of PutGLPs. (**A**) Neighbor-Joining phylogenetic tree of PutGLP proteins. Bootstrap values (1000 replicates) are labeled at branch nodes. (**B**) Genomic organization of *PutGLPs*. Structural components are annotated as follows: untranslated regions (UTRs, blue boxes), exons (green boxes), and introns (black lines). Scale bar indicates sequence length. (**C**) Distribution of conserved protein motifs. Ten motifs (Motif 1–10) are identified and color-coded with numerical identifiers. Motif positions are mapped to the corresponding protein sequences.

To elucidate the structural and functional characteristics of the GLP gene family in *P. tenuiflora*, the gene structure, conserved domain, and conserved motif of *PutGLPs* were analyzed (Figure 3). A phylogenetic tree constructed from the *PutGLPs* divided them into five distinct clades (Figure 3A). Gene structure analysis revealed that *PutGLPs* contain one to four exons (Figure 3B). Motif prediction analysis identified three conserved domains: motif 1, motif 2, and motif 4, which encode the BoxB, BoxA, and BoxC domains, respectively (Figure 3C and Figure S1). These motifs (motif 1, motif 2, and motif 4) were observed in all PutGLPs except PutGLP18 and PutGLP33. Multiple sequence alignment further confirmed that the GLP proteins contain BoxA, BoxB, and BoxC domains, with the cupin domain comprising BoxB and BoxC (Figures S2 and S3). Additionally, two conserved cysteine residues were identified in the aligned sequences (Figure S2).

### 2.5. Analysis of Cis-Acting Elements of PutGLP Promoters

**Figure 4:**
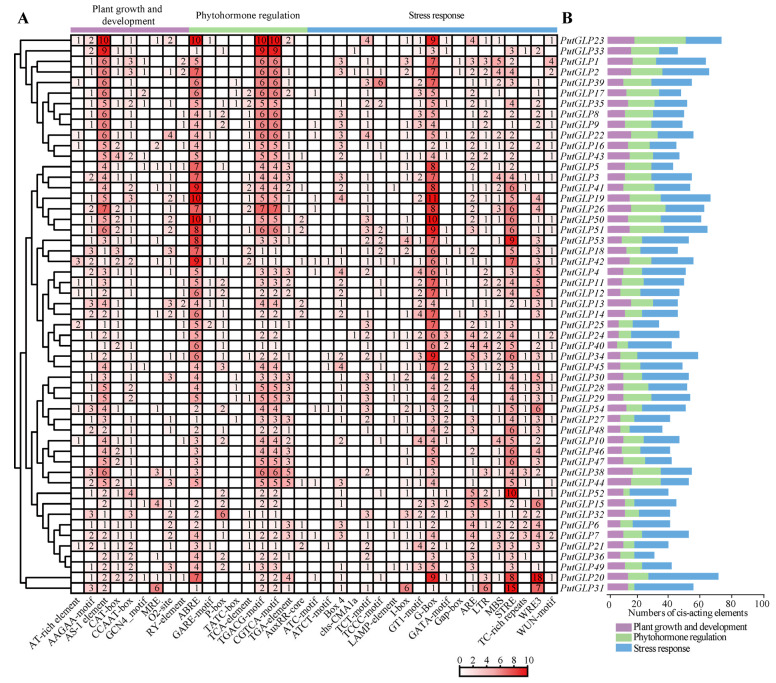
Quantitative and functional profiling of *cis*-acting elements in *PutGLP*s. (**A**) Heatmap visualization of *cis*-acting element abundance across *PutGLPs*. Element counts are color-coded (red intensity gradient) and numerically annotated in grid cells. (**B**) Functional classification of *cis*-acting elements. Grouped bar plots quantify elements associated with three biological processes, plant growth and development (purple), phytohormone regulation (green), and stress response (blue).

To better understand potential regulation and function of *PutGLPs*, we analyzed *cis*-acting elements within the 3000-bp upstream promoter regions of *PutGLP* members (Figure 4A,B). A total of 2619 *cis*-acting elements were identified and categorized into three functional groups based on previous research [33]: plant growth and development, phytohormone regulation, and stress response. Stress-responsive elements constituted nearly half of all elements (1282/2619, 48.9%), with the G-box (10.84%) and STRE (8.29%) being the two most prevalent. Among phytohormone regulatory elements, abscisic acid (ABA)-responsive element (ABRE), TGACG-motif, and CGTCA-motif collectively accounted for the highest proportion (25.47%). Notably, ABRE was detected in all *PutGLP*s except *PutGLP52*. Within the plant growth and development category, nine elements were identified, of which AS-1 exhibited the highest proportion (7.75%).

### 2.6. Subcellular Localization of PutGLP37 and Expression Profiles of PutGLPs in Various Organs

**Figure 5:**
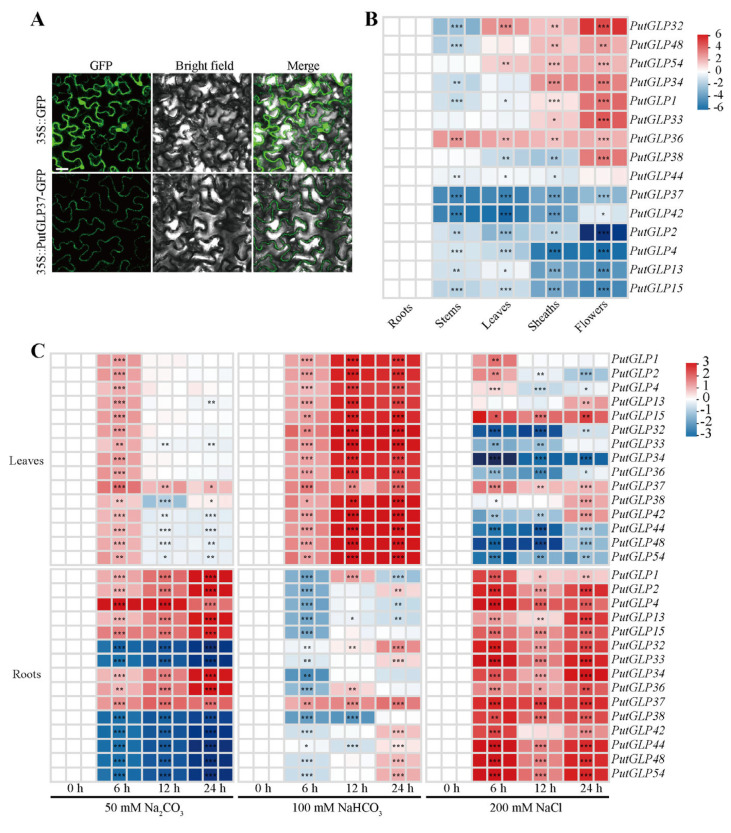
Subcellular localization and expression dynamics of *PutGLPs*. (**A**) Transient subcellular localization of PutGLP37 in *Nicotiana benthamiana* epidermal cells. Confocal micrographs show GFP fluorescence (green channel) from leaves co-infiltrated with *Agrobacterium tumefaciens* strains carrying either *35S::GFP* (vector control) or *35S::PutGLP37-GFP* constructs. Images were captured at 48 h post-infiltration. Scale bar = 25 μm. (**B**) Tissue-/Organ-specific expression profiles of 15 *PutGLPs*. Real-time quantitative PCR (RT-qPCR) analysis quantifies transcript abundance in five organs: roots, stems, leaves, sheaths, and flowers. Expression values were normalized to *PutActin* using the 2^−ΔΔCt^ method. (**C**) Salt stress-responsive regulation of *PutGLPs* in leaves and roots. Heatmaps display log_2_-transformed fold-changes in expression levels under three sodium treatments: 50 mM Na_2_CO_3_, 200 mM NaCl, and 100 mM NaHCO_3_, sampled at 0, 6, 12, and 24 h post-treatment. Color gradients (red: upregulation; blue: downregulation) reflect standardized expression relative to untreated controls (0 h). Significant differences compared to the control group were determined by Student’s *t*-test, *** *p* < 0.001, ** *p* < 0.01, and * *p* < 0.05.

To determine the subcellular localization of PutGLP37, we performed transient expression assays in tobacco (*Nicotiana benthamiana*) leaves (Figure 5A). The coding sequence of *PutGLP37* was fused in-frame to the C-terminus of GFP under the control of the CaMV 35S promoter. Both the *35S::PutGLP37-GFP* fusion construct and *35S::GFP* control were transiently expressed in tobacco epidermal cells, respectively. Results revealed that the GFP-only control localized diffusely in the cytosol, nucleus, and plasma membrane (PM). In contrast, PutGLP37-GFP was predominantly localized to the cell wall and PM (Figure 5A and Figure S4).

To investigate potential functional roles, we analyzed the expression patterns of *PutGLPs* across five tissues (roots, stems, leaves, sheaths, and flowers) using RT-qPCR (Figure 5B). Based on relative expression levels, *PutGLPs* were classified into three distinct groups. *PutGLP2*, *PutGLP4*, *PutGLP13*, *PutGLP15*, *PutGLP37*, and *PutGLP42* exhibited the highest expression in roots. *PutGLP1*, *PutGLP32*, *PutGLP33*, *PutGLP34*, *PutGLP38*, *PutGLP48*, and *PutGLP54* were predominantly expressed in flowers, while *PutGLP36* was exclusively expressed in stems.

### 2.7. Expression Patterns of PutGLPs in Roots and Leaves Under Various Abiotic Stresses

To validate the potential involvement of *PutGLPs* in stress responses suggested by *cis*-acting element analysis, we investigated the expression level of 15 *PutGLPs* in roots and leaves under various salt treatments including Na_2_CO_3_, NaCl, and NaHCO_3_ (Figure 5C). In roots, Na_2_CO_3_ treatment led to upregulation in half of the 15 tested genes (8/15) and downregulation in the remaining half (7/15). Strikingly, all 15 genes exhibited varying degrees of upregulation under NaCl treatment. Under NaHCO_3_ stress, 14 *PutGLPs* showed suppressed expression at 6 h, except *PutGLP37*. Notably, *PutGLP1* and *PutGLP36* reached peak expression levels at 12 h, while *PutGLP2*, *PutGLP32*, *PutGLP33*, *PutGLP37*, *PutGLP42*, *PutGLP44*, *PutGLP48*, and *PutGLP54* peaked at 24 h. In leaves, all 15 genes displayed maximal expression at 6 h under Na_2_CO_3_ treatment. Remarkably, *PutGLP15* and *PutGLP37* showed sustained upregulation throughout NaCl treatment, whereas all 15 genes were significantly upregulated at 12 h and 24 h under NaHCO_3_ stress compared to the control.

### 2.8. Tolerance Analysis by Overexpressing PutGLP37 in Yeast and E. coli

To investigate the biological role of PutGLP37, we expressed this gene in yeast *(S. cerevisiae INVSc*1) and *E. coli*. Under non-stress conditions in YPD medium, the growth of *pYES2-PutGLP37* yeast strains was comparable to the *pYES2* empty vector control (Figure S5A). However, when exposed to osmotic stressors including 1.3 M NaCl, 26 mM NaHCO_3_, or 12 mM Na_2_CO_3_, the *pYES2-PutGLP37* strains exhibited significantly enhanced growth relative to the control, indicating that PutGLP37 conferred tolerance to ionic and alkaline stresses. To further validate this stress-adaptive function, we overexpressed PutGLP37 in *E. coli* as well (Figure S5B). On Luria-Bertani (LB) agar plates supplemented with 100 mM or 250 mM NaCl, *E. coli* cells expressing *PutGLP37* showed markedly improved growth compared to the control strain. This phenotype was corroborated in liquid culture assays in liquid LB medium containing 250 mM NaCl; the *PutGLP37*-expressing strain displayed a significantly higher growth rate than the control (Figure S5C). These results demonstrate that PutGLP37 enhances NaCl stress tolerance in prokaryotic systems.

### 2.9. SOD and OXO Activity Assays of Recombinant PutGLP37 Protein

**Figure 6:**
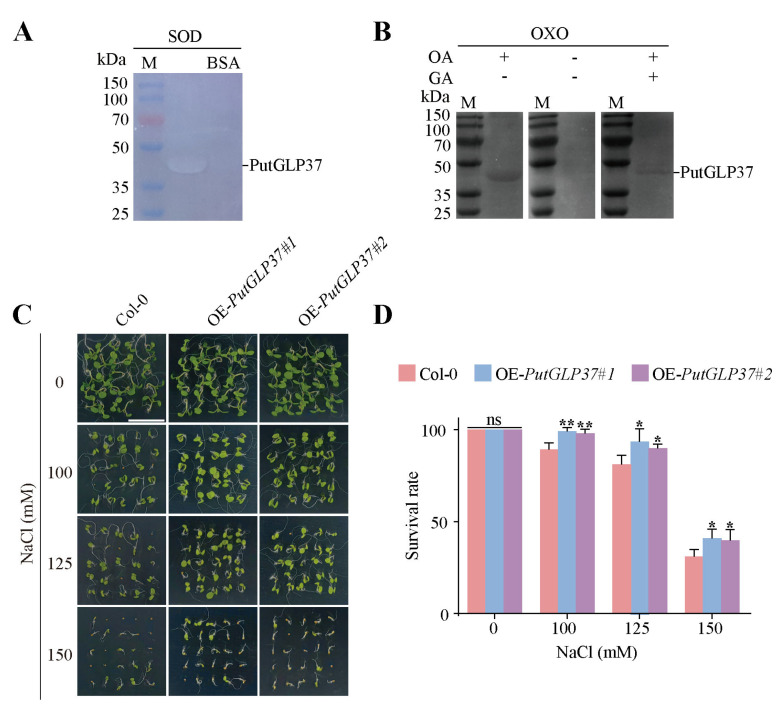
Functional characterization of PutGLP37 in enzymatic activities and salt stress phenotypes in transgenic *Arabidopsis*. (**A**) Superoxide dismutase (SOD) activity analysis. The semi-native PAGE showing SOD activity bands in PutGLP37 samples. Bovine serum albumin (BSA) served as negative control. (**B**) Oxalate oxidase (OXO) activity assay of PutGLP37 through in-gel activity staining. Reaction mixtures containing 2 mM oxalic acid (OA) or glycolic acid (GA) were incubated with purified protein. (**C**) Salt stress tolerance phenotypes of wild-type (Col-0) and *PutGLP37-*overexpression *Arabidopsis* lines. The seedlings grown under 22 °C were treated with 0–150 mM NaCl for 7 days under 16 h light/8 h dark conditions at 22 °C. (**D**) Survival rate quantification post-NaCl treatment. Each bar indicated the mean ± standard deviation (SD) (n = 4, significant differences compared to the control group were determined by Student’s *t*-test, ** *p* < 0.01 and * *p* < 0.05; “ns” represents no significance).

Previous studies have demonstrated that barley GLP proteins exhibit both OXO and SOD activities, which are critical for their defensive roles [34,35,36]. To assess whether PutGLP37 shares these enzymatic functions, we purified the recombinant protein and measured its SOD and OXO activities (Figure 6A,B). A semi-native PAGE gel assay confirmed that recombinant PutGLP37 was properly folded and enzymatically active, indicating potential SOD activity (Figure 6A). OXO activity was further evaluated using in-gel activity staining (Figure 6B). Incubation with oxalate revealed strong OXO activity in purified PutGLP37, whereas supplementation with the inhibitor glycolic acid significantly suppressed this activity, confirming reaction specificity.

### 2.10. Salt-Responsive Phenotype of PutGLP37-Overexpressing Arabidopsis Plants

To investigate the function of PutGLP37 in salt tolerance, we generated *PutGLP37*-overexpressing *Arabidopsis* plants under the control of the strong constitutive CaMV 35S promoter and compared survival rates between Col-0 and *PutGLP37*-overexpressing seedlings under NaCl treatment (Figure 6C,D). Under normal conditions, survival rates did not differ significantly between *PutGLP37*-overexpressing plants and Col-0 controls. In contrast, NaCl stress significantly increased green seedling survival in *PutGLP37*-overexpressing plants compared to Col-0, with the most pronounced difference observed under 150 mM NaCl. These results suggest that PutGLP37 may play a role in salt stress response mechanisms.

## 3. Discussion

### 3.1. Evolutionary Expansion and Stress-Adaptive Innovation of the PutGLP Family in P. tenuiflora

Research on the GLP gene family in plants reveals evolutionary expansion of *GLPs*. Currently, this gene family has been characterized in 12 species, comprising dicotyledons such as soybean (*G*. *max*) (21 *GLPs*), longan (*D. longan*) (35 *GLPs*), Citrus (*C. sinensis*) (57 *GLPs*), peanut (*A. hypogae*a) (84 *GLPs*), melon (*C. melo*) (22 *GLPs*), rapeseed (*B. napus*) (77 *GLPs*), and cotton (*G. hirsutum*) (106 *GLPs*), monocotyledons such as maize (*Z. mays*) (60 *GLPs*), rice (*O. sativa*) (43 *GLPs*), barley (*H. vulgare*) (80 *GLPs*), and *Arabidopsis* (*A. thaliana*) (32 *GLPs*), and bryophytes moss (*P. patens*) (77 *GLPs*) [3,4,16,25,26,27,28,29,30,31,32]. In this study, 54 *PutGLPs* were systematically identified in the halophytic model species *P. tenuiflora* (Figure 1). Compared to species exhibiting *GLP* gene expansion (such as *B. napus*, moss, cotton, and barley), members of PutGLP gene family also show significant expansion, potentially reflecting genomic adaptations to extreme saline-alkaline habitats of *P. tenuiflora*.

Previous evolutionary analyses of rice GLP family showed that 23 rice *GLPs* and 13 *Arabidopsis GLPs* clustered together, whereas maize GLP family analysis revealed that distinct clustering of 32 rice and 41 maize *GLPs* [29,30]. Here, *PutGLPs* primarily clustered in Clade III (24 *GLPs*) and Clade VII (20 *GLPs*), with 47 and 25 monocot GLPs, respectively (Figure 1), indicating monocot-specific orthology. Chromosomal distribution analyses further suggested that tandem duplication drives GLP family expansion across species. For instance, chromosome 2 contained 15 of 35 *GLPs* in *D. longan* [25], while 38 of 84 *GLPs* formed duplicated gene pairs in peanut [26]. Moreover, rice chromosome 8 harbored 14 of 43 *GLPs* in rice [30], *Arabidopsis* chromosome 5 carried 12 of 32 *GLPs* [30], while melon chromosome 8 contained 8 of 22 *GLPs* [27]. Here, we found the high density of *PutGLPs* on chromosome 7 (22 of 53) (Figure 2A). This suggests that gene family expansion of PutGLP gene family was highly likely to be driven by tandem duplication or chromosomal rearrangements.

Conserved cupin domains are essential for GLP function, enabling enzymatic activities such as OXO and SOD, as well as roles in plant development and defense [5]. These domains occur universally across GLPs in diverse species [3,4,16,25,26,27,28,29,30,31,32]. Our study confirms the conserved cupin domain is also ubiquitous within PutGLPs (Figure 3C), demonstrating its extensive conservation across the GLP family. This further underscores the conserved functional importance of cupin domains in fundamental enzymatic activities like OXO and SOD [37].

Promoter *cis*-acting elements critically govern gene expression patterns, stress responses, and biological functions, such as hormone-responsive elements (AREB) and stress-responsive elements (STRE) [38,39,40]. ABRE-binding transcription factors were reported to mediate ABA-triggered chlorophyll degradation and leaf senescence in *Arabidopsis* [39], while STREs were known to bind osmotic stress-responsive transcription factors [40]. Although prior analyses of the GLP gene family did not explicitly quantify ABREs and STREs, functional categories encompassing these elements were significantly enriched [25,26]. For instance, *DlGLP* promoters in longan exhibited enrichment for methyl jasmonate (MeJA)-response (70 elements), ABA-response (44 ABREs), and drought-inducible elements (35 elements) [25]. Furthermore, hormone-responsive elements and stress-responsive elements were also enriched in peanut [26]. Here, we identified 259 ABREs and 217 STREs in *PutGLP* promoters (Figure 4), indicating potent ABA-mediated regulation and osmotic stress responses. This conservation supports the role of *PutGLPs* in halophytic adaptation in *P. tenuiflora* [23].

### 3.2. Organ-Specific and Stress-Inducible Expression Patterns of PutGLPs

The members of GLP gene family exhibit tissue-/organ-specific expression patterns across diverse plant tissues, indicating their functional specialization for stress adaptation [25]. Approximately half of its 35 *GLPs* are highly expressed in roots in longan, whereas only four *GLPs* show root-specific expression in rapeseed [4,25]. The rice root germin-like protein (*OsRGLP1*) was induced by salinity stress, supporting its potential utility for developing transgenic crops with enhanced stress tolerance [41]. In our study, six *PutGLPs* exhibit root-predominant expression (Figure 5B), implying their involvement in root architecture and ion homeostasis essential for salt exclusion and osmoregulation in halophytes [22,42]. Moreover, five *GLPs* in melon exhibit peak expression in flowers, suggesting their functional significance in key developmental phases such as floral transition and fruit maturation [27]. Moreover, flower-enriched genes may also regulate salt stress responses. In alfalfa (*Medicago sativa*), the flavonol synthase gene *MsFLS13* exhibits flower-enriched expression and enhances saline-alkali stress tolerance [43]. Consequently, seven *PutGLPs* exhibiting both flower-enriched and NaCl-induced expression likely regulate floral reproductive traits and participate in the salt stress response during seed formation.

*GLPs* play a critical role in salt stress resistance across diverse species. Salt stress prolongs the expression of two *HvGLPs* in barley [44], and drives 11 *OsGLP* expression in rice [30]. Furthermore, salt stress induces peanut *AhGLP* expression, and overexpressing *AhGLP2* or *AhGLP3* in *Arabidopsis* enhances its salt tolerance [45]. Our study identified upregulated expression of 15 *PutGLPs* in roots exposed to NaCl stress and in leaves subjected to NaHCO_3_ stress (Figure 5C). Notably, the sustained induction of *PutGLP15* in leaves under both NaCl and NaHCO_3_ stress implies its role in long-term ionic stress adaptation. This functional conservation aligns with the phylogenetically related rice *OsGLP8-12* (Clade VIIa), where stress-responsive differential methylation occurs within promoter *cis*-acting elements, linking epigenetic modification to *OsGLP* regulation under drought and salinity conditions [46]. These results suggest *PutGLPs* play critical roles in plant salt stress response and tolerance.

### 3.3. Functional Significance of PutGLP37 in Salt Stress Adaptation

Notably, *PutGLP37*, identified through salt-tolerant yeast lines [47], was absent from the alkaligrass reference genome assembly, highlighting the challenges of resolving complex genomic regions in halophytes [48,49]. However, this absence sparked our interest in its functional verification. We found that PutGLP37 was localized to the cell wall (Figure 5A and Figure S4), a pattern similar to that observed in peanut AhGLP2/5 and rice OsGLP2-1 [45,50], where it may participate in cell wall remodeling or ROS scavenging [51].

GLPs from various plant species have been reported to exhibit SOD or OXO activities, including rice OsGLP3-7 [12], *Capsicum chinense* CchGLP [52], *C. procera* CpGLP1/CpGLP2 [13], and *R. mucronatum* RmGLP2 [14]. The SOD activity neutralizes superoxide radicals (O_2_^−^) [53], while OXO activity generates H_2_O_2_ [54]. The dual SOD and OXO activities of recombinant PutGLP37 (Figure 6A,B) suggest functional conservation of its enzymatic activity. These imply that the dual SOD and OXO activities of PutGLP37 potentially maintain ROS homeostasis in response to stress conditions [6,12,19,25].

GLP family members exhibit multifunctional roles in coordinating plant growth and mediating defense against abiotic stress [31,55]. CRISPR/Cas9-mediated knockout of *OsGLP1* in rice resulted in UV-B-dependent lesion formation [56]. Under heat stress, rice *osger4* mutant lines exhibit significantly reduced crown root production compared to wild-type rice [57]. Overexpression of potato (*Solanum tuberosum*) *StGLP* boosts heat stress tolerance by elevating H_2_O_2_^−^ triggered ROS scavenging and upregulating antioxidant enzymes in transgenic plants [6]. However, the involvement of *GLPs* in salt stress adaptation is lacking. In this study, overexpression of *PutGLP37* conferred NaCl tolerance in *Arabidopsis*, indicating its role in salt stress regulation (Figure 6C,D). In addition, enhanced survival of *PutGLP37*-overexpressing yeasts and *E. coli* under ionic and alkaline stresses demonstrates its conserved function across kingdoms (Figure S5) [31,56]. Furthermore, the universal presence of ABRE elements in *PutGLP* promoters (Figure 4) suggests that PutGLP37 likely enhances salt tolerance by integrating ABA signaling, potentially through modulating ion homeostasis and ROS scavenging [12,58]. Future studies should investigate PutGLP37’s interactions with salt tolerance pathways (e.g., SOS or ABA) and cell wall components (e.g., pectin or lignin) [59,60,61,62,63,64].

## 4. Materials and Methods

### 4.1. Sequence Search and Identification of PutGLPs

The whole-genome sequence of *P. tenuiflora* was obtained from the Alkaligrass Genome Database V1.0 (http://xhhuanglab.cn/data/alkaligrass.html; accessed on 10 March 2025) [22]. Candidate *GLPs* were initially identified by performing a local BLASTp v2.14 search against the *Arabidopsis* GLP protein database (TAIR, https://www.arabidopsis.org/; accessed on 10 March 2025) by setting a cutoff value of 1 × 10^−100^ for the expected value (e-value). To refine the selection, domain-specific screening was conducted using HMMER 3.0 (http://hmmer.org/; accessed on 10 March 2025), employing the Cupin_1 domain (PF00190) from the Pfam database (http://pfam.xfam.org/; accessed on 10 March 2025) as a query. Candidate proteins containing the Cupin_1 domain were further validated using NCBI-CDD (https://www.ncbi.nlm.nih.gov/cdd/; accessed on 10 March 2025) by setting the e-value to 0.01. Coding sequences (CDS), amino acid sequences, and genomic sequences of the identified *PutGLPs* were extracted for downstream analyses. Notably, *PutGLP37* was isolated from full-length cDNA libraries derived from salt-tolerant yeast strains. MW and theoretical pI of PutGLP proteins were predicted using the ExPASy ProtParam tool (https://web.expasy.org/compute_pi/; accessed on 12 March 2025). Subcellular localization was predicted using the Plant-mPLoc server (http://www.csbio.sjtu.edu.cn/bioinf/plant-multi/; accessed on 12 March 2025).

### 4.2. Phylogenetic and Classification Analysis of PutGLPs

The multiple sequence alignments of GLP proteins among six species, alkaligrass (*P. tenuiflora*), soybean (*G. max*), barley (*H. vulgare*), *Arabidopsis* (*A. thaliana*), rice (*O. sativa*), and maize (*Z. mays*), were initially analyzed using the MAFFT alignment program [65]. The databases include Alkaligrass Genome Database V1.0 (http://xhhuanglab.cn/data/alkaligrass.html; accessed on 10 March 2025), GenBank database (http://www.ncbi.nlm.nih.gov/Entrez/; accessed on 10 March 2025), Phytozome (https://phytozome-next.jgi.doe.gov/info/HvulgareMorex_V3; accessed on 10 March 2025), TAIR 10 (http://arabidopsis.org; accessed on 10 March 2025), RGAP 7 (http://rice.plantbiology.msu.edu/; accessed on 10 March 2025), and Phytozome (https://phytozome-next.jgi.doe.gov/info/Zmays_Zm_B73_REFERENCE_NAM_5_0_55; accessed on 10 March 2025). Subsequently, an unrooted phylogenetic tree was constructed through the Maximum Likelihood (ML) method implemented in FastTree version 2.1.11 by Jones-Taylor-Thornton (JTT) model [66], based on the alignment results. The phylogenetic tree was further refined and visualized using the Interactive Tree Of Life (iTOL) online platform [67].

### 4.3. Gene Structure and Conserved Motif Analysis of PutGLP Proteins

The exon/intron structures of *PutGLPs* were analyzed using the Gene Structure Display Server (GSDS 2.0, http://gsds.gao-lab.org/index.php; accessed on 13 March 2025) [68]. Conserved motifs in PutGLP proteins were identified using the Multiple Expectation Maximization for Motif Elicitation (MEME, https://meme-suite.org/meme/tools/meme; accessed on 13 March 2025), configured to detect a maximum of ten motifs [69].

### 4.4. Analysis of Cis-Acting Elements in PutGLP Promoters

Genomic DNA sequences spanning 3000 bp upstream of the transcription start site for each PutGLP gene family member were retrieved and analyzed for *cis*-acting elements using the PlantCARE online tool (http://bioinformatics.psb.ugent.be/webtools/plantcare/html/; accessed on 14 March 2025) [70]. Promoter *cis*-acting elements associated with plant growth and development, phytohormone regulation, and stress response were selected for detailed analysis.

### 4.5. Chromosomal Distribution and Collinearity Analysis of PutGLPs

Based on the *P. tenuiflora* genome annotation, chromosomal locations of *PutGLPs* (excluding *PutGLP37*) were determined. Their physical positions were mapped to chromosomes using TBtools (v2.322) software [71]. Interspecific collinearity analysis between *P. tenuiflora* and three related species (rice, maize, or soybean) was performed and visualized using TBtools to infer evolutionary relationships.

### 4.6. Plant Material and Abiotic Stress Treatment

Seeds of alkaligrass (*P. tenuiflora*) were germinated hydroponically on gauze-lined baskets under controlled conditions: fluorescent light (200 μmol·m^−2^·s^−1^ intensity, 12 h light/12 h dark cycle), 25 °C daytime and 20 °C nighttime temperatures, and 75% relative humidity for 21 days. Seedlings were subsequently subjected to abiotic stress treatments by exposure to 50 mM Na_2_CO_3_, 200 mM NaCl, or 100 mM NaHCO_3_ for durations of 0 h, 6 h, 12 h, and 24 h. Following treatment, leaf and root tissues were harvested, immediately flash-frozen in liquid nitrogen, and stored at −80 °C. For tissue-specific expression profiling, roots, stems, leaves, sheaths, and flowers were collected from mature plants at the reproductive stage.

### 4.7. RNA Extraction and Real-Time Quantitative PCR (RT-qPCR) Analysis

Total RNA was extracted from alkaligrass (*P. tenuiflora*) tissue samples using TRIzol™ reagent (Invitrogen). Complementary DNA (cDNA) was synthesized from the PrimeScript™ RT Reagent Kit with gDNA Eraser (TaKaRa, Tokyo, Japan). Gene-specific primers were designed using the Primer3 online tool (https://bioinfo.ut.ee/primer3-0.4.0/; accessed on 20 March 2025) and were listed in Table S4. RT-qPCR was performed on an ABI 7500 Real-Time PCR System (Thermo Fisher Scientific, Waltham, MA, USA) using SYBR^®^ Green Master Mix (Vazyme), with the following cycling parameters: initial denaturation at 95 °C for 5 min, followed by 40 cycles of 95 °C for 15 s, 60 °C for 30 s, and 72 °C for 30 s. Relative transcript levels were quantified via the 2^−ΔΔCt^ method [72], normalized to the reference gene *PutActin*. Three biological replicates were analyzed per sample. Heatmaps of relative expression values were generated using TBtools.

### 4.8. Subcellular Localization Analysis

To determine subcellular localization, the CDS of PutGLP37 was amplified using gene-specific primers (Table S4) and subsequently cloned into the *pCAMBIA1300-GFP* expression vector under the control of the CaMV 35S promoter. The recombinant plasmid (*pCAMBIA1300-PutGLP37-GFP*) and the empty vector control (*pCAMBIA1300-GFP*) were separately expressed in *N. benthamiana* leaves via *Agrobacterium tumefaciens*-mediated transient transformation. After 48–72 h incubation, transformed leaf sections were plasmolyzed by immersion in 1 M mannitol solution for 30 min. GFP fluorescence was visualized using confocal microscopy. Three independent biological replicates were performed to confirm localization patterns.

### 4.9. Stress Tolerance Assay of Yeast Transformants Expressing PutGLP37

The CDS of *PutGLP37*, flanked by KpnI/XhoI restriction sites, was amplified and ligated into the *pYES2* expression vector, generating the recombinant plasmid pYES2-PutGLP37. Recombinant plasmids (*pYES2-PutGLP37* and empty *pYES2* control) were transformed into yeast (*S. cerevisiae INVSc*1). Transformed yeast strains were grown in YPD medium. Cultures were then serially diluted to 10^−1^, 10^−2^, 10^−3^, and 10^−4^ times, and spotted onto YPD agar plates supplemented with 1.3 M NaCl, 26 mM NaHCO_3_, and 12 mM Na_2_CO_3_. Untreated YPD plates served as controls. Stress tolerance was assessed by comparing growth between recombinant and control strains. Experiments included three independent biological replicates.

### 4.10. Production and Purification of Recombinant PutGLP37 Protein

The recombinant plasmid *pET32a-PutGLP37* was constructed by amplifying the *PutGLP37* coding sequence and inserting it into the linearized *pET32a* vector using BamHI and SalI restriction sites. The recombinant plasmid was transformed into chemically competent *E. coli* BL21 (DE3) cells. Recombinant strains were cultured overnight in LB medium supplemented with 100 μg/mL ampicillin at 37 °C with shaking (220 rpm). Bacterial cultures were diluted 6-fold in fresh LB medium and incubated at 37 °C until reaching OD_600_ = 0.6–0.8. Protein expression was induced by adding 1 mM isopropyl β-D-1-thiogalactopyranoside (IPTG) under three temperature conditions: 16 °C for 20 h, 28 °C for 10 h, and 37 °C for 4 h. Based on comparative yield analysis, the optimal induction condition (1 mM IPTG at 37 °C for 4 h) was selected for large-scale protein production. His-tagged recombinant proteins were purified using a nickel-nitrilotriacetic acid (Ni-NTA) affinity column, following established protocols.

### 4.11. Salt Stress Tolerance Assay of E. coli Transformants Expressing PutGLP37

To assess salt tolerance, *E. coli* BL21 (DE3) cells harboring *pET32a-PutGLP37* were induced with 1 mM IPTG at 37 °C for 4 h. Cultures were serially diluted to 10^−1^, 10^−2^, and 10^−3^ and spotted onto LB agar plates supplemented with 0, 100, or 250 mM NaCl. *E. coli* BL21 (DE3) carrying the empty *pET32a* vector served as the control. For liquid culture assays, IPTG-induced *E. coli* transformants were inoculated in LB medium containing NaCl at 28 °C for 8 h. Bacterial growth was monitored by measuring OD_600_ at 2-h intervals over an 8-h period. All experiments included four independent biological replicates.

### 4.12. SOD and OXO Activity Assays of PutGLP37

SOD activity was assessed as described previously [73]. Briefly, proteins were separated by semi-native PAGE, and gels were immersed in 0.1 M potassium phosphate buffer (pH 7.8) containing 0.1 mg/mL riboflavin and 20 mg/mL nitroblue tetrazolium (NBT) under dark conditions. After negative staining, gels were rinsed twice with distilled water and exposed to light for 40 min to visualize activity bands. Bovine serum albumin (BSA) served as the negative control. OXO activity of PutGLP37 was analyzed through in-gel activity staining to detect oxalate-dependent H_2_O_2_ production. Horseradish peroxidase (HRP) was used to catalyze the oxidation of 4-chloro-1-naphthol (4CN) dye substrates in an ethanolic system [74]. Recombinant PutGLP37 protein was resolved via non-reducing 10% SDS-PAGE, after which gels were incubated at 25 °C for 1–3 h in a combined solution of substrate (2 mM oxalic acid, 100 mM succinate buffer pH 3.5, and 60% *v*/*v* ethanol) and developing solution (5 U/mL HRP and 0.5 mg/mL 4CN in sodium phosphate buffer pH 5.5). Oxalic acid served as the substrate and glycolic acid as the inhibitor. Experiments were performed in triplicate.

### 4.13. Salinity Tolerance Analysis of PutGLP37-Overexpressing Transgenic Arabidopsis

*A. tumefaciens* strain EHA105 harboring the *pCAMBIA1300-PutGLP37-GFP* plasmid was introduced into *Arabidopsis* Col-0 plants via the floral dip method to generate *PutGLP37*-overexpressing transgenic lines. Transgenic seedlings were selected over three successive generations on half-strength Murashige and Skoog (1/2 MS) agar medium containing 30 μg mL^−1^ hygromycin. Homozygous T_3_ lines were validated for *PutGLP37* expression levels using RT-qPCR with gene-specific primers (Table S4), and the lines exhibiting the highest expression were chosen for salinity tolerance assays. For salt stress analysis, Col-0 and *PutGLP37*-overexpressing seedlings were germinated and grown on 1/2 MS medium either control (0 mM NaCl) or 100 mM, 125 mM, or 150 mM NaCl under controlled conditions (22 °C/16 h light, 20 °C/8 h dark, 75% relative humidity) for seven days. Post-treatment survival rates were quantified to assess salt tolerance. The experiment was performed with three independent biological replicates.

### 4.14. Statistical Analysis

Statistical analyses were performed using GraphPad Prism 6. Data are shown as the means ± SD. Significant differences compared to the control group were determined by Student’s *t*-test, *** *p* < 0.001, ** *p* < 0.01, and * *p* < 0.05.

## 5. Conclusions

This study elucidates the evolutionary expansion and functional diversification of the PutGLP gene family in the halophyte *P. tenuiflora*. The *PutGLPs* exhibit significant expansion driven by tandem duplication on chromosome 7, implying the genomic plasticity underlying stress adaptation in alkaligrass. Collinearity, gene structure, and motif analyses reveal monocot-specific conservation among *PutGLPs*. Stress-responsive *cis*-acting elements and tissue-/organ-specific expression patterns highlight roles of *PutGLPs* in stress adaptation, particularly in root ion homeostasis and floral resilience. Dynamic responses to salt stresses revealed that *PutGLPs* could be involved in distinct signaling pathways for ionic and alkaline stress. Functional characterization demonstrates that cell wall-localized PutGLP37 possesses dual enzymatic activity and is critical for salt adaptation as evidenced by heterologous expression in yeast (*S. cerevisiae INVSc*1), *E. coli*, and transgenic *Arabidopsis*. These findings advance our understanding of GLP multifunctionality in *P. tenuiflora* stress tolerance and provide a foundation for molecular engineering of salt-tolerant crops.

## Data Availability

Data are contained within the article and Supplementary Materials.

## Supplementary Materials

The following supporting information can be downloaded at: https://www.mdpi.com/article/10.3390/plants14152259/s1, Figure S1: Sequence logos depicting conserved amino acid residues across the ten identified protein motifs; Figure S2: Multiple sequence alignment of PutGLPs. The atypical Cupin domain was marked; Figure S3: Multiple sequence alignment of PutGLP37 with orthologous germin-like proteins: OsGLP1-1 (*Oryza sativa*), OsGLP5-2 (*O. sativa*), ZmGLP32 (*Zea mays*), and HvGER6v (*Hordeum vulgare*). The atypical Cupin domain and conserved cysteine residues were marked; Figure S4: Plasmolysis of *Nicotiana benthamiana* cells expressing PutGLP37-GFP. Scale bar = 25 μm; Figure S5: Heterologous expression of PutGLP37 enhances salt stress tolerance in yeast (*Saccharomyces cerevisiae INVSc*1) and *Escherichia coli*. (A) Yeast complementation assay under sodium stress. Recombinant strains harboring *pYES2-PutGLP37* or empty vector (EV, *pYES2*) were serially diluted (10^−1^ to 10^−4^) and spotted on YPD agar plates containing 1.3 M NaCl, 26 mM NaHCO_3_, and 12 mM Na_2_CO_3_. Plates were incubated at 30 °C for 48–72 h. Scale bar = 1 cm. (B) *E. coli* BL21 (DE3) survival assay on Luria-Bertani (LB) solid medium. Transformants expressing *pET32a-PutGLP37* or EV (*pET32a*) were plated with 10-fold serial dilutions (10^−1^ to 10^−3^) under NaCl gradients (0, 100, 250 mM). Scale bar = 1 cm. (C) Growth kinetics of *E. coli* BL21 (DE3) strains in liquid LB medium supplemented with 250 mM NaCl. Growth was monitored at 28 °C for 8 h. Optical density (OD600) was recorded hourly. EV transformants served as negative controls. Data represents mean ± standard deviation (SD) (n = 4, significant differences compared to the control group were determined by Student’s *t*-test, *** *p* < 0.001, ** *p* < 0.01, and * *p* < 0.05); Table S1: Basic characteristics of *GLP* gene family in *Puccinellia tenuiflora*; Table S2: Protein sequences of GLP in soybean (*Glycine max*), barley (*Hordeum vulgare*), *Arabidopsis* (*Arabidopsis thaliana*), rice (*Oryza sativa*), alkaligrass (*Puccinellia tenuiflora*), and maize (*Zea mays*); Table S3: List of *GLP* orthologous gene pairs identified between alkaligrass (*Puccinellia tenuiflora*) and three angiosperm species, rice (*Oryza sativa*), maize (*Zea mays*), and soybean (*Glycine max*); Table S4: Primer sequences were designed for both RT-qPCR analysis of *GLP* gene expression and vector construction of *PutGLP37* in *Puccinellia tenuiflora*.
