# Supplementary material for: Genome-Wide Identification and Salinity Response Analysis of the Germin-like Protein (GLP) Gene Family in Puccinellia tenuiflora
Source: Plants (Basel). 2025 Jul 22;14(15):2259. doi: 10.3390/plants14152259 (PMC12348945; doi:10.3390/plants14152259)
Supplement: Supplementary file 1 [file plants-14-02259-s001.zip › Supplementary Figures S1-S5.pdf]

Motif	E-value	Sites	Width	Logo
1	6.0e-1914	52	50	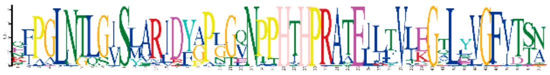
2	9.6e-939	53	29	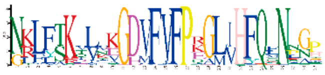
3	1.3e-756	42	29	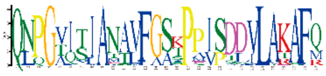
4	8.6e-522	50	21	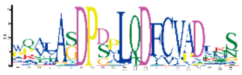
5	1.5e-293	28	15	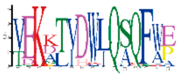
6	3.4e-259	42	11	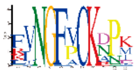
7	1.4e-320	43	21	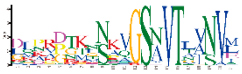
8	9.7e-168	45	11	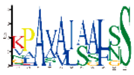
9	4.0e-118	45	8	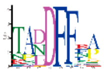
10	1.2e-097	40	12	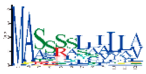

Figure S1. Sequence logos depicting conserved amino acid residues across the 10 identified protein motifs.

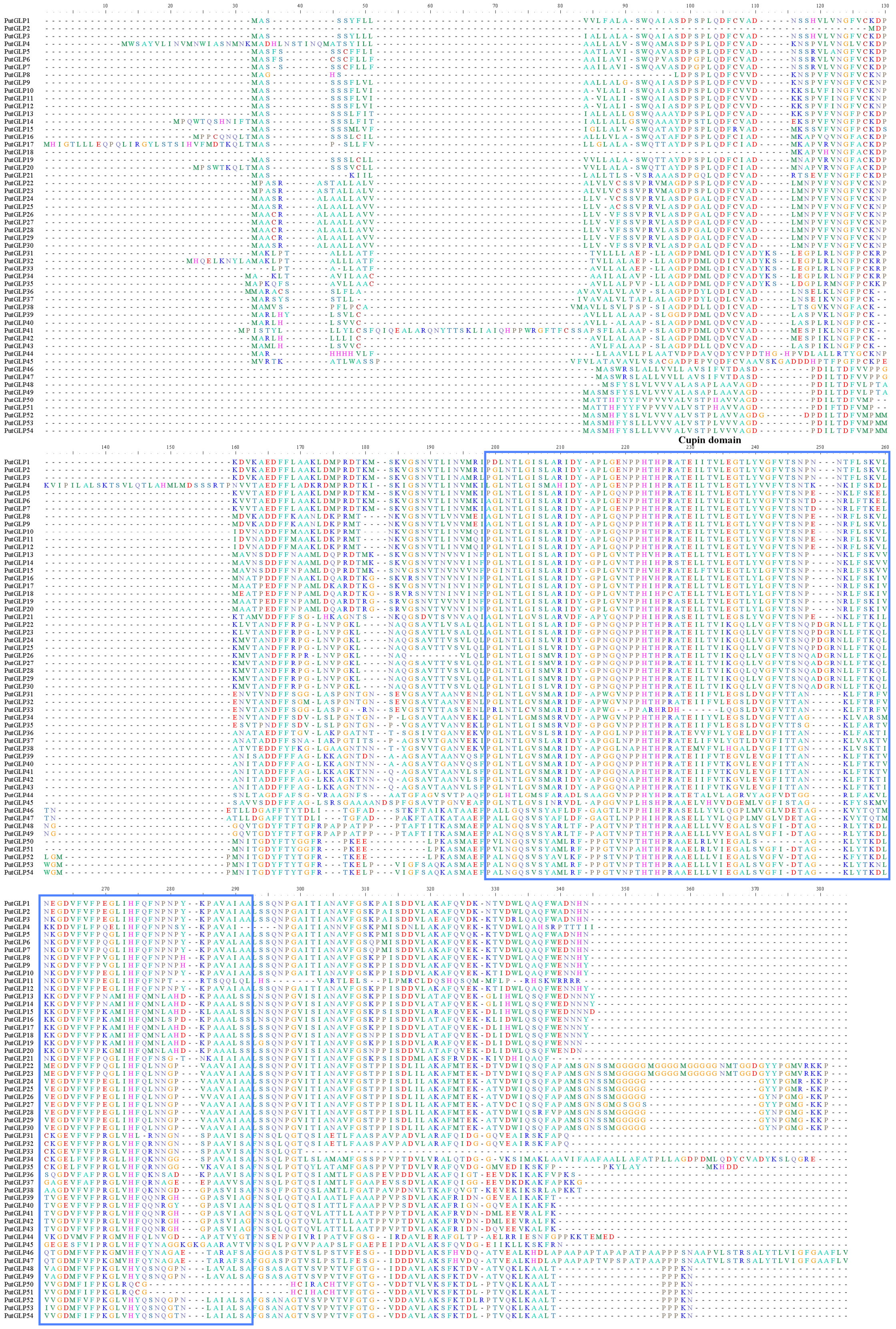

Figure S2. Multiple sequence alignment of PutGLPs. The atypical Cupin domain was marked.

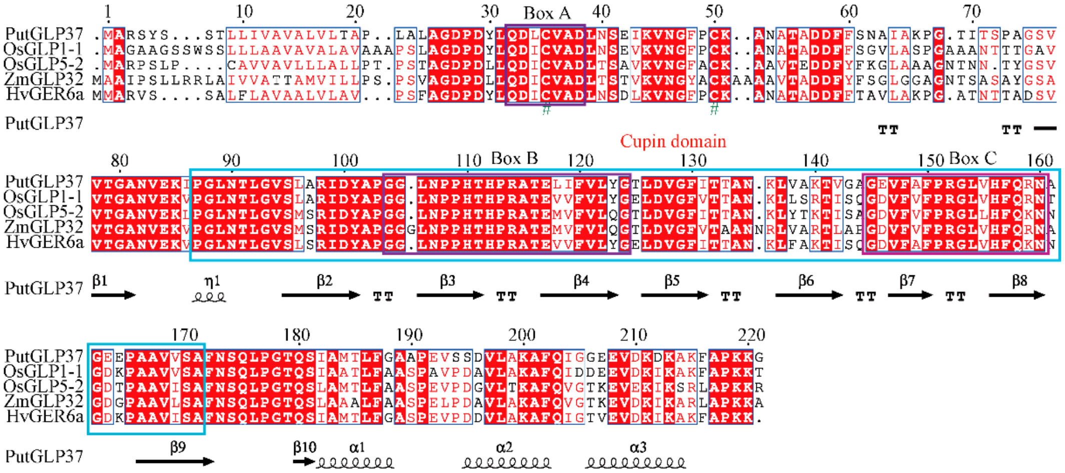

Figure S3. Multiple sequence alignment of PutGLP37 with orthologous germin-like proteins: OsGLP1-1 (*Oryza sativa*), OsGLP5-2 (*O. sativa*), ZmGLP32 (*Zea mays*), and HvGER6v (*Hordeum vulgare*). The atypical Cupin domain and conserved cysteine residues were marked.

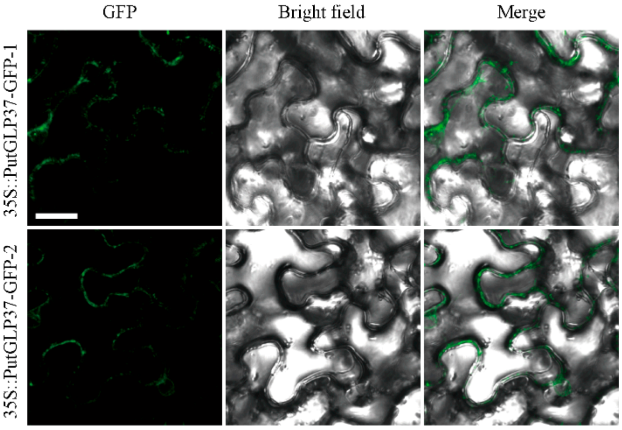

Figure S4. Plasmolysis of *Nicotiana benthamiana* cells expressing PutGLP37-GFP. Scale bar = 25 μm.

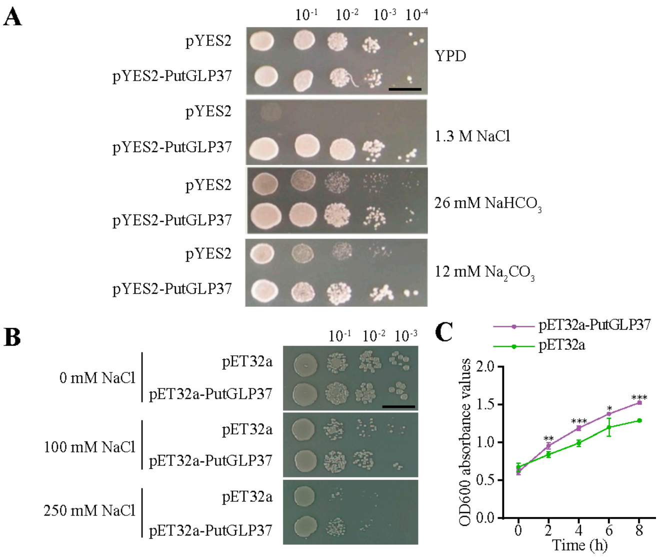

Figure S5. Heterologous expression of *PutGLP37* enhances salt stress tolerance in yeast (*Saccharomyces cerevisiae INVSc*1) and *Escherichia coli*. (A) Yeast complementation assay under sodium stress. Recombinant strains harboring *pYES2-PutGLP37* or empty vector (EV, *pYES2*) were serially diluted (10-1 to 10-4) and spotted on YPD agar plates containing, 1.3 M NaCl, 26 mM $NaHCO_3$, and 12 mM Na_2CO_3. Plates were incubated at 30 °C for 48-72 h. Scale bar = 1 cm. (B) *E. coli* BL21(DE3) survival assay on LB solid medium. Transformants expressing *pET32a-PutGLP37* or EV (*pET32a*) were plated with 10-fold serial dilutions (10-1 to 10-3) under NaCl gradients (0, 100, 250 mM). Growth was monitored after 8 h at 37°C. Scale bar = 1 cm. (C) Growth kinetics of *E. coli* BL21(DE3) strains in liquid Luria-Bertani (LB) medium supplemented with 250 mM NaCl. Optical density (OD600) was recorded hourly. EV transformants served as negative controls. Data represent mean ± SD (n=4, significant differences compared to the control group were determined by Student's *t*-test, ****P* < 0.001, ***P* < 0.01, and **P* < 0.05).
