# Supplementary material for: Genome-Wide Identification and Salinity Response Analysis of the Germin-like Protein (GLP) Gene Family in Puccinellia tenuiflora
Source: Plants (Basel). 2025 Jul 22;14(15):2259. doi: 10.3390/plants14152259 (PMC12348945; doi:10.3390/plants14152259)
Supplement: Supplementary file 1 [file plants-14-02259-s001.zip › Table S1.pdf]

Table S1. Basic characteristics of *GLP* gene family in *Puccinellia tenuiflora*.

Gene name	Gene ID	Number of amino acids	Theoretical pI	Molecular weight (kDa)	Subcellular localization prediction
PutGLP1	Pt_Chr0205171	226	5.07	24.58	Cell wall
PutGLP2	Pt_Chr0205214	179	5.47	19.52	Cell wall
PutGLP3	Pt_Chr0205170	226	5.67	24.40	Cell wall
PutGLP4	Pt_Chr0402444	280	8.43	30.91	Cell wall
PutGLP5	Pt_Chr0207227	229	6.89	24.84	Cell wall
PutGLP6	Pt_Chr0205169	229	5.51	24.95	Cell wall
PutGLP7	Pt_Chr0205212	228	5.51	24.76	Cell wall
PutGLP8	Pt_Chr0402443	207	6.58	22.60	Cell wall
PutGLP9	Pt_Chr0402446	225	6.50	24.38	Cell wall
PutGLP10	Pt_Chr0402451	224	6.41	24.49	Cell wall
PutGLP11	Pt_Chr0402445	210	9.51	23.53	Cell wall
PutGLP12	Pt_Chr0402447	224	6.41	24.48	Cell wall
PutGLP13	Pt_Chr0704422	228	5.90	24.69	Cell wall
PutGLP14	Pt_Chr0704423	240	5.58	26.20	Cell wall
PutGLP15	Pt_Chr0704421	227	5.65	24.85	Cell wall
PutGLP16	Pt_Chr0704687	235	6.81	25.53	Cell wall
PutGLP17	Pt_Chr0704688	257	6.64	28.13	Cell wall
PutGLP18	Pt_Chr0704689	190	5.90	20.80	Cell wall
PutGLP19	Pt_Chr0704690	225	5.74	24.39	Cell wall
PutGLP20	Pt_Chr0704691	234	5.24	25.39	Cell wall
PutGLP21	Pt_Chr0502264	215	7.80	23.04	Cell wall
PutGLP22	Pt_Chr0301173	268	8.49	27.58	Cell wall
PutGLP23	Pt_Chr0301199	268	8.49	27.58	Cell wall
PutGLP24	Pt_Chr0301174	249	8.84	26.00	Cell wall
PutGLP25	Pt_Chr0301200	249	8.84	25.97	Cell wall
PutGLP26	Pt_Chr0700805	241	8.86	25.33	Cell wall
PutGLP27	Pt_Chr0700807	249	8.38	25.90	Cell wall
PutGLP28	Pt_Chr0700806	249	8.86	25.98	Cell wall
PutGLP29	Pt_Chr0700827	249	8.49	25.91	Cell wall
PutGLP32	Pt_Chr0604736	235	5.54	25.04	Cell wall
PutGLP33	Pt_Chr0604730	171	7.00	18.33	Cell wall
PutGLP34	Pt_Chr0403036	260	5.58	27.19	Cell wall

PutGLP35	Pt_Chr0605775	234	6.41	24.84	Cell wall
PutGLP36	Pt_Chr0604084	221	5.58	23.09	Cell wall
PutGLP37	-	221	5.60	22.86	Cell wall
PutGLP38	Pt_Chr0501398	222	7.72	23.07	Cell wall
PutGLP39	Pt_Chr0402211	216	6.50	22.51	Cell wall
PutGLP40	Pt_Chr0603689	214	9.30	22.33	Cell wall
PutGLP43	Pt_Chr0106251	214	6.95	22.45	Cell wall
PutGLP44	Pt_Chr0206684	228	6.79	24.09	Cell wall
PutGLP45	Pt_Chr0105553	235	5.71	24.27	Cell wall
PutGLP46	Pt_Chr0700256	248	4.51	25.82	Cell wall
PutGLP47	Pt_Chr0700445	248	4.57	25.79	Cell wall
PutGLP48	Pt_Chr0700255	208	6.25	21.41	Cell wall
PutGLP50	Pt_Chr0700250	173	8.43	18.86	Cell wall
PutGLP51	Pt_Chr0700437	179	7.05	19.44	Cell wall
PutGLP52	Pt_Chr0700439	207	6.04	21.96	Cell wall
PutGLP53	Pt_Chr0700249	210	6.90	22.33	Cell wall
PutGLP54	Pt_Chr0700435	210	6.39	22.35	Cell wall
