# Supplementary material for: Genome-Wide Identification and Salinity Response Analysis of the Germin-like Protein (GLP) Gene Family in Puccinellia tenuiflora
Source: Plants (Basel). 2025 Jul 22;14(15):2259. doi: 10.3390/plants14152259 (PMC12348945; doi:10.3390/plants14152259)
Supplement: Supplementary file 1 [file plants-14-02259-s001.zip › Table S2.pdf]

Table S2. Protein sequences of GLP in soybean (*Glycine max*), barley (*Hordeum vulgare*), Arabidopsis (*Arabidopsis thaliana*), rice (*Oryza sativa*), alkaligrass (*Puccinellia tenuiflora*), and maize (*Zea mays*).

Species	Gene name	Gene ID	Protein sequences
Glycine max	GmGER1	EU916269	MKLTGFLQAVTLALVLATVSASDPDPLQDLCVADLASAVKVNGFTCKDAAKVNASDFFSDILAKPGA<br>TNNTYGSLVTGANVQKIPGLNTLGVSLSRIDYAPGGINPPHTHPRATEVVFVLEGTLDVGFITTANVLI<br>SKSISKGEIFVFPKGLVHFQKNNGKEQASVIAAFNSQLPGTQSIALTLFAATPPVPDNVLTKAFQVGTK<br>EVEKIKSRLAPKK
	GmGER2	EU916250	MKVVYLLVALLALTSTVVSAYDPSPLQDFCVAAKEKDGVFVNGKFCKDPKLVKAEDFFRHVEPGKT<br>DNPLGSNVTQVFVDQLPGLNTLGIGLARIDFAPKGLNAPHTHPRGTEILIVLEGTLYVGFVTSNQDGN<br>RLFTKVLNKGDVFVFPIGLIHFQLNVGYGNAVAIAGLSSQNPGAITIANALFKANPPISSEVLTKAFQV<br>DKSIIDYLQKQSWYDNNN
	GmGER3	EU916251	MKVVYFLVAILALTSTVVSAYDPSPLQDFCVAAKEKDGVFVNGKFCKDPKLVKAEDFFKHVEPANTA<br>NPLGSQVTPVFVDQLPGLNTLGISLARIDYAPKGLNPPHTHPRGTEILIVLEGTLYVGFVTSNQDGNRL<br>FTKVLNKGDVFVFPIGLIHFQLNVGYGNAVAIAGLSSQNPGTITIANALFKANPPISPEVLTKAFQVDK<br>STIDYLQKQSWYDNNN
	GmGER4	EU916252	MKVVYFLVAILALASSLVSAHDPSPLQDFCVATKERDGVYVNGKFCKEPKDVKAEDFYKEVEPGNPS<br>NQLGSAVTPVFVDQLPGLNTLGLSLARIDYESMGLNPPHIHPRATEIIIVLEGILLVGFATSNQDGNRLF<br>SKMLKKGDVFVSPMGLIQFQYNPGRGRAVSISAFSSQNPGTVTVANAVFRSNPRISTDILTKSFQVDK<br>KVIDELQNQN
	GmGER5	EU916253	MRTHFHSLPLIFLFTLSLLLGKTRPDPDPLQDYCVADSKSEFFINGVPCIDPDKVSSSHFVTSALSKTGN<br>TSNQFGFSVTATTTANLPGLNTLGLVLVRVDIAGNGIVPPHSHPRASEVTTCLKGMLLVGFVDTSNRV<br>FTQNLRPGESFVFPKGLVHFLFNSDSREPAIAISGLNSQNPGAQIASLATFASKPPIPDDILKKAFQISKG<br>EVETIRRNLGG
	GmGER6	EU916254	MKVVYLFVVLMALASSVAFAYDPSPLQDFCVAINDTKAGGLYGVFVNGKFCKDPKFAYADDFFFGG<br>LGPGDTANAQGSKVTAVTVNEILGLNTLGISLARIDFAPKGLNPPHTHPRGTEILVVLEGTLYVGFVAS<br>NQNDNRLFTKVLYKGDVFVFPIGLIHFQQNVGYGNAVAIAALSSQNPGVITIANAVFGSKPPISDEVLA<br>KAFQVDKNIVDYLQKQFGYNNKVNREHN
	GmGER7	EU916255	MKPFAPFFFMLLFLEAFSNIQVCLGDCDNLQDTCPAVPPNKQTIFINGLQCKNPVNVTAQDFRTTELSK<br>AGPTDIFGASLKIVSAAEFNGLNTLGLSIGRIDLDGNGLVNFHYHPRATEIIFVTKGVLLAGFVDTKNQ<br>FFQKFLKVGDVFVFPKALFHFCLNTGFEESTVFSVYNSQNPGFVSLSPTTFDTTLESLDKIKKRLMSLS<br>ASEA

GmGER8	EU916256	MFLHIVFLLFLLSSTSHATVQDFCVADLKGADTPAGYPCKPPANVTSDDFVYTGLAEAANVTNIINAA<br>VTPAFVAQFPGLNGLELSAARLDLGPSGVIPLHTHPGANELLIVLQGHILAGFISSGNIVYQKVLKKGE<br>LMVFPQGLLHFQIAVGKRKALAFPVFSSANPGLQILDFALFASNFSTPLVTQTTFLDPDLVKKLKGVLG<br>GSG
GmGER9	EU916257	MKMIHFLFLFALVSFTSHASVNDFCVADLKGPDSPSGYQCKPPNTVTVDDFVFSGFVAGNTTNTFNA<br>ALTSAFVTDFPGVNGLGVSAARLDIAKGGSIPMHTHPAATELLIMVEGQITAGFMTPTALYTKTLKPG<br>DIMVFPQGQLHFQVNSGNGKATAFLAFSSANPGAQLLDLLLFGNTLPSDLVAQTTFLDVAQVKKLKA<br>RFGGRG
GmGER10	EU916258	MKKMILTLFFNIILSLLSLSHASVVDFCVADYTGPNGPAGYSCKSPAKVTVDDFVYSGLGTAGNTSNII<br>KAAVTPAFDAQFPGVNGLGISIARLDLAAGGVIPLHTHPGASELLVVVQGTICTGFVASDNTVYLKTL<br>KKGDVMVYPQGLLHFQINDGESQALAFVSFSSANPGLQILDFSLFKSDFPTELITQTTFIDAAVVKKLK<br>GVLGGSG
GmGER11	EU916259	MKVFYFFVVLLALASSVAFGYDPSPLQDFCVAINDTKTGGLYGVFVNGKFCKDPKFAYADDFFFGGL<br>GPGNTANPQGSKVTAVTVNEILGLNTLGISLARIDFAPKGLNPPHTHPRGTEILVVLEGTLYVGFVASN<br>QNDNRLFTKVLYKGDVFVFPIGLVHFQQNIGYGNAVAIAGLSSQNPGVITIANAVFGSKPPISDEVLAK<br>AFQVDKNVIDYLQKQFGYNNKVNGKHN
GmGER12	EU916260	MKVVYFFVVLLALASSIAFAYDPSPLQDFCVAINDTKTGVFVNGKFCKDPKLAKAEDFFFPGLGPGN<br>TSNPLGSKVTAVTVNEILGLNTLGISLARIDFAPKGLNPPHTHPRGTEILVVLEGTLYVGFVASNQNDN<br>RLFTKVLNKGDVFVFPIGLIHFQQNIGYGNALAIAGLSSQNPGVITIANAVFGSKPPISDEVLAKAFQV<br>GKNVIDYLQKQFWYNNS
GmGER13	EU916261	MKVVYFLVVILALTSSVVSAYDPSPLQDFCVAAKEKDGVFVNGKFCKDPKLVKAEDFFRHVEPGKTD<br>NPVGSNVTQVFVDQLPGLNTLGIALARIDFAPKGLNAPHTHPRGTEILIVLEGTLYVGFVTSNQDGNR<br>LFTKVLNKGDVFVFPIGLIHFQLNVGYGNAAAIAALSSQNPGAITIANALFKANQPISSEVLTKAFQVD<br>KSIIDYLQKQSWYDNNN
GmGER14	EU916262	MKVVYFLVAILALTSSVVSAYDPSPLQDFCVAAKEKDGVFVNGKFCKDPKLVKAEDFFRHVEPGKTD<br>NPVGSNVTQVFVDQLPGLNTLGIALARIDFAPKGLNAPHTHPRGTEILIVLEGTLYVGFVTSNQDGNR<br>LFTKVLNKGDVFVFPIGLIHFQLNVGYGNAAAIAALSSQNPGAITIANALFKANPPISSEVLTKAFQVD<br>KSIIDYLQKQSWYDNNN
GmGER15	EU916263	MKLFAHFFFMLLFLVAFSNIQVCLGDCDNLQDTCPAVPPNKQTIFINGLQCKNPVNVTAQDFRTTELS<br>KTGPRDIFGASLKIVSAAEFIGLNTLGLSIGRTDLDGNGLVNFHYHPRATEIIYVTKGVLLAGFVDTKN<br>QYFQKFLKVGDVFVFPKALFHFFLNTDFEEATVFSVYNSQNPGFVSLSPTTFDTTLESLDKIKKRLISL<br>SASEAQAQAQDVNSFISPELETIYS

	GmGER16	EU916264	MSPTNNTFAIFVIFCCAISFAFASDPDTLQDLCVALPSSGVKVNGFACKAESNVTEADFFFAGLAKPGV<br>INNTVGSVVTGANVEKIPGLNTLGVSFSRIDYKAEGLNPPHTHPRATEIVFVLDGQLDVGFITTANKLI<br>SKSIKKGEIFVFPKGLVHYQKNNGDKPASVLSAFNSQLPGTVSIAAALFTSTPTVPDDVLAQAFQIDTK<br>KVDDIKAKLAPKKT
	GmGER17	EU916265	MSPTNTFAILVFFCCAISFTFASDPDTLQDLCVALPSSGVKVNGFACKAESNVTEADFFFAGLAKPGAT<br>NNTLGSVVTAANVDKIPGLNTLGVSFSRIDYKAGGLNPPHTHPRATEIVFVLDGQLDVGFITTANKLIS<br>KSINKGEIFVFPKGLVHYQKNNGDKPASVLSAFNSQLPGTVSIAATLFTSTPTVPHNVLSQAFQIDAKL<br>VDDIKAKLAPKKT
	GmGER18	EU916266	MINKVAVLLVVLLFIIAALASDPDPVMDFCIAKSPDNSFSCKNSSTATVEDFTYSGIKSPGNFKQTGFSS<br>MAVNSNVFPGLNTLGVSFVRADFGVGGVNVPHFHPRATEVAFVLEGKIYSGFVDTNNKVFAKVLEK<br>GEVMVFPRGLVHFQMNVGDRPATILGSFDSQNPGLMRIPNAVFGSDIKEELLEKAFGLSSKELSKLKK<br>REAPMAAIRGKTCACFLPLFTHCLVIH
	GmGER19	EU916267	MKMANLLLLVTLAQLLATAISSDPDLLQDLCVADLASGVKVNGFTCKEASKVNASDFFSNILAKPGA<br>TNNTFGSLVTGANVQKVPGLNTLGVSLSRIDYAPGGINPPHTHPRATELVFVLEGQLYVGFITTSNVLI<br>SKTINKGDIFVFPKGLLHFQKNNAKVPAAVISAFNSQLPGTQSTATTLFAATPSVPDHVLTQTFQVGTK<br>EVQKIKSRLAPKK
	GmGER20	EU916268	MKVVYFLVAILALTSSLVSAYDPSPLQDFCVAAKEKDGVFVNGKFCKDPKLVKAEDFFRHVEPGKTD<br>NPVGSNVTQVFVDQLPGLNTLGIALARIDFAPKGLNAPHTHPRGTEILIVLEGTLYVGFVTSNQDGNR<br>LFTKVLNKGDVFVFPIGLIHFQLNVGYGNAVAIAGLSSQNPGAITIANALFKANPPISSEVLTKAFQVD<br>KTIIDYLQKQSWYDNNN
	GmGER21	EU925816	MVPTAVKVNGFTCKDAAKVNASDFFSDILAKPGATNNTYGSLVTGANVQKIPGLNTLGVSLSRIDYA<br>PGGINPPHTHPRATEVVFVLEGTLDVGFITTANVLISKSISKGEIFVFPKGLVHFQKNNGKEQASVIAAF<br>NSQLPGTQSIALTLFAATPPVPDNVLTKAFQVGTKEVEKIKSRLAPKK
Hordeum vulgare	HvGER2a	ABG46233.1	MANAMLLPVLISFLVLPFSAMALTQDFCVADLSCSDTPAGYPCKTGVGAGDFYYHGLAAAGNTSNLI<br>KAAVTPAFVGQFPGVNGLGISAARLDIAVGGVVPMHTHPAASELLFVTEGTILAGFISSSSNTVYTKTL<br>YKGDIMVFPQGLLHYQYNGGSSSAVALVAFSGPNPGLQITDYALFANNLPSAVVEKVTFLDDAQVKK<br>LKSVLGGSG
	HvGER5a	ABG46237.1	MARLHLSAVAACAVLLALAAPSLAGDPDMLQDVCVADLKSPIKLNGFPCKADITADDFFFAGLKNAG<br>NTNNPAGSNVTAANVQSFPGVNTLGVSMARIDYAPGGQNPPHTHPRATEIIFVLEGVLEVGFITTANK<br>LFTKTITVGDVFVFPRGLVHFQQNRGHGPAAVIAGFNSQLQGTQAIATTLFAAAPPVPSDVLAKAFRV<br>SNEDIDAVKARFK
	HvGER6a	ABG46238.1	MMARVSSALFLAVAALVLAVPSFAGDPDYLQDICVADLNSDLKVNGFPCKANATADDFFTAVLAKPG

			ATNTTADSVVTGANVEKVPGLNTLGVSLSRIDYAPGGLNPPHTHPRATEVVFVLYGELDVGFITTANK<br>LFAKTISQGDVFAFPRGLVHFQKNNGDKPAAVISAFNSQLPGTQSIAMTLFGASPEVPDDVLAKAFQIG<br>TVEVDKIKAKFAPKK
	HvGER1a	ABG46232.1	MGYSKNLGAGLFTMLLLAPAIMATDPDPLQDFCVADLDGKAVSVNGHTCKPMSEAGDDFLFSSKLT<br>KAGNTSTPNGSAVTELDVAEWPGTNTLGVSMNRVDFAPGGTNPPHIHPRATEIGMVMKGELLVGILG<br>SLDSGNKLYSRVVRAGETFVIPRGLMHFQFNVGKTEAYMVVSFNSQNPGIVFVPLTLFGSNPPIPTPVL<br>TKALRVEAGVVELLKSKFAGGS
	HvGER3a	ABG46234.1	MASSSTLFCLSALLVLASWQATAYDPSPLQDFCIADMKAPVRVNGFACKDPMAATPEDFFNAAMLD<br>QPRDTKASKVRSNVTNINVINFPGLNTLGISLARIDYGPLGVNTPHIHPRATELLTVLEGTLYLGFVTS<br>NPNRLFSKIVKKGDVFVFPKAMIHFQMNLAHDKPAAALSSLSSQNPGVISIANAVFGSKPPISDDVLAT<br>AFQVEKDLIHWLQSQFWENNNY
	HvGER4b	-	MSSSSSFLVLASLLALVSWQATASDPSPLQDFCVADMHSPVRVNGFVCKNPMEVNADDFFKAANLD<br>KPRMTNKVGSNVTLINVMQIAGLNTLGISIARIDYAPLGQNPPHTHPRATEILTVLEGTLYVGFVTSNL<br>PAPNRNKFLSKVLNKGDVFVFPVGLIHFQFNPNPHKPAVAIAALSSQNPGAITIANAVFGSNPPISDDVL<br>AKAFQVEKNTIDWLQAQFWENNHN
	HvGER4c	ABG46235.1	MASSPTFLLLVALFALISWQAVASDPGPLQDFCVADMHSPVRVNGFVCKNPMDVNADDFFKAAALD<br>KPRVTNKVGSNVTLINVMQIAGLNTLGISIARIDYAPLGQNPPHTHPRATEILTVLEGTLYVGFVTSNLP<br>APNRNKFLSKVLNKGDVFVFPMGLIHFQFNPNPHQPAVAIAALSSQNPGAITIANAVFGSDPTISDDVL<br>AKAFQVEKNTIDWLQAQFWENNQN
	HvGER4d	ABG46236.1	MASSCSFLLLAALLALVSWQATSSDPSPLQDFCVADMHSPVRVNGFVCKNPMDVNADDFFKAAALD<br>KPRVTNEVGSNVTLINVMQIAGLNTLGISIARIDYAPLGQNPPHTHPRATEILTVLEGTLYVGFVTSNLP<br>APNRNKFLSKVLNKGDVFVFPVGLIHFQFNPNPHQPAIAIAALSSQNPGAITIANAVFGSDPAISDDVL<br>AKAFQVEKNTIDWLQAQFWENNHN
Arabidopsis thaliana	AtGLP1-1	AT1G02335.1	MMNSRISIIIALSCIMITSIRAYDPDALQDLCVADKSHGTKLNGFPCKETLNITESDFFFAGISKPAVINST<br>MGSAVTGANVEKIPGLNTLSVSLARIDYAPGGLNPPHTHPRATEVVYVLEGELEVGFITTANKLFTKTI<br>KIGEVFVFPRGLVHFQKNNGKSPASVLSAFNSQLPGTASVAATLFAAEPALPEDVLTKTFQVGSKMVD<br>KIKERLATKK
	AtGLP1-2	AT1G09560.1	MASPTLTLLLLLTTVSFFISSSADPDMLQDLCVADLPSGIKINGFPCKDAATVTSADFFSQGLAKPGLTN<br>NTFGALVTGANVMTIPGLNTLGVSLSRIDYAPGGLNPPHTHPRATEVVFVLEGTLDVGFLTTANKLIS<br>QSLKKGDVFAFPKGLVHFQKNNGDVPASVIAAFNSQLPGTQSLGATLFGSTPPVPDNILAQAFQTSPG<br>TVKHIKSKFQPKK
	AtGLP1-3	AT1G10460.1	MILNILLTLTLLMGRVKSDPDPLQDYCVSPPPSSHQQIFLNGKLCKDPTQASVSDFSTSALSRPGNTKT

		KPFMINVTVTTTANLPGLNTVGLTMARLDFGGSGVVPPHVHPRASEVTVCLDGVLLVGFVDTSGRVFTQELHPGETFVFPKGLIHFLYNIDTVSSALAVSGLSSQNPGTQIVSLSSFISKPPFLVEVLKSAYDINGQDVARIRKSLEG
AtGLP1-4	AT1G18970.1	MATLQIPSALLRSFLLMFCLFVIPSLSSDSDPLQDFCVGDLKASASINGFPCKSAVSASDFFYSGLGGPLDTSNPNGVTVAPANVLTFPGLNTLGISMNNVELAPGGVNPPHLHPRATEVGTVIEGSVFVGFLSTNNTLFSKVLNAGEAFVIPRGLVHFQWNVGQVKARMITAFNSQLPGAVVLPSTLFGSKPEIPNAVLTRAFRTDDTTVQNLKSKFAV
AtGLP1-5	AT1G18980.1	MTTLQISSSLFRSFLLVICVFVIPSLSSDSDPLQDFCVGDLKASPSINGFPCKSSVSASDFFFSGLGGPLNTSTPNGVAVSPANVLTFPGLNTLGLSMNNVEFAPGGVNPPHSHPRATEAGVVIEGSVFVGFLTTNNTLFSKVLNAGEMFVVPRGLVHFQWNVGKVKARLITSFNSQLPGSAVLPSTLFGSNPTIPNAVLTKTFRTDDVTVNKLKSKFAV
AtGLP1-6	AT1G72610.1	MLRTIFLLSLLFALSNASVQDFCVANLKRAETPAGYPCIRPIHVKATDFVFSGLGTPGNTTNIINAAVTPAFAAQFPGLNGLGLSTARLDLAPKGVIPMHTHPGASEVLFVLTGSITAGFVSSANAVYVQTLKPGQVMVFPQGLLHFQINAGKSSASAVVTFNSANPGLQILDFALFANSLPTELVVGTTFLDATTVKKLKGVLGGTG
AtGLP1-7	AT1G74820.1	MAHISQISSFLSIVLIFLALCITLFTNPTLSLPALKLNPFQDFCVADLQATPTNSGYPCKSQVTSEDFFYSGLNTPLNTSNPKGIAANPANLLTFPGLNTLGISMYNVAIAPGGYNQPHSHPGVTEAGVVIEGSVLVGFLTTNYTLYSKVIGPGDMFVIPPGLIHYEGNVGKTQCRLLTVVADDLPSEVGVPHTLLATKPAIPNEVLISAFKADSKTINMLRSKFTA
AtGLP3-1	AT3G04150.1	MKGLVQFLVAKIILLVLASTFVHCYDPNPLQDYCVATNGTNRVFVNGKFCKDPKLVTANDFFYSGLNIPGNTSNRLGASVTDVDVRRIPGLNTLGIAIARLDFAPGGQLPPHIHPRASQIILVLKGQLSVGFVSSNDYNYTLFSKILYPGDVFAFPIGLVQFHANTGKTHAVAIGVVGSQDPGVIPIGDAVFGSNPLIDPKLLAKAFALDVNIVRHVQRVFSSEGYIVK
AtGLP3-2	AT3G04170.1	MKYPFQCFLAKIILLALASSFVSCYDPSPLQDYCVAVPEKNGVFVNGEFCKDPKLVTSDDFFASGLNIPGNTNKRLGSFVNPANIPGLNTLGVGIARIDFAPGGLIPPHIHPRASEIILVIKGKLLVGFVSSNDYNYTLFSKILYPGDVFVHPIGLVQFHANIGKTNAVAIGAVGSQNPGYISVGDAVFGSKPPIDPKILAKAFALDINIVRYLRKVFSPQDDIVND
AtGLP3-3	AT3G04180.1	MEGLLQFLLAKIILLALASSFVYCYDPSPLQDYCVATNETNGVYVNGEFCKDPKRVTTNDFYTSGLNVPGNTIIGPGARNTVVDVERLPGLNTLGVDIARYDFAPGGLDPPHTHPRGSQIFLVMKGKLFVGFVSSNEYNYTLFTKVLYPGDVFVFPKGLIHFHANIGETNAVVISAGGSQDPGRIIIGDAVFGSKPLIDPKVLAKAFALDYNKVKYLQAVFS
AtGLP3-4	AT3G04190.1	MKGLLHFLLAKIILLALASSFVYCYEPSPLQDYCVATNETNGVYVNGKFCKDPKCVTANDFYTSGLN

		VPGNTSTGPGVKITVVDVKRMPGLNTLGVDIARIDFAPGGLYPPHTHPRGSEIFLVMKGKLFVGFVSS<br>NEYNYTLFTKVLYPGDVFVFPKGLIQFHANIGKTNAVVIAATGSQNPGRIIIGNAVFGSKPLIDPKVLA<br>KAFALDFNKVKYFQAVFS
AtGLP3-5	AT3G04200.1	MMEVLLRLLVTQVILLALATSFVSCYDPNPLQDFCVAASETNRVFVNGKFCKDPKSVTANDFSYSGL<br>NIARNTTNFLGSNVTTVDVNKIPGLNTLGVSLARLDFAQGGQNPPHIHPRATEILVVTKGKLLVGFVS<br>SNQDNNRLFYKVLKRGDVFVFPIGLIHFQMNVRRTRAVAFAGFGSQNPGTIRIADAVFGSNPSIPQEVL<br>AKAFQLDVKLVRFLHIVFGPPLW
AtGLP3-6	AT3G05930.1	MATSMIPIFVTFMLVAAHMALADTNMLQDFCVADLSNGLKVNGYPCKDPAKVTPEDFYFIGLATAAA<br>TANSSMGSAVTGANVEKVPGLNTLGVSISRIDYAPGGLNPPHLHPRASEAIFVLEGRLFVGFLTTTGKL<br>ISKHVNKGDVFVFPKALLHFQQNPNKAPASVLAAFDSQLPGTQVVGPSLFGSNPPIPDDLLAKAFGA<br>AAPEIQKIKGKFPPKK
AtGLP3-7	AT3G05950.1	MEGFLRFLVAKAILLALASSFVSCYDPSPLQDFCVAVDDASGVFVNGKFCKDPKYVKAEDFFTSGLNI<br>AGNTINRVGSNVTNVNVDKIPGLNTLGVSLVRIDFAPGGQNPPHTHPRATEILVVVEGTLLVGFVTSN<br>QDNNRLFSKVLYPGDVFVFPIGMIHFQVNVGRTNAVAFAGLGSQNPGTITIADAVFGSKPSIMPEILAK<br>AFQLDVNVVKYLEARFSSNYDRHY
AtGLP3-8	AT3G10080.1	MEANTLFLLKALCLLCFNVCFTLASDPDPIQDFCIPKPVTSPYHDHHFSTNLPCKNSSEVTTEDFVFSG<br>LKTAGNFTETGFATVPVGPENFPGLNTLGISFVRADLKPGSINPPHYHPRATEVAHLVKGRVYSGFVDS<br>NNKVYAKVMEEGEMMVYPKGLVHFQMNVGDVTATIVGGLNSQNPGIQKIPSVVFGSGINEELLMKA<br>FGLSLKQIGTLKKRFDPVMSNEH
AtGLP3-9	AT3G62020.1	MDSRCFGFFFTLLSLNVIVLAYDPDTLQDLCVADRTSGIKVNGFTCKPESNITASDFFFAGIGKPAVVN<br>NTVGSAVTGANVEKIAGLNTLGVSLARIDYAPGGLNPPHTHPRATEVIFVLEGELDVGFITTANKLFA<br>KTVKKGEVFVFPRGLIHYQKNNDKAKPASVISAFNSQLPGTQSIAATLFTATPAIPDHVLTTTFQIGTKE<br>IEKIKSKFAPKKV
AtGLP4-1	AT4G14630.1	MTIKSLSFLAALSLFALTLPLVIASDPSPLQDFCVGVNTPADGVFVNGKFCKDPRIVFADDFFFSSLNRP<br>GNTNNAVGSNVTTVNVNNLGGLNTLGISLVRIDYAPNGQNPPHTHPRATEILVVQQGTLLVGFISSNQ<br>DGNRLFAKTLNVGDVFVFPEGLIHFQFNLGGTPAVAIAALSSQNAGVITIANTIFGSKPDVDPNVLARA<br>FQMDVNAVRNLQARF
AtGLP5-1	AT5G20630.1	MKMIIQIFFIISLISTISFASVQDFCVADPKGPQSPSGYSCKNPDQVTENDFAFTGLGTAGNTSNIIKAAV<br>TPAFAPAYAGINGLGVSLARLDLAGGGVIPLHTHPGASEVLVVIQGTICAGFISSANKVYLKTLNRGDS<br>MVFPQGLLHFQLNSGKGPALAFVAFGSSSPGLQILPFALFANDLPSELVEATTFLSDAEVKKLKGVLG<br>GTN
AtGLP5-2	AT5G26700.1	MASFATHLVVVVTMLFVAMASAEMLQDVCVADLSNAVKVNGYTCKDSTQITPEDFYFKGLANIAAT

		NTSTGSVVTGANVEKLPGLNTLGLSMSRIDYAPNGLNPPHVHPRASEIIFVLEGQLYVGFVTTAGKLI<br>AKNLNKGDVFTFPKGLIHFQKNIANSPASVLAAFDSQLPGTQSLVASLFGALPDDILAKSFQLKHKQV<br>KKIKLRYAPKK
AtGLP5-3	AT5G38910.1	MKSFSFLAVLSILAITLSLSKASDPSSLQDFCVGVNTPADGVFVNGKFCKDPKLVTVEDFFFTGLHEAR<br>PPNPKTGSNVTAVNVNNLPGLNTLGISLVRIDYGVYGQNPPHTHPRASEVLYVAVGTLFVGFVTSNPE<br>NRLFSKTLYEGDVFVFPQGLIHFQVNVGKYPAVAFAGLSSQNPGVITIADTVFGSNPQIDPSFLASAFQ<br>VDPKIVMDLQTKFIKP
AtGLP5-4	AT5G38930.1	MAMKSLSFLAALSLLALTLPLTIASDPSQLQDFCVSANSSANGVFVNGKFCKDPKLVTADDFFFPGLQ<br>TARPITSPVGSTVTAVNVNNLLGLNTLGISLVRIDYAVDGQNPPHTHPRATEILVVELGTLLVGFVTSNP<br>DNRLFTKVLNEGDVFVFPEGLIHFQANIGKAPAVAFAALSSQNPGVITIAPTVFGANPAINPNILAKAFQ<br>VDPRVVMDLQTKFKK
AtGLP5-5	AT5G38940.1	MAMKSLSFLAVLSLLALTLPLAIASDPSQLQDFCVSANTSANGVFVNGKFCKDPKLVTADDFFFSGLQ<br>TARPITSPVGSTVTAVNVNNLLGLNTLGISLVRIDYAVNGQNPPHTHPRATEILVVEQGTLLVGFVTSNP<br>DNRLFSKVLNEGDVFVFPEGLIHFQANIGKAPAVAFAALSSQNPGVITIANTVFGANPAINPTILAKAFQ<br>LNPRVVMDLQTKFKK
AtGLP5-6	AT5G38960.1	MKNLYLAILYLLAASTLPFAIASDPSPLQDFCIGVNTPANALFVNGKFCKDPKLVTADDFYFSGLDKA<br>RTTESSPVGSNVTTVNVNQIPGLNTLGISLVRIDYGINGQNPPHTHPRATEILLVQEGTLFVGFFSSFPE<br>NRLFNKTLNKGDVFVFPEGLIHFQVNIGKQPAVAFASLSSQNPGVIIIGNTLFGSKPPIDPNVLAKAFQL<br>DPKVIIQLQKKFG
AtGLP5-7	AT5G39100.1	MGISLVRIDYAPYGQNPPHTHPRATEILVLIEGTLYVGFVSSNQDNNRLFAKVLYPGDVFVFPIGMIHF<br>QVNIGKTPAVAFAGLSSQNAGVITIADTVFGSTPPINPDILAQAFQLDVNIVEDLEAKFRN*
AtGLP5-8	AT5G39110.1	MRFSKSLILITLSALVISFAEANDPSPLQDFCVAIGDLKNGVFVNGKFCKDPKQAKAEDFFYSGLNQA<br>GTTNNKVKSNVTTVNVDQIPGLNTLGISLVRIDYAPYGQNPPHTHPRATEILVLVEGTLYVGFVSSNQ<br>DNNRLFAKVLNPGDVFVFPIGMIHFQVNIGKTPAVAFAGLSSQNAGVITIADTVFGSTPPINPDILAQAF<br>QLDVNVVKDLEAKFKN
AtGLP5-9	AT5G39120.1	MKVSMSLILITFWALVTIAKAYDPSPLQDFCVAIDDPKNGVFVNGKFCKDPKQAKAEDFFSSGLNQA<br>GITNNKVKSNVTTVNVDQIPGLNTLGISLVRIDYAPYGQNPPHTHPRATEILVLVEGTLYVGFVSSNQD<br>NNRLFAKVLNPGDVFVFPIGMIHFQVNIGKTPAVAFAGLSSQNAGVITIADTVFGSTPPINPDILAQAFQ<br>LDVNVVKDLEAKFKN
AtGLP5-10	AT5G39130.1	MRVSQSLIPFAIIALVLSFVNAYDPSPLQDFCVAIDDLKGVFVNGRFCKDPERVDAKDFFFSGLNVPGN<br>TNNQVGSNVTTVNVDQIPGLNTMGISLVRIDYAPHGQNPPHTHPRGSEILVLVEGTLYVGFVSSNQDN<br>NRLFAKVLHPGDVFVFPIGMIHFQLNIGKIPAIAFAGLSSQNAGVITIANTVFGSNPPIYPELLARAFQL

			DANVVKELQAKFGSI
	AtGLP5-11	AT5G39150.1	MKVSMSLILITLSALVTIAKAYDPSPLQDFCVAIDDPKNGVFVNGKFCKDPKQAKAEDFFSSGLNQAGITNNKVQSNVTTVNVDQIPGLNTLGISLVRIDYAPYGQNPPHTHPRATEILVLVEGTLYVGFVSSNQDNNRLFAKVLNPGDVFVFPIGMIHFQVNIGKTPAVAFAGLSSQNAGVITIADTVFGSTPPINPDILAQAFQLDVNVVKDLEAKFKN
	AtGLP5-12	AT5G39160.1	MRVSQSLVPFAIIALVLSFVNAYDPSPLQDFCVAIDDLKGVFVNGRFCKDPKRVDAKDFFFSGLNMPGNTNNQVGSNVTTVNVDQIPGLNTMGISLVRIDYAPHGQNPPHTHPRGSEILVLVEGTLYVGFVSSNQDNNRLFAKVLHPGDVFVFPIGMIHFQVNVGKIPAVAFAGLSSQNAGVITIANTVFGSNPPIYPELLARAFQLDASVVKELQAKFGSI
	AtGLP5-13	AT5G39180.1	MKVSMSLILITLSALVTIAKAYDPSPLQDFCVAIDDPKNGVFVNGKFCKDPKQAKAEDFFSSGLNQAGITNNKVQSNVTTVNVDQIPGLNTLGISLVRIDYAPYGQNPPHTHPRATEILVLVEGTLYVGFVSSNQDNNRLFAKVLNPGDVFVFPIGMIHFQVNIGKTPAVAFAGLSSQNAGVITIADIVFGSTPPINPDILAQAFQLDVNVVKDLEAKFKN
	AtGLP5-14	AT5G39190.1	MRVSQSLVPFAIIALVLSFVNAYDPSPLQDFCVAIDDLKGVFVNGRFCKDPKRVDAKDFFFSGLNVPGNTNNQVGSNVTTVNVDQIPGLNTMGISLVRIDYAPHGQNPPHTHPRGSEILVLVEGTLYVGFVSSNQDNNRLFAKVLHPGDVFVFPIGMIHFQVNVGKIPAVAFAGLSSQNAGVITIANTVFGSNPPIYPELLARAFQLDASVVKELQAKFGSI
	AtGLP5-15	AT5G61750.1	MKFFVVIVFCAIFLSVSGDSDNMQDTCPTAPGEQSIFFINGYPCKNPTKITAQDFKSTKLTEAGDTDNYLQSNVTLLTALEFPGLNTLGLSVSRTDLERDGSVPFHSHPRSSEMLFVVKGVVFAGFVDTNNKIFQTVLQKGDVFVFPKGLLHFCLSGGFEPATAFSFYNSQNPGVVNIGEVFGIDQEHIKIMTRCLATGSGCRVTDGDEL
Oryza sativa	OsGLP1-1	LOC_Os01g14670.1	MAGAAGSSWSSLLLAAVAVALAVAAAPSLAGDPDYLQDICVADLNSEVKVNGFPCKANATADDFFSGVLASPGAAANTTTGAVVTGANVEKVPGLNTLGVSLARIDYAPGGLNPPHTHPRATEVVFVLYGELDVGFVTTANKLLSRTISQGDVFVFPRGLVHFQRNTGDKPAAVVSAFNSQLPGTQSIAATLFAASPAVPDAVLAKAFQIDDEEVDKIKAKFAPKKT
	OsGLP1-2	LOC_Os01g18170.1	MARVQLWVAAACAVVLALAAPSLAGDPDMLQDVCVADLASPVKLNGFPCKANVTADDFFFAGLKNPGNTNNPAGSNVTAANVQSFPGVNTLGVSMARIDYAPGGQNPPHTHPRATEIIFVLEGVLEVGFITTANKLFTKTVTAGEVFVFPRGLVHFQQNRGHGPAAVIAAFNSQLQGTQAIAATLFAAAPPVPSDVLAKAFRVDVPQVDAIKAKFK
	OsGLP1-3	LOC_Os01g50900.1	MASSRSVVLRVLVAVAVVAAAGAPRLAVADSPPLQDICVADLRAATAVDGFPCKPTASVVSDDFFCDAIVQAPSTSNPFGVNSTRATVSAFPGLNTLGLSITRTDLAPGGLNPPHSHPRASELVLVLSGEVMVGFTTAANRLFSKVVREKELFVVPRGLQHFQLNVGAGNASFVAMFDSQSPGLVTPTFALFATQPAMPMEVLA

		KTFLMGEDEVGAIKSKFAGF
OsGLP1-4	LOC_Os01g72290.1	MAKLILATFAVVFMALAATSLAGDPDMLQDVCVADYKSLKGPLRLNGFPCKRIENVTANDFFFDGLMKAGNTGNAVGSVVTAASVESLPGLNTMGVSMARIDYAPWGLNPPHTHPRATEIIFVVEGSLDVGFVTTANKLFTRTVCKGEVFVFPRGLVHFQKNNGNTPAFAIAALNSQLPGTQSIAAALFGAAPPLPSDTLARAFQVDGGMVEFIKSKFVPPKY
OsGLP1-5	LOC_Os01g72300.1	MAAKLPTVVLLASFAAVILSLAAPLLAGDPDMLQDICVADYKSLQGPLRVNGFPCKPEANVTAEDFFFPGLGKPADVYSGNPMGSAVTAATVERIPGLNTLGVSMARVDYAPWGGANPPHSHPRATEILFVADGLLEVGFVVATAAPASSRLITRVVPKGGVFVFPRGLLHYERSVGEKPAVAISAFDSQLPGTQAAADALFGSSSPAVPTDVLARAFQVDGGVVENIKSKFQHK
OsGLP2-1	LOC_Os02g29000.1	MASTWFFLLALLAVSISNAFASDPSQLQDFCVADKMSQVLVNGFACKDPAAITVEDFFFSGLHMAGNTSNRQGSAVTGVNVAQISGLNTLGISLARVDYAPYGLNPPHIHPRATEILTILEGSLYVGFVTSNPENKLFTKVLNKGDVFVFPQGLIHFQFNYGTKDVIALAALSSQNPGVITIANAVFGSKPFISDDILAKAFQVEKKIVDRIQAQF
OsGLP2-2	LOC_Os02g29010.1	MAAVGACFLQQLAVVALLALWCSHGAIASDPGLLQDFCVVDKMSQVRVNGFPCKDAKDVVAGDFFFSGLHMAGNTTNKQGSNVTTVNVAQIPGLNTMGVSLVRIDYAPNGLNPPHTHPRATEIPTVLEGSLYVGFVISNPENKLFTKVLNKGDVFVFPQGLVHFQFNNGTNNAVALAALSSQNPGVITVGNAVFGSKPSISDDILAKAFQEMFKFECDSSERNFGYVAPSPLRLLTITLSLPYMSFTLSSVLISYLKIANSIKILLVQGVEQGIYGLGIELEIAMVNILQVVAGDSKTYINNISNVEAHSLKPLVYQSDRSQRGHTGVGSISSGSPNGGHHTGSLAARPIECGGLGIMNLEFFGQALRLKWLAQRLEQKGRPWTLVNSRPTTDKQDLFRAASTLEGLENADRIVGGAVPTARRRDRRAIKVVATSPARLWLQGGLRLVVHACLLGGLERKECQDFRAQKLHGGVGLQGYPGGSPRLERSRRVQRMRVKLRESFFVLFWGLWFPADLPTSTV
OsGLP2-3	LOC_Os02g29020.1	MAAIRASFLLAAAALLALWCSDHGGVVASDPSHLQDLCVADKASTVRVNGVACKDGEDVAAEDFFFSGLHMAGNTTNKQGSAVTAVNVAQVPGLNTLGISLARIDYALHGLNPPHTHPRATEILTVLEGSLYVGFVTSNPENKLFTKVINKGDVFVFPKGLVHFQFNYGTTDAVAIVALSSQNPGVITVANAVFGSKPSITDDILAKAFQVEKTVVDQIQAKF
OsGLP2-4	LOC_Os02g32980.1	MAHRRRCLLLLLAVLLPAMAARGDPDAVQDFCVPDAGRGRPVELAMLPAYPCRSPANLTAGDFAFSGVRAAGNFSPETGFAGVSVTPAQFPGLHTLGMSFARADLSAAGGVNPPHYHPRATETALVLAGRVYAGFVDSGGRLFAKVLEQGEVMVFPRAMVHFQLNVGDTPATVYGAFNSENPGIVRIPATVFGSGIREAVLERAFGLTPAELRRLEKRFGPPKKAEMED
OsGLP3-1	LOC_Os03g08150.1	MRAAVAHRILLSLALFAVLCRCDPDLLFDYCVADTAAATAAGAFHLNGLACIDPALARADHFATSALSRATNPAATLYGFNATLTSPAASLPGANAQGLAMARIDLAPGGMAPPHSHPRASEAALVLSGSVLVGFADTSYRLYTQLLRAGEAFVFPRAMVHFLYNMDTAAPAVVLSGLNSQSPGAQLVPFSAFRTEPRLPDEVL

		KKAFKITGQDVQRIQKHLGGL
OsGLP3-2	LOC_Os03g44880.1	MAKLILATFAVVFLALAATSLAGDPDMLQDVCVADYKSLRGPLRLNGIPCKRLENVTANDFFFDGLTNAGNTTNAVGSLVTAASVERLPGLNTMGVSMARIDYAPWGLSPPHTHPRATEIMFVAEGTLDVGFVTTANKLFTRTVSKGEVFVFPRGLVHFQRNSGNTSALAIAAFNSQLPGTQSIADTLFGAAPPLPSDTLARAFQVDGGMVESIKSKFPPKY
OsGLP3-3	LOC_Os03g48750.1	MECFKTTLAGVVLVVLLLQQAPVLRANDPDPLQDFCVADLDSEVTLNGYPCKPTPAAGDEFLFSSRLATGGDVNANPNGSNVTQLDVAGWPGVNTLGVSMNRIDFAPGGTNPPHVHPRATEVGIVLRGELLVGIIGSLDTGNRYYSRVVRGGETFVIPRGLMHFQFNVGKTEATMVVSFNSQNPGIVFVPLTLFGSNPPIPTPVLVKALRVDAGVVELLKSKFTGGY
OsGLP3-4	LOC_Os03g48760.1	MEHSFKTITAGVVFVVLLLQQAPVLIRATDADPLQDFCVADLDSKVTVNGHACKPASAAGDEFLFSSKIATGGDVNANPNGSNVTELDVAEWPGVNTLGVSMNRVDFAPGGTNPPHVHPRATEVGIVLRGELLVGIIGTLDMGNRYYSKVVRAGETFVIPRGLMHFQFNVGKTEATMVVSFNSQNPGIVFVPLTLFGSNPPIPTPVLVKALRVDTGVVELLKSKFTGGY
OsGLP3-5	LOC_Os03g48770.1	MEYGFKAAGLVFVVLLLQQAPVLIRATDADPLQDFCVADLNSEVTVNGHACKPASAAGDEFLFSSKIATGGDVNANPNGSNVTELDVAEWPGVNTLGVSMNRVDFAPGGTNPPHVHPRATEVGIVLRGELLVGIIGTLDTGNRYYSKVVRAGETFVIPRGLMHFQFNVGKTEATMVVSFNSQNPGIVFVPLTLFGSNPPIPTPVLVKALRVDAGVVELLKSKFTGGY
OsGLP3-6	LOC_Os03g48780.1	MEHSFKTIAAGVVIVVLLLQQAPVLIRATDADPLQDFCVADLDSKVTVNGHACKPASAAGDEFLFSSKIATGGDVNANPNGSNVTELDVAEWPGVNTLGVSMNRVDFAPGGTNPPHVHPRATEVGIVLRGELLVGIIGTLDTGNRYYSKVVRAGETFVIPRGLMHFQFNVGKTEATMVVSFNSQNPGIVFVPLTLFGSNPPIPTPVLVKALRVDAGVVELLKSKFTGGY
OsGLP3-7	LOC_Os03g58980.1	MSSSSSMECTGNMSAAPLLVLTVAVLAVLASTCAADPEPIQDFCVAVPRAGGEASPAYPGFPCKPASAVVSDDFFFAGLAAAGSTDNPFGASLKPGNVEAFPALNTLGVAINRVDLAPGGVNPLHSHPRAAELVHVITGRMLVGFVSTAGKYYSKVVGEGETFAIPRGLMHFQYNPGNASARAMTVFNSQLPGVVPAATALFGADPEIPDAVLAKSFQVDAEIIKLLKSKFKK
OsGLP3-8	LOC_Os03g59010.1	MSRTSSAPLLVLSAALAVLASTCIADPEPVQDFCVAVVPRAGDAAAAACPAYPGFPCKPASTVVSDDFFFAGLAVASDTDNRFGFNVTAANAETFPGLNTLGVSIGRVDLAPGGVNPLHSHPRATELIHVVAGRVLAGFVSTAGEFYSKVLGEGETFVVPRGMIHFQYNVGGVAAQVITAFNSQMPGVVAAGSTLFGSDPEIPDAVLAKSFQVDAKIIKLLKSKF
OsGLP3-9	LOC_Os03g58990.1	MECTEAMSRTSSSAPLLVLTPDGESTAVISDDFFFAGNTENRFGFNATLGNVQAFPGLNTLGVSINRGDFAPGGLNALHSHPRAAELVHVSSPYNVGDVAAQVITAFNSQLPGVVAAAPSLFGSDPEIPDAVLAENYQVDVKIIRLLKSKF

OsGLP4-1	LOC_Os04g52720.1	MASRAFAAVFAAVALVVCSSVLPRALASDPSQLQDFCVADKLSAVFVNGFVCKNPKQVTANDFFLPKALGVPGNTVNAQGSAVTPVTVNELPGLNTLGISFARIDFAPNGQNPPHTHPRATEILTVLQGTLLVGFVTSNQPGGGNLQFTKLLGPGDVFVFPQGLIHFQLNNGAVPAVAIAALSSQNPGVITIANAVFGSTPPILDDVLAKAFMIDKDQVDWIQAKFAAPPAASGGGGGFIGGGGGGGFPGGGAP
OsGLP5-1	LOC_Os05g10830.1	MAMVGRSLLLLLLLVTLAAGHGVVVVVAFDPNPLQDFCVADPTSKVRVNGLPCKDPAAVTADDFFFSGVGEPAAGGGRGATASRRYGFTARSVDIPGLNTLGASAARVDVAPGGVFPPHYHPRASETAVVLAGAVYFGFVTSYPDSRVVAKVLRRGDVFAVPQGLVHFLHNNGSEPAALYASLSSQNPGLVLVADALLAAPLPVDLVAKTLLTDEATVDKIRANFIVHRS
OsGLP5-2	LOC_Os05g19670.1	MARPSLPCAVVAVLLLALLPTPSTAGDPDLLQDICVADLTSAVKVNGFACKAAVTEDDFYFKGLAAAGNTNNTYGSVVTGANVEKLPGLNTLGVSMSRIDYAPGGLNPPHTHPRATEMVFVLQGTLDVGFITTANKLYTKTISAGDVFVFPRGLLHFQKNNGDTPAAVISAFNSQLPGTQSLAMTLFAASPEVPDGVLTKAFQVGTKEVEKIKSRLAPKKR
OsGLP8-1	LOC_Os08g08920.1	MASFISFLLLAALIGMASWQAIAAEPSPLQDFCVADLNSAVRVNGFACKNPTNVSADDFFKAAMLDKPRDTAVNKVGSNITLINVMEIPGLNTLGISIVRVDYAPLGLNPPHTHPRATEIFTVLEGTLYVGFVTSNPDNKLFSKVLNKGDVFVFPKGLIHFQFNLDPHKPAIATSAISSQNPGIITIANAVFRSNPPISDDILAKAFQVDKKIIDLLQA
OsGLP8-2	LOC_Os08g08960.1	MASSSFSFLLVAALLGLASWKAIASDPSPLQDFCVADLNSPVRVNGFVCKNPMNASADDFFKAAMLDKPRDTNNKVGSNVTLVNVLQLPGLNTLGISIARLDFAPLGLNPPHTHPRATEIFTVLEGTLYVGFVTSNPDNRLLSKVLNKGDVFVFPEGLIHFQFNPNPHKPAVAIAALSSQNPGVITIANAVFGSNPPISDDILMKAFQVDKKIIDLLQAQF
OsGLP8-3	LOC_Os08g08970.1	MASSSLFLLASLLVLASWQQAIAFDPSPLQDFCVADMASPVRVNGFPCKNPMNVTSDDFFNAAKFDMPRNTMNKVGSNVTNLNVINFPGLNTLGISLARIDYAPMGVNPPHVHPRATELLTVLEGTLYVGFVTSNPNRLFSKVVHKGDVFVFPKAMIHFQMNLDHNKPAVAQSALSSQNPGVITIASAIFGSTPPISDDVLVKAFQVEKKVIDWLKSQFSENNHY
OsGLP8-4	LOC_Os08g08980.1	MASSSSLYLLAALLALASWQAIAFDPSPLQDFCVADMKSPVRVNGFPCKNPMEVNSDDFFNAAKFDMPRSTMNKVGSNVTNLNVLNFPGLNTLGISLARIDYAPLGVNPPHIHPRATELLTVLEGTLYVGFVTSNPNRLFSKVVHKGDTFVFPKAMIHFQMNLDHNKPAVAQSSLNSQNPGVITIASAVFGSKPPISDDVLTKAFQVEKKVIDWLKSQFWESNY
OsGLP8-5	LOC_Os08g08990.1	MASPSSLCLLAALALISWQAMASDPSPLQDFCVADMHSPVRVNGFACLNPMEVNADHFFKAAKLDTPRKTNKVGSNVTLINVMQIPGLNTLGISIARIDYAPLGQNPPHTHPRATEILTVLEGTLYVGFVTSNPNNTLFSKVLNKGDVFVFPQGLIHFQFNPNPHQPAVAIAALSSQNPGAITIANAVFGSKPPISDEVLAKAFQVEKGTIDWLQAQFWENNHY

OsGLP8-6	LOC_Os08g09000.1	MASPSSLCLLTALLALVSWQTIASDPSPLQDFCVADEHSPVLVNGFACLDPKHVNADHFFKAAMLDTPRKTNKVGSNVTLINVMQIPGLNTLGISIARIDYAPLGQNPPHTHPRATEILTVLEGTLYVGFVTSNPNNTLFSKVLKKGDVFVFPVGLIHFQFNPNPHQPAVAIAALSSQNPGAITIANAVFGSKPPISDEVLAKAFQVEKGTIDWLQAQFWENNHY
OsGLP8-7	LOC_Os08g09010.1	MASPSSFCLLAVLLALVSWQAIASDPSPLQDFCVADKHSPVLVNGFACLDPKYVNADHFFKAAMLDTPRKTNKVGSNVTLINVMQIPGLNTLGISIARIDYAPLGENPPHTHPRATEILTVLEGTLYVGFVTSNPNNTLFSKVLNKGDVFVFPEGLIHFQFNPNPHQPAVALAALSSQNPGAITIANAVFGSKPPISDDILAKAFQVEKGTIDWLQAQFWENNHY
OsGLP8-8	LOC_Os08g09020.1	MASPSFCLLAALLALVSWQAIASDPSPLQDFCVADKHSPVLVNGFACLDPKYVTADHFFKAAMLDTPRKTNKVGSNVTLINVMQIPGLNTLGISIARIDYAPLGENPPHTHPRATEILTVLEGTLYVGFVTSNPNNTLFSKVLNKGDVFVFPEGLIHFQFNPNPHQPAVAIAALSSQNPGAITIANAVFGSKPPISDKVLAKAFQVEKGTIDWLQAQFWENNHY
OsGLP8-9	LOC_Os08g09040.1	MASPSFCLFAALLALVSWQAIASDPSPLQDFCVADKHSPVLVNGFACLDPKYVTADHFFKAAMLDTPRKTNKVGSNVTLINVMQIPGLNTLGISIARIDYAPLGQNPPHTHPRATEILTVLEGTLHVGFVTSNPNNTLFSKVLNKGDVFVFPVGLIHFQFNPNPHQPAVAIAALSSQNPGVITIANAVFGSKPPISDEVLAKAFQVGKGTIDWLQAQFWENNHY
OsGLP8-10	LOC_Os08g09060.1	MASPSICLLAALLALVSWQAIASDPSPLQDFCVADMHSPVLVNGFACLDPKYVNADHFFKAAMLDTPRKTNKVGSNVTLINVMQIPGLNTLGISIARIDYAPLGENPPHTHPRATEILTVLEGTLYVGFVTSNPNNTLFSKVLNKGDVFVFPEGLIHFQFNPNPHQPAVAIAALSSQNPGAITIANAVFGSKPPISDIVLAKAFQVEKGTIDWLQAQFWENNHY
OsGLP8-11	LOC_Os08g09080.1	MASSSFLLLATLLAMASWQGMASDPSPLQDFCVADMHSPAVLVNGFACLNPKDVNADHFFKAAMLDTPRKTNKVGSNVTLINVMQIPGLNTLGISIARIDYAPLGQNPPHTHPRATEILTVLEGTLYVGFVTSNPDNKFFSKVLNKGDVFVFPVGLIHFQFNPNPYKPAVAIAALSSQNPGAITIANAVFGSKPPISDDVLAKAFQVEKGTIDWLQAQFWENNHY
OsGLP8-12	LOC_Os08g13440.1	MASSSLFLLGALLVLASWQAIVAYDPSPLQDFCVADMNSPGVLRVNGFACKNPMDVSSEDFFNAAKFDMPRNTFNKLGSNVTNLNVMEFPGLNTLGISLARIDYAPMGVNPPHIHPRATELLTVLEGTLYVGFVTSNPNKLFSKVVCKGDVFVFPKAMIHFQMNLDHDKPAVAQSALSSQNPGVITIASAVFGSQPPISDDVLTKAFQVEKKLIDWLQSQFWENNY
OsGLP8-13	LOC_Os08g35750.1	MSSTPLLPVLLSTMILLSAVSTTTTALTQDFCVANLPLGADTPSGYQCRPAATVTAADFYSGALARPGILIRPFNTSLASAFVQQYPAVNGLGISASRVDILPGGVVPLHTHPAGSELLYVLDGALVAGFISSSDNKVYYKEVSKGGMFVFPQGLLHFQYNTGDTTAVAFAAYSSSNPGLQILDYALFANNLPTSYVVKGTFLAEAEVRRLKSKFGGSG

OsGLP8-14	LOC_Os08g35760.1	MAKAVMMLPVLLSFLLLPFSSMALTQDFCVADLTCSDTPAGYPCKASVGAGDFAYHGLAAAGNTSNLIKAAVTPAFVGQFPGVNGLGISAARLDIAVGGVVPLHTHPAASELLFVTQGTVAAGFITSSSNTVYTRTLYAGDIMVFPQGLLHYQYNAGQSAAVALVAFSGPNPGLQITDYALFANNLPSAIVEKVTFLDDAQVKKLKSVLGGSG
OsGLP9-1	LOC_Os09g39510.1	MMMSSRSSVSLGVLLLLAVILSAGAADPDILTDFVVPSDTDPSGIDGAFFTYKNLVTGNSGDPAKLTVTKATHAEFPALLGQSVSYAALVFGAGTVNPPHIHPRASELLVVVQGPLLVGLVDAARNGTVYTQTLQTGDMFVFPKGMVHFQFNNGTDVVARAFSAFGSATPGTISLPAALFGSGIDDTILDKSMHTDQATVDQLKQDQAPPSPRPSPGSSSSAAAALLPSRWAITLLLCFAASYYFYF
OsGLP9-2	LOC_Os09g39520.1	MALSYYSLLLLLLAVWAPALTLVMAGDPDILTDYVIPANGNPMNITGDFFTFTGFRKVFNTSSAPEPNSFTVTKATMAEFPALNGQSVSYATLVFPPSTVNPPHTHPRSAELLLVVDGALSVGFIDTTNKLYTQDLAAGDMFVFPKGMVHFQFNSGNQPAMALSAFGSAAPGVVPVPVTVFGTGIDDAVLAKSFKTDVPTILKLKANLTPPNKS
OsGLP9-3	LOC_Os09g39530.1	MASSILLLVVLAVVSAPVALVMAGDPDILTDYVIPAGSNAENITGDFFTFTGFRNPLSMNMSMPMPNANFTVTKATMAEFPALNGQSVSYAVLMYPPATLNPPHTHPRSAELLLLVDGALSVGFVDTTNKLYTQDLAAGDMFVFPKGMVHFQFNSGNQPAMALSAFGSAAAGLVSVPVTVFGTNIDDAVLAKSFKTDVPTIQKLKAGLTPPKKA
OsGLP11-1	LOC_Os11g33110.1	MKLSTVLCCYLLLLGLFAPEIISDSPPLQDVCPMAPQGERKLFMNGFFCKSPSTIMASDFKTLLLNHAGDLDNMVRSSANIITATEFPGLNTLGISMARTDIAVSGAVLPHSHPRASEMMFVHSGSVVAGFFDTKGKLFQKTLAEGDVFIFPRGLVHFIMNYGFGLATTFSVLNSQNPGVVGITHAMFAPDSEVAEGLMARMLSFRDMGMDDSSSVDSPWFY
OsGLP12-1	LOC_Os12g05840.1	MASSNFFLPTALIALVATQAMAFDPSPLQDFCVADRNSPVRVNGFPCKDAKDVNVDDFFLEANLDKPMDTTKSKAGSNVTLINVMKLTGLNTLGISMARIDYAPKGQNPPHTHPRATEILTVFEGTLYVGFVTSNQANGENKLFTKTLNKGDVFVFPQGLIHFQFNPSYDKPAVAIAALSSQNPGAITIANAVFGSNPPISDDVLAKAFQVDKKAVDWLQAQFWENNHN
OsGLP12-2	LOC_Os12g05860.1	MASSNFFLLTALIALVATQAMASDPSPLQDFCVADRNSPVHVNGFPCKDAKDVNVDDFFLAANLDKPMDTTKSKAGSNVTLINVMKLAGLNTLGISMARIDYAPKGQNPPHTHPRATEILTVLEGTLYVGFVTSNQANGENKLFTKTLNKGDVFVFPQGLIHFQFNPSYDKPAVAIAALSSQNPGAITIANAVFGSNSPISDDVLAKAFQVDKKAVDWLQAQFWENNHN
OsGLP12-3	LOC_Os12g05870.1	CKDAKDVSVDDFFLAANLDKPMDITKSKAGSNVTLINVMKLAGLNTLGISMARIDYAPKGQNPPHTHPRATEILSVIEGSLYVGFVTSNQANGENKLFTKTLNKGDVFVFPEGLIHFQFNPSYDKPAAAIVALSSQNPGAITIANAVFGSNPPISDDVLAKAFQVDKKAVDWLQAQFWENNHN
OsGLP12-	LOC_Os12g0	MFFNAVRVNGLPCKDAKDVSVDDFFLAANLDKPMDTTKSKAGSNVTLINVMKLAGLNTLSISMARI

	4	5880.1	DYAPKGQNPPHTHPRATEILTVLEGSLYVGFVTSNQANRENKLFTKTLNKGDVFVFPQGLIHFQFNPSYDKPAVAIAALSSQNPGAITIANAVFGSHPPISDDVLAKAFQVDKKAMDWLQAQFWENNHN
Puccinellia tenuiflora	PutGLP1	Pt_Chr0205171	MASSSYFLLVVLFALASWQAIASDPSPLQDFCVADNSSHVLVNGFVCKDPKDVKAEDFFLAAKLDMPRDTKMSKVGSNVTLINVMRIPDLNTLGISLARIDYAPLGENPPHTHPRATEIITVLEGTLYVGFVTSNPNNTFLSKVLNEGDVFVFPEGLIHFQFNPNPYKPAVAIAALSSQNPGAITIANAVFGSKPAISDDVLAKAFQVDKNTVDWLQAQFWADNHN
	PutGLP2	Pt_Chr0205214	MDPKDVKAEDFFLAAKLDMPRDTKMSKVGSNVTLINVMRIPGLNTLGISLARIDYAPLGENPPHTHPRATEILTVLEGTLYVGFVTSNPNNTFLSKVLNEGDVFVFPEGLIHFQFNPNPYKPAVAIAALSSQNPGAITIANAVFGSKPAISDDVLAKAFQVDKNTVDWLQAQFWADNHN
	PutGLP3	Pt_Chr0205170	MASSSYLLLIALLALASWQAIASDPSPLQDFCVADNSSHVLVNGFVCKDPKDVKAEDFFLAAKLDMPRDTKMSKVGSNVTLINAMRLPGLNTLGISLARIDYAPLGENPPHTHPRATEILTVLEGTLYVGFVTSNPNNTFLSKVLNKGDVFVFPEGLIHFQFNPNPYKPAVAIAALSSQNPGAITIANAVFGSKPAISDDVLAEAFQVDKKTVDRLQAQFWADNHN
	PutGLP4	Pt_Chr0402444	MWSAYVLINVMNWIASNMNKMADHLNSTINQMATSYILLAALLALVSWQAMASDPSPLQDFCVADKNSPVLVNGLVCKDPKVIPILALSKTSVLQTLAHMLMDSSSRTPNVVTAEDFFLADKRDMPRDTKISKIGSNVTLINVMKILGLNTLGISMAHIDYAPLGENPPHIHPRATEILTVLEGTLYVVFVTSNTKNKIFSKDLKKDDVFLFPQELIHFQFNPNSYKPAVAINPGAITIANNVFGSKPMISDNLLAKAFQVEKKTVDWLQAHSRPTTTII
	PutGLP5	Pt_Chr0207227	MASFSSSCFFLIAATLAVISWQAVASDPSPLQDFCVADNSSRVLANGFVCKDPKVVTAEDFFLAAKLDMPRDTKMSKVGSNVTLINVMKIPGLNTLGISLARIDYAPLGQNPPHTHPRATEILTVLEGTLYVGFVTSNPENKLFSKELNKGDVFVFPQGLIHFQFNPNPYKPAVAIAALSSQNPGAITIANAVFGSKPMISDDVLAKAFQVQKKTVDWLQAQFWADNHN
	PutGLP6	Pt_Chr0205169	MASFSCSCFLLFAAILAVISWQPVASDPGPLQDFCVADNSSRVLVNGFVCKDPKVVTAEDFFLAAKLDMPRDTKMSKVGSNVTLINVMKIAGLNTLGISLARIDYAPLGENPPHTHPRATEILTVLEGTLYVGFVTSNTDNRLFTKELNKGDVFVFPQGLIHFQFNPNPYKPAVALAALSSQNPGAITIANAVFGSQPMISDDVLAKAFQVEKKTVDWLQAQFWEDNHN
	PutGLP7	Pt_Chr0205212	MASSSSCFLLFAAILAVISWQAVASDPGPLQDFCVADNSSRVLVNGFVCKDPKVVTAEDFFLAAKLDMPRDTKMSKVGSNVTLINVMKIAGLNTLGISLARIDYAPLGENPPHTHPRATEILTVLEGTLYVGFVTSNTDNRLFTKELNKGDVFVFPQGLIHFQFNPNPYKPAVALAALSSQNPGAITIANAVFGSQPMISDDVLAKAFQVEKKTVDWLQAQFWEDNHN
	PutGLP8	Pt_Chr0402443	MAGHSLDPSPLQDFCVVDKNSPVFVNGFVCKNPMDVKADDFFKAANLDKPRMTNKVGSNVTLVNVMEIAGLNTLGISLARIDYAPLGQNPPHTHPRATEILTVLEGTLYVGFVTSNPENRFLSKVLNKGDVFV

		FPVGLIHFQFNPNPHKPAVAIAALSSQNPGAITIANAVFGSKPPISDDVLAKAFQVEKKTVDWLQAQFWENNHY
PutGLP9	Pt_Chr0402446	MASSSSFLVLAALLALGSWQAIASDPSPLQDFCVVDKNSPVFVNGFVCKNPMDVKADDFFKAANLDKPRMTNKVGSNVTLVNVMEIAGLNTLGISLARIDYAPLGQNPPHTHPRATEILTVLEGTLYVGFVTSNPENRFLSKVLNKGDVFVFPVGLIHFQFNPNPHKPAVAIAALSSQNPGAITIANAVFGSKPPISDDVLAKAFQVEKKTVDWLQAQFWENNHY
PutGLP10	Pt_Chr0402451	MASSSSFLVIAVLALISWQAIASDPSPLQDFCVVDKKSLVFINGFVCKNPIDVNADDFFMAAKLDKPRMTNKVGSNVTLINVMQIPGLNTLGISLARIDYAPLGQNPPHTHPRATEILTVLEGTLYVGFVTSNPENRFLSKVLNKGDVFVFPEGLIHFQFNPNPYKPAVAIAALSSQNPGAITIANAVFGSKPPISDDVLAKAFQVEKKTIDWLQAQFWENNHY
PutGLP11	Pt_Chr0402445	MASSSSFLVIAVLALISWQAIASDPSPLQDFCVVDKKSPVFINGFVCKNPIDVNADDFFMAAKLDKPRMTNKVGSNVTLINVMQIPGLNTLGISLARIDYAPLGQNPPHTHPRATEILTVLEGTLYVGFVTSNPENRFLSKVLNKGDVFVFPEGLIHFQFNPTRTSQQLQLLHSVARTLELSPLPMRCLDQSHQSQMMFLPRHSKWRRRR
PutGLP12	Pt_Chr0402447	MASSSSFLVIAVLALISWQAIASDPSPLQDFCVVDKKSPVFINGFVCKNPIDVNADDFFMAAKLDKPRMTNKVGSNVTLINVMQIPGLNTLGISLARIDYAPLGQNPPHTHPRATEILTVLEGTLYVGFVTSNPENRFLSKVLNKGDVFVFPEGLIHFQFNPNPYKPAVAIAALSSQNPGAITIANAVFGSKPPISDDVLAKAFQVEKKTIDWLQAQFWENNHY
PutGLP13	Pt_Chr0704422	MASSSSLFITIALLALLGSWQAAAYDPSPLQDFCVADKKSPVFVNGFPCKDPMAVNSDDFFNAAMLDQPRDTMKSKVGSNVTNVNVINFPGLNTLGISLARIDYGPLGVNTPHVHPRATELLTVLEGTLYVGFVTSNPNKLFSKVVKKGDVFVFPNAMIHFQMNLAHDKPAAALSSLNSQNPGVISIANAVFGSKPPISDDVLATAFQVEKGLIHWLQSQFWEDNNNY
PutGLP14	Pt_Chr0704423	MPQWTQSHNIFTMASSSSLFITIALLALLGSWQAAAYDPSTLQDFCVADEKSPVFVNGFPCKDPMAVNSDDFFNAAMLDQPRDTMKSKVGSNVTNVNVINFPGLNTLGISLARIDYGPLGVNTPHIHPRSTELLTVLEGTLYVGFVTSNPNKLFSKVVKKGDVFVFPNAMIHFQMNLAHDKPAAALSSLNSQNPGVISIANAVFGSKPPISDDVLATAFQVEKGLIHWLQSQFWEDNNNY
PutGLP15	Pt_Chr0704421	MASSSSMLVFIGLLALVSWQATAYDPSPLQDFRVADMKSSVFVNGFPCKDSMAVNSDDFFNPAMLDQPRDTMKSNVGSNVTNVNVVNFPGINTLGISLARIDYGPLGVNTPHVHPRATELFTVLEGTLYLGFVTSNPNRLFSKVVKKGDVFVFPKAMIHFQMNLAHDKLAAALSSLNSQNPGVISIANAVFGSKPSISDDVLARAFQVEKDLIHWLQSQFWEDNNND
PutGLP16	Pt_Chr0704687	MPPCQNQLTMASSSSLCILALLLVLASWQATAFDPSPLQDFCIADMKAPVQVNGFACKDPMNATPDDFFNAAKLDQARDTKGSKVRSNVTNVNVINFPGLNTLGISLARIDYGPLGVNTPHVHPRATELLTVLEG

		TLYLGFVTSNPNKLFSKIVKKGDVFVFPKAMIHFQMNLSPDKPAAALSSLNSQNPGVISIANAVFGSKPPISDDVLATAFQVEKKLIDWLQSQFWENNNY
PutGLP17	Pt_Chr0704688	MHIGTLLLEQPQLIRGYLSTSIHVFMDTKQLTMASPSLLFVLLGLALASWQTTAYDPSPLQDFCIADMKAPVRVNGFACKDPMAATPEDFFNPAMLDKARDTKGSKVGSNVTNINVINFPGLNTLGISLARIDYGPLGVNTPHIHPRATELLTVLEGTLYLGFVTSNPNRLFSKIVKKGDVFVFPKAMIHFQMNLAHDKPAAALSSLSSQNPGVISIANAVFGSKPPISDDVLATAFQVEKDLIHWLQSQFWENNNY
PutGLP18	Pt_Chr0704689	MKAPVHVNGFACKDPMEATPEDFFNPAMLDQARDTKGSRVRSNVTNINVINFPGLNTLGISLARIDYGPLGVNTPHIHPCATELLTVLEGTLYLGFVTSNPNRLFSKIVKKGDVFVFPKAMIHFQMNLAHDKPAAALSSLSSQNPGVISIANAVFGSNPPISDDVLATAFQVEKDLIDWLQSQFWENNNY
PutGLP19	Pt_Chr0704690	MASSSSLCLLVVLLALASWQTTAYDPSPLQDFCIADMNAPVRVNGFACKDPMAATPEDFFNPAMLDQARDTRGSRVGSNVTVVNVINFPGLNTLGISLARIDYGPLGVNPPHIHPRASELLTVLEGTLYLGFVTSNPNRLFSKIVKKGDVFVFPKAMIHFQMNLAHDKPAAALSSLGSQNPGVITIANAVFGSKPPISDDVLARAFQVEKDLIDWLQSQFWENNN
PutGLP20	Pt_Chr0704691	MPSWTKQLTMASSSSVCLLVVLLALASWQTTAYDPSPLQDFCIADMNAPVRVNGFACKDPMAATPEDFFNPAMLDQARDTRGSRVGSNVTVVNVINFPGLNTLGISLARIDYGPLGVNTPHTHPRATELLTVLEGTLYLGFVTSNPNRLFSKIVKKGDVFVFPKGMIHFQMNLAHDKPAAALSSLSSQNPGVISIANAVFGSNPPISDDVLATAFQVEKDLIDWLQSQFWENDN
PutGLP21	Pt_Chr0502264	MASKIILLALLTLSVSRAAASDPGQLQDFCVADRTSEVFVNGFFCKDPKTAMVDDFFFSGLHKAGNTSNKQGSDVTSVNVAQIAGLNTLGVSLARVDFAPYGQNPPHIHPRASEILTVLEGSLYVGFVTSNPENKLFTKILNKGDVFVFPQGLIHFQFNSGTNKAIAIAALSSQNPGVITIANAVFGSKPSISDDMLAKSFRVDKKIVDHIQAQF
PutGLP22	Pt_Chr0301173	MPASRASTALLALVALVLVCSSVPRVMAGDPSPLQDFCVADLMNPVFVNGFVCKNPKLVTANDFFRPGLNVPGKLNAQGSAVTLVSALQLAGLNTLGISLARIDFGPNGQNPPHTHPRATEILTVIKGQLLVGFVTSNQPDGRNLLFTKQLMEGDVFVFPQGLIHFQLNNGPVAAVAIAALSSQNPGVITIANAVFGSTPPISDLILAKAFMTEKDTVDWIQSQFAPAMSGNSSMGGGGGMGGGGMGGGGGNMTGGDGYYPGMVRKKP
PutGLP23	Pt_Chr0301199	MPASRASTALLALVALVLVCSSVPRVMAGDPSPLQDFCVADLMNPVFVNGFVCKNPKLVTANDFFRPGLNVPGKLNAQGSAVTLVSALQLAGLNTLGISLARIDFGPNGQNPPHTHPRATEILTVIKGQLLVGFVTSNQPDGRNLLFTKQLMEGDVFVFPQGLIHFQLNNGPVAAVAIAALSSQNPGVITIANAVFGSTPPISDLILAKAFMTEKDTVDWIQSQFAPAMSGNSSMGGGGGMGGGGMGGGGGNMTGGDGYYPGMVRKKP
PutGLP24	Pt_Chr0301174	MAASRALAALLAVVLLVVCSSVPRVLASDPGALQDFCVADLMNPVFVNGFVCKNPKMVTANDFFRPGLNVPGKLNAQGSAVTTVSVLQLPGLNTLGISLVRIDYGPNGQNPPHTHPRATEILTVIKGQLLVGFVTSNQPDGRNLLFTKQLVEGDVFVFPEGLIHFQLNNGPVAAVAIAALSSQNPGVITIANAVFGSTPPISDLI

		LAKAFMTEKATVDWIQSQFAPAMSGNSSMGGGGGGYYPGMRKKP
PutGLP25	Pt_Chr0301200	MAASRALAALLAVVLLVACSSVPRVLASDPGALQDFCVADLMNPVFVNGFVCKNPKMVTANDFFRPGLNVPGKLNAQGSAVTTVSVLQLPGLNTLGISLVRIDYGPNGQNPPHTHPRATEILTVIKGQLLVGFVTSNQPDGRNLLFTKQLVEGDVFVFPEGLIHFQLNNGPVAAVAIAALSSQNPGVITIANAVFGSTPPISDLILAKAFMTEKATVDWIQSQFAPAMSGNSSMGGGGGGYYPGMRKKP
PutGLP26	Pt_Chr0700805	MAACRALAALLAVVLLVVFSSVPRVLASDPGALQDFCVADLMNPVFVNGFVCKNPKMVTANDFFRPRLNVPGKLNAQVLQLPGLNTLGISMVRIDYGPNGQNPPHTHPRATEILTVIKGQLLVGFVTSNQADGRNLLFTKQLVEGDVFVFPEGLIHFQLNNGPVVAVAIAALSSQNPGVITIANAVFGSTPPISDLILAKAFMTEKATVDWIQSQFAPAMSGNSSMGGGGGGYNPGMGKKP
PutGLP27	Pt_Chr0700807	MAACRALAALLAVVLLVVFSSVPRVLASDPGALQDFCVADLMNPVFVNGFVCKNPKMVTANDFFRPGLNVPGKLNAQGSAVTTVSVLQLPGLNTLGISMVRIDYGPNGQNPPHTHPRATEILTVIKGQLLVGFVTSNQADGRNLLFTKQLVEGDVFVFPEGLIHFQLNNGPVAAVAIAALSSQNPGVITIANAVFGSTPPISDLILAKAFMTEKATVDCIQSQFAPAMSGNSGMGSGGSGYYPGMGKKP
PutGLP28	Pt_Chr0700806	MAACRALAALLAVVLLVVFSSVPRVLASDPGALQDFCVADLMNPVFVNGFVCKNPKMVTANDFFRPGLNVPGKLNAQGSAVTTVSVLQLPGLNTLGISMVRIDYGPNGQNPPHTHPRATEILTVIKGQLQVGFVTSNQADGRNLLFTKQLVEGDVFVFPEGLIHFQLNNGPVAAVAIAALSSQNPGVITIANAVFGSTPPISDLILAKAFMTEKATVDWIQSRFVPAMSGNSSMGGGGGGYNPGMGKKP
PutGLP29	Pt_Chr0700827	MAACRALAALLAVVLLVVFSSVPRVLASDPGALQDFCVADLMNPVFVNGFVCKNPKMVTANDFFRPGLNVPGKLNAQGSAVTTVSVLQLPGLNTLGISMVRIDYGPNGQNPPHTHPRATEILTVIKGQLLVGFVTSNQADGRNLLFTKQLVEGDVFVFPEGLIHFQLNNGPVAAVAIAALSSQNPGVITIANAVFGSTPPISDLILAKAFMTEKATVDWIQSQFAPAMSGNSSMGGGGGGYNPGMGKKP
PutGLP30	Pt_Chr0700808	MAASRALAALLAVVLLVVFSSVPRVLASDPGALQDFCVADLMNPVFVNGFVCKNPKMVTANDFFRPGLNVPGKLNAQGSAVTTVSVLQLPGLNTLGISLVRIDYGPNGQNPPHTHPRATEILTVIKGQLLVGFVTSNQADGRNLLFTKQLVEGDVFVFPEGLIHFQLNNGPVAAVAIAALSSQNPGVITIANAVFGSTPPISDLILAKAFMTEKATVDWIQSQFAPAMSGNSSMGGGGGGYNPGMGKKP
PutGLP31	Pt_Chr0604735	MAKLPTALLLATFTVLLLLAEPLLAGDPDMLQDICVADYKSLEGPLRLNGFPCKRPENVTVNDFFSGGLASPGNTGNSEVGSAVTAANVENLPGLNTLGVSMARIDFAPWGVNPPHTHPRATEIIFVLEGSLDVGFVTTANKLFTRFVCKGEVFVFPRGLVHLRNNGNSPAAVISAFNSQLQGTQSIAETLFAASPAVPADVLARAFQIDGGQVEAIRSKFAPQ
PutGLP32	Pt_Chr0604736	MHQELKNYLAMAKLPTALLLATFTVLLALAEPLLAGDPDMLQDICVADYKSLEGPLRLNGFPCKRPENVTANDFFSGMLASPGNTGNSEVGSAVTAANVENLPGLNTLGVSMARIDFAPWGVNPPHTHPRATEIIFVLEGSLDVGFVTTANKLFTRFVCKGEVFVFPRGLVHFQRNNGNSPAAVISAFNSQLQGTQSIAETLF

		AASPAVPADVLARAFQIDGGQVEAIRSKFAPQ
PutGLP33	Pt_Chr0604730	LPTALLATFAVLLALAEPLLAGDPDMLQDICVADYKSLEGPLRLNGFPCKRPENVTANDFFSGGLASP<br>GRNSEVGSTVTTASVENLPRLNTLCVSMARIDFAPWGPPARHRDHLQGSLDVGFVTTANKLFTRFVC<br>KGEVFVFPRGLVHFQRNNGNSPAAVISAFNSQLQGT
PutGLP34	Pt_Chr0403036	MAKLTAVILAACAAILALAVPLLAGDPDMLQDFCVADYKSLEGPLRLNGFPCKRPENVTANDFFSDV<br>LSLPGNTGNPLGSAVTAANVEKLPGLNTLGMSMSRVDYAPWGVNPPHTHPRATEIIYVLEGSLDVGF<br>VTTAGKLVARSMCKGELFVFPRGLLHFQKNNGGSPAVAISALNSQLPGTLSLAMAMFGSSPPVPTDVL<br>VRALQTDGGVKSIMAKLAAVIFAAFAALLAFATPLLAGDPDMLQDYCVADYKSLQGRE
PutGLP35	Pt_Chr0605775	MAPKQFSAVLLAACAVLLALPVPLLAGDPDMLQDFCVADYKSLDGPLRMNGFPCKKPESVTPNDFFS<br>DVLSLPGNTGNPVGSAVTVANVEKLPGLNTLGISMSRVDFGPGGVNPPHTHPRATEIIFVLEGSLDVGF<br>VTTSGKLFARTVCKGELFVFPRGLVHFQRNNGGVKAVAISAFNSQLPGTQYLATAMFGASPPVPTDVL<br>VRAFQVDGGMVEDIKSKFPPKYLAYMKHDD
PutGLP36	Pt_Chr0604084	MMARACSSLFLAAVAVALVLAVPSLAGDPDYLQDICVADLNSELKLNGFPCKANATAEDFFTGVLAK<br>PGATNTTSGSIVTGANVEKVPGLNTLGVSLSRIDYAPGGLNPPHTHPRATEVVFVLYGELDVGFITTAN<br>KLFAKTISQGDVFAFPRGLVHFQKNSADKPAAVISAFNSQLPGTQSIAMTLFGASPEVPDDVLAKAFQI<br>GTEEVDKIKAKFVPKS
PutGLP37	-	MARSYSSTLLIVAVALVLTAPLALAGDPDYLQDLCVADLNSEIKVNGFPCKANATADDFFSNAIAKPG<br>TITSPAGSVVTGANVEKIPGLNTLGVSLARIDYAPGGLNPPHTHPRATELIFVLYGTLDVGFITTANKLV<br>AKTVGAGEVFAFPRGLVHFQRNAGEEPAAVVSAFNSQLPGTQSIAMTLFGAAPEVSSDVLAKAFQIG<br>GEEVDKDKAKFAPKKG
PutGLP38	Pt_Chr0501398	MAMVSPFLPCAVMAVLLSVLPSPSIAGDPDLLQDICVADLTSGVKVNGFACKATVTEDDFYFKGLGA<br>AGNTNNTYGSVVTGANVEKVPGLNTLGVSMARIDYAPGGLNAPHTHPRATEMVFVLHGALDVGFIT<br>TGNKLVSKTIAAGDVFVFPRGLVHFQKNNGDGPASVISAFNSQFPGTQSLAMTLFGATPAVPDNVLTK<br>AFQVGTKEVEKIKSRLAPKKT
PutGLP39	Pt_Chr0402211	MARLHYLSVLCAVLLLALAAPSLGGDPDMLQDVCVADLASPVKLNGFPCKANISADDFFFAGLKKA<br>GNTDNAAGSAVTAANVQSFPGLNTLGVSMARIDYAPGGQNPPHTHPRATEIIFVTEGVLEVGFITTAN<br>KLFTKTVTVGEVFVFPRGLVHFQQNRGHGPASVIAGFNSQLQGTQAIAATLFAAAPPVPSDVLAKAFR<br>IDNGEVEAIKAKFT
PutGLP40	Pt_Chr0603689	MARLHLSVVCAVLLALAAPSLAGDPDMLQDVCVADLASPLRLNGFPCKANITADDFFFAGLKKAGN<br>TNNAAGSAVTGANVQSFPGLNTLGVSMARIDYAPGGQNPPHTHPRATEIIFVTKGVLEVGFITTANKL<br>FTKTVTVGEVFVFPRGLVHFQQNRGYGPASVIAAFNSQLQGTQSIATTLFAAAPPVPSDVLAKAFRIG<br>NGQVEAIKAKFK

PutGLP41	Pt_Chr0106250	MPISTYLLLYLCSFQIQEALARQNYTTSKLIAIQHPPWRGFTFCSSAPSFLALAAPSLAGDPDMLQDVCVADMESPIKLNGFPCKANITADDFFFAGLKKAGKTNNQAGSAVTAANVLSFPGLNTLGVSMARIDYAPGGQNAPHTHPRATEIIFVTKGVLEVGFITTANKLFTKTVTVGDVFVFPRGLVHFQQNRGHGPASVIAGFNSQLQGTQVLATTLLAATPPVPTDVLAKAFRVDNDMLEEVRALFK
PutGLP42	Pt_Chr0106263	MARLHLLLICAVLLALAAPSLAGDPDMLQDVCVADMESPIKLNGFPCKANITADDFFFAGLKKAGKTNNQAGSAVTAANVLSFPGLNTLGVSMARIDYAPGGQNAPHTHPRATEIIFVTKGVLEVGFITTANKLFTKTITVGDVFVFPRGLVHFQQNRGHGPASVIAGFNSQLQGTQVLATTLLAATPPVPTDVLAKAFRVDNDMLEEVRALFK
PutGLP43	Pt_Chr0106251	MAMLHLSVVCAVLFALAAPSLAGDPDLLQDVCVADLASPIKLNGFPCKANITADDFFFAGLKKAGNTNNQAGSAVTAANVLSFPGLNTLGVSMARIDYAPGGQNAPHTHPRATEIIFVTKGVLEVGFITTANKLFTKTITVGDVFVFPRGLVHFQQNRGHGPASVIAGFNSQLQGTQVLATTLLAATPPVPTDVLAKAFRIDNDQVEEVKALFK
PutGLP44	Pt_Chr0206684	MARHHHHVLFLLAAVLLPLAATVDPDAVQDYCVPDTHGHPVDLALLRTYGCKNPSNLTAGDFAFSGVRAAGNFSAATGFAGVSVTPAQFPGLHTLGMSFARADLSAAGGVNPPHYHPRATETALVLAGRVYAGFVDTGGRLFAKVLVKGDVMVFPRGMVHFQLNVGDAPATVYGTFNSENPGIVRIPATVFGSGIRDAVLERAFGLTPAELRRIESNFGPPKKTEMED
PutGLP45	Pt_Chr0105553	MVRTKATLWASSPVFVLATAVAVLVSACGADPEPVQDFCVAAVSKGADDDHPTFPGFPCKPESAVVSDDFFFAGLSRSGAAAANDSPFGSAVTPGNVEAFPGLNTLGVSINRVDLAPGGVNPLHSHPRAAELVHVVDGEMLVGFISTAGKFYSKMVGEGESFVIPRGLVHFQYNAGGKGKGAARAVTVFNSQLPGVVPAAPSLFGAEPEIPDAVLAKSFQVDGEIIKLLKSKFRN
PutGLP46	Pt_Chr0700256	MASWRSLALLVVLLAVSIFVTDASDPDILTDFVVPPGTNETLLDGAFFTYTDLITGFADSTKFTAIKATAAEFPALLGQSVSYAFLDFGAGTLNPPHIHPRASELLYVLQGPLMVGLVDETAGKVYTQTMQTGDMFVFPKGMVHFQYNAGAETARAFSAFGGASPGTVSLPSTVFESGIDDDVLAKSFHVDQATVEALKHDLAPAAPAPTAPAPATPAAPPPSNAAPVLSTRSALYTLVIGFGAAFLV
PutGLP47	Pt_Chr0700445	MASWRSLALLVVLLAVSIFVTDASDPDILTDFVVPPGTNATLLDGAFFTYTDLITGFADPAKFTATKATAAEFPALLGQSVSYAFLDFGAGTLNPPHIHPRASELLYVLQGPLMVGLVDETAGKVYTQTMQTGDMFVFPKGMVHFQYNAGAETARAFSAFGGASPGTVSLPSTLFESGIDDDVLAKSFHVDQATVEALKHDLAPAAPAPTVPSPATPAAPPPSNAATVLSTRSALYTLVIGFGAAFLV
PutGLP48	Pt_Chr0700255	MSFYSLVLVVVALASAPLAAVAGDPDILTDFVLPTANGGQVTGDYFTFTGFRPAPPATPPPTAFTITKASMAEFPALNGQSVSYARLTFPAGTVNPTHTHPRAAELLLVVEGALSVGFIDTAGRLYTKDLVAGDMFVFPKGLVHYQSNQGPNLAVALSAFGSASAGTVSVPVTVFGTGIDDAVLAKSFKTDVATVQKLKAALTPPPKN

	PutGLP49	Pt_Chr0700444	MASMSFYSLVLVVVALASAPLAAVAGDPDILTDFVLPTANGGQVTGDYFTFTGFRPAPPATPPPTAFTITKASMAEFPALNGQSVSYARLTFPAGTVNPTHTHPRAAELLLVVEGALSVGFIDTAGRLYTKDLVAGDMFVFPKGLVHYQSNQGPNLAVALSAFGSASAGTVSVPVTVFGTGIDDAVLAKSFKTDVATVQKLKAALTPPPKN
	PutGLP50	Pt_Chr0700250	MATTHFYYFVPVVVALVSTPHAVVAGDPDILTDFVMPMNITGDYFTYGGFRPKEELPKASMAEFPVLNGQSVSYAMLRFPPGTVNPTHTHPRAAELRLVIEGALSAGKLYTKDLVVGDMFIFPKGLRQCGHCIRACHTVFGTGVDDTVLVKSFKTDLPTVQKLKAALTPPPKN
	PutGLP51	Pt_Chr0700437	MATTHFYYFVPVVVALVSTPHAVVAGDPDIFTDFVMPMNITGDYFTYGGFRPKEELPKASMAEFPVLNGQSVSYAMLRFPPGTVNPAHTHPRAAELRLVIEGALSVGFIDTAGKLYTKDLVVGDMFIFPKGLRQCGHCIHACHTVFGTGVDDAVLVKSFKTDLPTVQKLKAALTPPPKN
	PutGLP52	Pt_Chr0700439	MASMHFYSLVLVVVALVSTPLAVVAGDGDPDILTDFVMPMMLGMPMNITGDYFTYTGFRTKEELPKASMAEFPALNGQSVSYAVLKFPPSTVNPTHTHPRAAELLLVIEGALSVGFVDTAGKFYTKDLVVGDMFVFPKGLVHYQSNQGPNLAIALSAFGSANAGTVSVPVTVFGTGVDDAVLAKSFKTDLRPTVQKLKAALTPPPKN
	PutGLP53	Pt_Chr0700249	MASMHFYSLLLVVVALVSTPLVVVAGDPDILTDFVMPMMWGMPMNITGDYFTYTGFRTKELPVIGFSAQKASMAEFPALNGQSVSYAMLRFPPGTVNPTHTHPRAAELLLVIEGALSVGFVDTAGKLYTKNLIVGDMFVFPKGLVHYQSNQGTNLAIALSAFGSANAGTVSVPVTVFGTGVDDAVLAKSFKTDLPTVQKLKAALTPPPKN
	PutGLP54	Pt_Chr0700435	MASMHFYSLLLVVVALVSTPLVVVAGDPDILTDFVMPMMWGMPMNITGDYFTYTGFRTKELPVIGFSAQKASMAEFPALNGQSVSYAMLRFPPGTVNPTHTHPRAAELLLVIEGALSVGFIDTAGKLYTKDLVVGDMFIFPKGLVHYQSNQGTNLAIALSAFGSANAGTVSVPVTVFGTGVDDAVLAKSFKTDLPTVQKLKAALTPPPKN
Zea mays	ZmGLP1	Zm00001eb267120_P002	MAAAIVLSGQVRPLPSSLPLSLLLLLLLCCSGTSWGWSTSRGGAARECGFDGKLEALEPRHKVQSEAGSVQYFSRFNEADRELTCAGIFAVRVVVDAMGLLLPRYSNVHSLVYIVQGRGIIGFSFPGCQEETQQQQYGYGYGYGHHHHQHDHHKIHRFEQGDVVAMPAGAQHWLYNDGDAPLVAVYVFDENNNINQLEPSMRKFLLAGGFSKGQPHFAENIFKGIDARFLSEALGVSMHVAEKLQSRRDQRGEIVRVEPEHGFHQLNPSPSSSSFSFPSSQVQYQTCQRDVDRHNVCAMEVRHSVERLDQADVYSPGAGRITRLTSHKFPVLNLVQMSAVRVDLYQDAIMSPFWNFNAHSAMYGIRGSARVQVASDNGTTVFDDVLRAGQLLIVPQGYLVATKAQGEGFQYIAFETNPDTMVSHVAGKNSVLSDLPAAVIASSYAISMEEAAELKNGRKHELAVLTPAGSGSYQQGQAGSAQQ
	ZmGLP2	Zm00001eb052450_P001	MVSARIVVLLATLLCAAAAVASSWEDDNHHHHGGHKSGQCVRRCEDRPWHQRPRCLEQCREEEREKRQERSRHEADDRSGEGSSEDEREQEKEKQKDRRPYVFDRRSFRRVVRSEQGSLRVLRPFDEVSRLL

		RGIRDYRVAVLEANPRSFVVPSHTDAHCICYVAEGEGVVTTIENGERRSYTIKQGHVFVAPAGAVTYLANTDGRKKLVIAKILHTISVPGEFQFFFGPGGRNPESFLSSFSKSIQRAAYKTSSDRLERLFGRHGQDKGIIVRATEEQTRELRRHASEGGHGPHWPLPPFGESRGPYSLLDQRPSIANQHGQLYEADARSFHDLAEHDVSVSFANITAGSMSAPLFNTRSFKIAYVPNGKGYAEIVCPHRQSQGGESERERDKGRRSEEEEEESSEEQEEAGQGYHTIRARLSPGTAFVVPAGHPFVAVASRDSNLQIVCFEVHADRNEKVFLAGADNVLQKLDRVAKALSFASKAEEVDEVLGSRREKGFLPGPKESGGHEEREQEEEEEREERHGGRGERERHGREEREKEEEEEREGRHGRGRREEVAETLMRMVTARM
ZmGLP3	Zm00001eb202970_P001	MAIRRLFLFISMLLAMACSCAIASDPSLLQDFCVADKTSQVRVNGFACKDAKDVVAEDFFFSGLHKAGNTSNRQGSAVTAANVAQIPGLNTMGISMVRIDYAPKGLNPPHTHPRATEMLAVLEGSLYVGFVTSNPNNTLVSKVLSKGDVFVFPKGLVHFQYNYGTDSAAALAALSSQNPGVITVANTVFGSDPLISDDVLAKAFQVDQKAVNWIQAQF
ZmGLP4	Zm00001eb266290_P002	MAAVDLTPRTPKKAYGGDGGAYYEWSPADLPMLAVASIGAAKLSLAAGGLSLPSYSDSAKVAYVLQGVGTCGLVLPEATKEKVVAVKEGDALALPFGAVTWWHNGPAAQADLTVLFLGDTSKGHKRGQFTNFQLTGSAGIFTGLSTEFVSRAWDLPEPDAARLVSSQPASGIVKLPASAAALPAPSPQDRAGVALNCLEAPLDVDIPGGGRVVVLNTANLPLVREVGLGADLVRIDAHSMCSPGFSCDSAYQVTYIVRGSGRVQVVGPDGRRVLETRIEGGSLFIVPRFHVVSKIADASGMEWFSIITTPNPVFSHLAGKTSVWKAISPEVLQASFNTTPEMEKLFRSKRLDSEIFFAPPSSN
ZmGLP5	Zm00001eb171510_P001	MAASTYFLLVAFLALVTSQSIASDPSPLQDFCVADKYSPVKVNGFVCKDPMAVNADDFFKAAKLDQPRDTKNKVGSNVTLINVMQLPGLNTLGISLARIDYAPLGQNPPHTHPRATEILTVLEGKLYVGFVTSNQANSNNKLFTKVLNKGDVFVFPQGLIHFQFNPVHDKPAVAIAALSSQNPGVITIANAVFGSKPPISDDVLAKAFQVQKGTIDWLQAQFWENNHY
ZmGLP6	Zm00001eb171500_P001	MAALTYFLLVAFLALVTSQAIASDPSPLQDFCVADKYSPVKVNGFVCKDPMAVNADDFFKAAKLDQPRDTKNKVGSNVTLINAMQLPGLNTLGISLARIDYAPLGQNPPHTHPRATEILTVLEGKLYVGFVTSNQANNNNKLFTKVLNKGDVFVFPQGLIHFQFNPVHDKPAVAIAALSSQNPGVITIANAVFGSKPPISDDVLAKAFQVQKGTIDWLQAQFWENNHY
ZmGLP7	Zm00001eb129440_P001	MAKVHLYVAAACAVVLALATPALAGDPDMLQDVCPADYASPVKLNGFACKANFSADDFFFDGLRNPGNTNNPAGSVVTAANVEKFPGVNTLGVSMARIDYAPGGQNPPHTHPRATEIIFVLEGTLEVGFITTANALFTKTVTKGDVFVFPRGLVHFQQNRGHGPAAVVAAFNSQLQGTQAIAMTLFGAVPPVPSDILAKAFRISSGEVDHIKANFAPK
ZmGLP8	Zm00001eb171650_P001	MAASTYFLLVAFLALVTSHAIASDPSPLQDFCVADKDSPVKVNGFVCKDPMAVNADDFFKAAKLDQPRDTTSKVGSNVTLINVLQLPGLNTLGISLARIDYAPLGQNPPHTHPRATEILTVLEGTLYVGFVTSNQADKSNKLFAKVLNKGDVFVFPQGLIHFQFNPVPDKPAVALAALSSQNPGAITIANAVFGSKPPISNDVLA

		KAFQVQKGTIDWLQAQFWENNHN
ZmGLP9	Zm00001eb132730_P001	MVHRTSIADVHVMCMDLSPKKPNKASASDGGAYYDWSPADLPMLGVASIGAAKLCLTAGGLALPSYSDSAKIAYVLQGKGIFGVVLPEATKEKVISVKEGDALALPFGVVTWWHNNADAAISDLVVLFLGDTSTGHKPGQFTNFQLTGSTGIFTGFSTEFVARAWDLTQDDAAKLVSTQPGSGIVRVKDGHKMPEARDEDRQGLVLNCLEAPLDVDIKNGGRVVVLNTQNLPLVKEVGLGADLVRIDAHSMCSPGFSCDSAYQVTYIVRGSGRVQVVGIDGTRVLETRAEGGCLFIVPRFFVVSKIADETGMEWFSIITTPNPIFSHLAGRTSVWKAISPAVLQSSFNTTPEMEKLFRSKRLDSEIFFAPSN
ZmGLP10	Zm00001eb355570_P001	MAPVDLTPKQPRKAYGGDGGAYYEWSPADLPMLGVASIGAAKLSLAAGGLALPSYSDSAKVAYVLQGTGTCGIVLPEATKEKVVAVKEGDALALPFGVVTWWHNGPAAPTPLTVLFLGDTSKGHRPGQFTNFQLTGATGIFTGFSTEFVSRAWDLPEADAAALVSSQPASGIVRASSSPLPAPSPRDREGVALNCLEAPLDVDIPGGGRVVVLNTANLPLVKEVGLGADLVRIDAHSMCSPGFSCDSAYQVTYIVRGSGRVQVVGPDGVRVLETRVEGGFLFIVPRFHVVSKIADASGMEWFSIITTPNPIFSHLAGKTSVWKAISAEVLQASFNTTPEMEKLFRSKRLDSEIFFAPPSN
ZmGLP11	Zm00001eb171540_P001	MAASTYFLLVAFLALVTSHAIASDPSPLQDFCVADKDSPVKVNGFVCKDPMAVNADDFFKAAKLDQPRDTTKSKVGSNVTLINVLQLPGLNTLGISLARIDYEPLGQNPPHTHPRATEILTVLEGTLYVGFVTSNQADRSNKLFAKVLNKGDVFVFPQGLIHFQFNPAHDKPAVALAALSSQNPGAITIANAVFGSKPPISDDVLAKAFQVQKGTIDWLQAQFWENNHY
ZmGLP12	Zm00001eb413210_P001	MASCSFHLLSVILIVIASSGAWATDPSPLQDFCVADKESPVRVNGLPCKDIKDVKVDDFFLAADLDKPRDTTMSKVKSNVTLINVMKLAGLNTLGISMARIDYAPQGQNPPHTHPRATEILTVLEGSLYVGFVTSNPDNKFISKLLNKGDVFVFPQGLIHFQFNPSHDKPAVAIAALNSQNPGAITISNAVFGSKPPIADDVLAKAFQVDKKVVDWLQAQFWEDNHN
ZmGLP13	Zm00001eb413180_P001	MVSSNFYLLSVLLIAIASGARATDPSPLQDFCVADKESTVRVNGLPCKDIKDVKVDDFFLAADLDKPRDTTTNKVKSNVTLINVMKLAGLNTLGISMARIDYAPQGQNPPHTHPRATEILTVLEGSLYVGFVTSNPDNKFFSKLLNKGDVFVFPQGLIHFQFNPSHDKPAVALAALSSQNPGAITISNAVFGSKPPIADDVLAKAFQVDKKVVDWLQAQFWEDNHN
ZmGLP14	Zm00001eb088410_P001	MASSLVLIAAALLALASSWQQAIAYDPSPLQDFCVADKNSPVRVNGFPCKDPMAVTPDDFFNAAMIIDKRRDTNNKVGSNVTNVNVESFPGLNTLGISLARIDYAPLGVNPPHIHPRATELLTVLEGTLYLGFVTSNPNRLFSKVVKKGDVFVFPKAMIHFQMNLDHDKPAAALSSLSSQNPGVITIASAVFGSKPPISDDVLAKAFQVEKKLIDWLQSQFWDTNY
ZmGLP15	Zm00001eb413190_P001	MASSSFYLLSVLLIVIASCAWATDPSPLQDFCVADKESPVRVNGFPCKDVKDVKVDDFFLAADLDKPRDTTMSKVKSNVTLINVMKLAGLNTLGISMARIDYAPQGQNPPHTHPRATEILTVLEGSLYVGFVTSNPDNKFISKLLNKGDVFVFPQGLIHFQFNPSHDKPAVAIAALNSQNPGAITISNAVFGSKPPIADNVLAKAF

		QVDKKVVDWLQAQFWEDNHN
ZmGLP16	Zm00001eb171660_P001	MAASTFLLIAFLALITSQAIASDPSPLQDFCVADKDSPVKVNGFVCKDPMAVNADDFFKAAKLDQPRDTTKSKVGSNVTLINVMQLPGLNTLGISLARIDYAPLGQNPPHTHPRATEILTVLEGTLYVGFVTSNQADRSNRLFAKVLNKGDVFVFPQGLIHFQFNPVHDKPAVALAALSSQNPGAITIANAVFGSKPPISDDVLAKAFQVQKGTIDWLQAQF
ZmGLP17	Zm00001eb171610_P001	MAASTVLLVAFLALVTSHAIASDPSPLQDFCVADKDSPVKVNGFVCKDPMAVNADDFFKAAKLDQPRDTTKSKVGSNVTLINVMQLPGLNTLGISLARIDYAPLGQNPPHTHPRATEILTVLEGTLYVGFVTSNQADRSNKLFAKVLNKGDVFVFPQGLIHFQFNPVHDKPAVALAALSSQNPGAITIANAVFGSKPPISDDVLAKAFQVQKGTIDWLQAQFWENNHY
ZmGLP18	Zm00001eb171520_P001	MTASTFLLVAFLALVTSQAIASDPSPLQDFCVADKDSPVKVNGFVCKDPMAVNADDFFKAAKLDQPRDTTKSKVGSNVTLINVMQLPGLNTLGISLARIDYAPLGQNPPHTHPRATEILTVLEGTLYVGFVTSNQADRSNKLFAKVLNKGDVFVFPQGLIHFQFNPVHDKPAVALAALSSQNPGAITIANAVFGSKPPISDDVLAKAFQVQKGTIDWLQAQFWENNHY
ZmGLP19	Zm00001eb332460_P001	MARIHLYVVAAACAVALALAGDPDMLQDVCVADYASPVKLNGFPCKANVSADDFFFDGLRSPGNTNNPAGSAVTAANVDKFPGVNTLGVSMARIDYAPGGQNPPHTHPRATEIIFVLEGTLEVGFLTTANRLLSKTVATGDVFVFPRGLVHFQQNRGHGPAAVVAAFNSQLQGTQAIAMTLFGATPPVPTDILAKAFRVGSGEVEHIKANFAPK
ZmGLP20	Zm00001eb171600_P001	MAASTVLLVAFLALVTSHAIASDPSPLQDFCVADKDSPVKVNGFVCKDPMAVNADDFFKAAKLDQPRDTTKSKVGSNVTLINVMQLPGLNTLGISLARIDYAPLGQNPPHTHPRATEILTVLEGTLYVGFVTSNQADRSNKLFAKVLNKGDVFVFPQGLIHFQFNPVHDKPAVALAALSSQNPGAITIANAVFGSNPPISDDVLAKAFQVQKGTIDWLQAQFWENNHY
ZmGLP21	Zm00001eb171570_P001	MAASTFLLVAFLALITSQAIASDPSPLQDFCVADKDSPVKVNGFVCKDPMAVNADDFFKAAKLDQPRDTSKSKVGSNVTLINVMQLPGLNTLGISLARIDYAPLGQNPPHTHPRATEILTVLEGTLYVGFVTSNQADRSNKLFAKVLNKGDVFVFPQGLIHFQFNPVHDKPAVALAALSSQNPGAITIANAVFGSKPPISDDVLAKAFQVQKGTIDWLQAQFWENNHY
ZmGLP22	Zm00001eb419900_P001	MASLVALLAVALVAQLASWQQAAVAYDPSPLQDFCVADKNSPVRVNGFPCKDPMAVTPDDFFNPAMIIGKRRDTNNKVGSNVTNVNVESFPGLNTLGISLARIDYAPLGVNPPHIHPRATELLTVLEGTLYLGFVTSNPNRLFSKVVKKGDVFVFPKAMIHFQMNLDHEKPAAALSSLSSQNPGVITIASAVFGSKPPISDDVLAKAFQVEKKLIDWLQSQFWDTNY
ZmGLP23	Zm00001eb171630_P001	MAASTYFLLVAFLALVTSHAIASDPSPLQDFCVADKDSPVKVNGFVCKDPIAVNADDFFKAAKLDQPRDTTKSKVGSNVTLINVMQLPGLNTLGISLARIDYMPLGQNPPHTHPRATEILTVLEGTLYVGFVTSNQADRSNKLFAKVLNKGDVFVFPQGLIHFQFNPAHDKPAVALAALSSQNPGAITIANAVFGSKPPISDDV

		LAKAFQVQKGTIDWLQAQFWENNHY
ZmGLP24	Zm00001eb171590_P001	MPLPLILARSKTSVLPTKTLQVNGFVCKDPMAVNADDFFKAAKLDQPRDTTKSKVGSNVTLINVMQLPGLNTLGISLARIDYAPLGQNPPHTHPRATEILTVLEGTLYVGFVTSNQADRSNKLFAKVLNKGDVFVFPQGLIHFQFNPVHDKPTVALAALSSQNPGAITIANAVFGSKPPISDDVLAKAFQVQKGTIDWLQAQFWENNHY
ZmGLP25	Zm00001eb413250_P001	MAASSYFLLAAFLAVTASHATASDPSPLQDFCVADIHSPVKVNGFVCKDPMAVIPDDFFKAANLDKARDTMKSKVGSNVTLINVMQLPGLNTLGISLARIDYAPLGENPPHTHPRATEILTVLEGTLYVGFVTSNPNKLFAKVLNKGDVFVFPQGLIHFQFNPVYDKPAVAIAALSSQNPGVITIANAVFGSKPPISDDVLAKAFQVEKGTIDWLQAQFWENNHY
ZmGLP26	Zm00001eb171640_P001	MAASTFLLVAFLALITSQAIASDPSPLQDFCVADKDSPVKVNGFVCKDPMAVNADDFFKAAKLDQPRDTTKSKVGSNVTLINVMQLPGLNTLGISLARIDYAPLGQNPPHTHPRATEILTVLEGTLYVRFVTSNQADRSNKLFAKVLNKGDVFVFPQGLIHFQFNPVHDKPAVALAALSSQNPGAITIANAVFGSKPPISDDVLAKAFQVQKGTIDWLQAQFWENNHY
ZmGLP27	Zm00001eb401380_P001	MAPVDLTPRQPRKAYGGDAGAYYEWSPADLPMLGVASIGAAKLSLAAGGLSLPSYSDSSKVAYVLEGTGTCGIVLPEATKEKVLAVKEGDALALPFGVVTWWHNGPAAPTQLTVLFLGDTSKGHRPGQFTNFQLTGASGIFTGFSTEFVSRAWDLPEANAAALVSSQPASGIVRASSPLPAPSAQDREGVALNCLEAPLDVDIPGGGRVVVLNTANLPLVREVGLGADLVRIDAHSMCSPGFSCDSAYQVTYIVRGSGRVQVVGPDGVRVLETRVEGGFLFIVPRFHVVSKIADASGMEWFSIITTPNPIFSHLAGKTSVWKAISAEVLQASFNTTPEMEKLFRSKRLDSEIFFAPPSN
ZmGLP28	Zm00001eb171530_P001	MAASTYFLLVAFLALVTSHAIASDPSPLQDFCVADKDSPVKVNGFVCKDPMAVNADDFFKAAKLDQPRDTTKSKVGSNVTLINVLQLPGLNTLGISLARIDYAPLGQNPPHTHPRATEILTVLEGTLYVGFVTSNQADRSNKLFAKVLNKGDVFVFPQGLIHFQFNPIHDKPAVALAALSSENPGAITIANAVFGSKPSISDDVLAKAFQVQKGTIDWLQAQFWENNHY
ZmGLP29	Zm00001eb171550_P001	MAASTFLLVAFLALITSQAIASNPSPLQDFCVADKDSPVKVNGFVCKDPMAVNADDFFKAAKLDQPRDTSKSKVGSNVTLINVMQLPGLNTLGISLARIDYAPLGQNPPHTHPRAIEILTVLEGTLYVGFVTSNQADRSNKLFAKVLNKGDVFVFPQGLIHFQFNPVHDKPAVALAALSSQNPGAITIANAVFGSKPPISDDVLAKAFQVQKGTIDWLQAQFWENNYY
ZmGLP32	Zm00001eb301380_P001	MAAIPSLLRRLAIVVATTAMVILLPSPSVAGDPDLLQDICVADLTSTVKVNGYACKAAAAVTADDFYFSGLGGAGNTSASAYGSAVTGANVEKVPGLNTLGVSMSRIDYAPGGGLNPPHTHPRATEMVFVLQGTLDVGFVTAANNRLVARTLAPGDVFVFPRGLVHFQRNAGDGPAAVLSAFNSQLPGTQSLAAALFAASPELPDAVLAKAFQVGTKEVDKIKARLAPKKA
ZmGLP33	Zm00001eb36	MANKLATSSLLAAAVLLMALAAPSLAGDPDMLQDICVADYKSLKGPLRLNGFPCKRPENVTACDFFS

	2850_P001	GALASAGNTANALGSAVTAASADTLPGLNTLGVSLSRIDYAPWGVNPPHVHPRATEVIFVLQGSLDVGFVAAASNRLYARTVSAGDVFVFPRGLVHYQRNRGGDPAVALSAFDSQLPGTQPVAEALFGSSPPVPTDVLARSFHVDGGLVEAIRSKFPPK
ZmGLP34	Zm00001eb070560_P001	MAYRAFATLLAVLAFVGFASVPRGLATDPTQLQDFCVADNKNPVLVNGVVCKNPNVVKATDFFFRIVPMAPNGQGSAVTPVAVNEIPGLNTLGISLARIDFVPGGQNPPHTHPRGSEILTVIQGTLLVGFVTSNQLLNNTLFTMQLNMGDVFVFPQGLIHFQLNNGKTPAVAIAALSSQNPGTVTIANAVFGAKPPILDDILARAFMLEKATVDWVQQAFGAAPVAGGGGGLPGGGGYPGSGGGLPGGGGYPGSGFPGYPGRS
ZmGLP35	Zm00001eb053700_P001	MEKYTKALASLLTMLVLAPLLAMATDPDPLQDFCVADLNGKPSVNGYPCQPPSSAGDQFLFSTKIATGGDPLANPNGSNVTELDVSEWPGVNTLGVSMNRVDFAPGGTNPPHVHPRATEVGLVTRGELLVGIVGSLDSGNRYYSKVVRAGETFVIPRGLVHFQFNVGKEDATMVVSFNSQKPGIIFVPLTLFGSSPPIPTPVLSKALRVDASVVDLIKSKFTGGY
ZmGLP36	Zm00001eb266290_P003	MAAVDLTPRTPKKAYGGDGGAYYEWSPADLPMLAVASIGAAKLSLAAGGLSLPSYSDSAKVAYVLQGVGTCGLVLPEATKEKVVAVKEGDALALPFGAVTWWHNGPAAQADLTVLFLGDTSKGHKRGQFTNFQLTGSAGIFTGLSTEFVSRAWDLPEPDAARLVSSQPASGIVKLPASAAALPAPSPQDRAGVALNCLEAPLDVDIPGGGRVVVLNTANLPLVREVGLGADLVRIDAHSMCSPGFSCDSAYQVTYIVRGSGRVQVVGPDGRRVLETRIEGGSLFIVPRFHVVSKIADASGMEWFSIITTPK
ZmGLP37	Zm00001eb287560_P001	MAPKLPVVLLAACAVLLALATPLLAGDPDMLQDICVADYESLEGPLRVNGFPCKREANVTADDFFFGGLAAAADVYSGNPMGSAATAADVSVLPGLNTLGVSMARTDFAPSGGVSPPHVHPRATEILFVVEGTLEVGFVTSANRLFSRAVGKGEVFVFPRGLVHFQRIVGAAPAVAISAFNAQLPGTQTVAGALFGAAPAVPTDVLARALQIDGGAIEYIKSKFAPN
ZmGLP38	Zm00001eb401000_P001	MAVATTARWLLLLAVVSAAAASGKHERWRVGGQVVEKERRRVVAESEAGSVSAVDVADAAGTAYRLHFITMDPGALFLPVQLHADMVFYVHSGRGKVTSIEEESSEQSSLEVERGDVYNFEQGSILYIQSYPNASRQRLRIYAIFTSEGINADDPSKPKVEAYSSVSNLVKGFETDVLRLGFGVKPEVVEAIKSAKTPPPIIAYNPEEEKGDKKPGWTENIIDALLGVRDPEEFLNKKKKKKDKHKDKKSKSKAFNFYSGKPDVQNCYGWSRMMTSKDLDALHGSSIGMFMVNLTTGSMMGPHWNPKATEIAIVTEGSGIVQTVCPSSSSSSSSPSGGSSGDHHHGHKRRGGPGGRGDEGEGEGGRARWQCRNSVFRVKEGDVFVVPRFHPMAQMSFNDDSFVFVGFSTHMGQNHPQFLAGKGSVLQAIGKKVLALALGQRDPTAVDKLLSAQRESTILPCVSCAEELAEKDRENVRRKRRGGNGRRRKRRGRKGNGRKRRGRKGNGRKKRGGKRKKSGPGRSKRSSGGERKRRRSVHGDARRKKERGRRKKNGGGRRKKAVTSRHTGCPRN
ZmGLP39	Zm00001eb232890_P001	MASVVVRPSKLLLALLVAAMAASPRALAYDPSPLQDFCVADTASNVFVNGQACKDPAQVTAADFAFSGLQDAGDTGNAFGSKVTLVDARALPGLNSLGVAMARLDIAPGGVNPPHTHPRATEVLTVVEGQMYAGFLATDGKLFARVLNRGDAFVFPRGLVHFEFNCGAGPAVGLAGLSSQNPGLVRVADSLFGAAPAVT

		DEVLAKAFRIDAATVQRIKAQFAKK
ZmGLP40	Zm00001eb016090_P001	MDRLTVMAPPLLVLLLLLLSRCSAAASRRGGGWWEEGEGEWRPSEEEEKGKGKGRGLFLLHRVEKVVESEGGQVRVVRGQPWPPASFACREGLMHIGFITMEPKTLFVPQYLDSSITLFVQRGEAKVGYIHKDELVERKLKMGDVLHIDAGSTFYMVNPGKGQRLQIICSVDASDSLGFGPPYQAFFLGGAGDPASVIAGFGPKTLTRAFNATYDELARILLPRTGGPIVYYTADAEPESGAAEEERGQVDGHDGVLDRGARREGAGAWVPGGRGDGGDECGGSDDAREATWWWTKLVNRVVGGAAGGGGAAEANRKGKKKKGGAPEPYNLYDSEPGFRNAYGWTVSVDKHQYEPLKHPDIGVYLVNLTAGSMLAPHVNPRATEYGVVLGGEGTVQVVFPNGSLAMSEVVRPGDVFWIPRYFPFCQVAARAGPFEFFGFTTSARRNRPQFLVGASSVLRTMLGPEIAAAFGAREKEFSKLVRAQREALIMPSSPGKEEEEHGKKGREKEESLPMVVEQAAAE
ZmGLP41	Zm00001eb155450_P001	MASAARSAALRVLLLVAVAIAGALSDPTPLQDTCVADMRAATPVDGFACKPQSAVVDEDFFSRAIASAASTANPFGANSTRATVATFPGLNTLGVSITRVDLAPGGLNPPHSHPRASELVMVLQGEVLVGFTTGANRLFSKVVRENELFVVPRGLQHFQLNTGAGDAVFVAMFDSQSPGVVTPTFAMFSTKPAMPMEVLTKTFLTGEDQVNAIMSKFAGF
ZmGLP42	Zm00001eb060460_P001	MKVPVLLLLVSLCFSLALAWQTDTESGSGRPYHYGEESFRHWTRSRQGRFRVLERFTHELLEDAVGNYRVAELEAAPRAFLQPSHYDADEVMFVKEGEGVIVLLRGGKRESFCVREGDVMVIPAGAVVYSANTHQSEWFRVVMLLSPVVSTSGRFEEFFPIGGESPESFLSVFSDDVIQASFNTRREEWEKVFEKQSKGEITTASEEQIRELSRSCSRGGRSSRSEGGDSGSSSSKWEIKPSSLTDKKPTHSNSHGRHYEITGDECPHLRLLDMDVGLANIARGSMMAPSYNTRANKIAIVLKGQGYFEMACPHVSGGRSSPRRERGHGREEEEEREEEQGGGGGQKSRSYRQVKSRIREGSVIVIPAGHPTALVAGEDKNLAVLCFEVNASFDDKVFLAGTNSALQKMDRPAKLLAFGADEEQQVDRVIGAQKDAVFLRGPQSHRVSSV
ZmGLP43	Zm00001eb061410_P001	MSKPASSSFLLLLLSATLLATVCRADPDPVQDFCVAVAPGGHNNASSSSSTYPGFPCKPASTVVSDDFFFAAHAGGASTDNPMGAGVTPGNVEAFPGLNTLGLSINRVDLAPGGVNPLHTHPRSAELVHVEAGEMLVGFVSTEGRFYSKVVRAGESFVIPRGMVHFQYNVGKGAARAMTVFNSQLPGVVLAAQTLFGADPEIPDDVLAKSFQVDADTIKLLKSKFQKG
ZmGLP44	Zm00001eb171560_P001	MPLGQNPPHTHPRATEILTVLEGTLYVGFVTSNQADRSNKLFAKVLNKGDVFVFPQGLIHFQFNPAHDKPAVALAALSSQNPGAITIANAVFGSKPPISDDVLAKAFQVQKGTIDWLQAQFWENNHY
ZmGLP45	Zm00001eb242750_P001	MATRHLLLLLLAALLPAAATADPDAVQDYCVPDTGGGRGRGRAVDLALLPSYPCRSPANLTAADFAFAGVRAAGNFSADTGFAGVSVTPAQFPALHTLGVSFARADLSAAGGVNPPHYHPRATETALVLAGRVYAGFVDSGGRVFAKVLEKGEVMVFPRAMVHFQMNVGDEPATVYGTFNSENPGIVRIPATVFGSGIKDAVLERAFGLEPEELRRLQKRFGPPPKTKTAEMDD
ZmGLP46	Zm00001eb171620_P001	LSLVRTPLACQPLCLRRLGHPFTDQIHIANGLTVDDPDSSLSSGQEFVIPLPLKVNGFDCKDPLAVNVDDFFKAAKLDQEQGQNPQHTHPRATEILTVLEGTLYVGIVTSNQADRSNKLFVKVLNKGDVFVFPQGL

		IHLQFNPVHDKPVVALAALSSQNPRAITIANAVFGSKPPISDDVLAKAFQVQKGSID
ZmGLP47	Zm00001eb35<br>5570_P002	MMLLEQRKRTTGTGTCGIVLPEATKEKVVAVKEGDALALPFGVVTWWHNGPAAPTPLTVLFLGDTS<br>KGHRPGQFTNFQLTGATGIFTGFSTEFVSRAWDLPEADAAALVSSQPASGIVRASSSPLPAPSPRDREG<br>VALNCLEAPLDVDIPGGGRVVVLNTANLPLVKEVGLGADLVRIDAHSMCSPGFSCDSAYQVTYIVRG<br>SGRVQVVGPDGVRVLETRVEGGFLFIVPRFHVVSKIADASGMEWFSIITTPNPIFSHLAGKTSVWKAIS<br>AEVLQASFNTTPEMEKLFRSKRLDSEIFFAPPSN
ZmGLP48	Zm00001eb26<br>6290_P001	MAAVDLTPRTPKKAYGGDGGAYYEWSPADLPMLAVASIGAAKLSLAAGGLSLPSYSDSAKVAYVLQ<br>GVGTCGLVLPEATKEKVVAVKEGDALALPFGAVTWWHNGPAAQADLTVLFLGDTSKGHKRGQFTNF<br>QLTGSAGIFTGLSTEFVSRAWDLPEPDAARLVSSQPASGIVKLPASAAALPAPSPQDRAGVALNCLEAP<br>LDVDIPGGGRVVVLNTANLPLVREVGLGADLVRIDAHSMCSPGFSCDSAYQVTYIVRGSGRVPLHRA<br>PLPCRVQDRRRVRDGVVLHHHHPEPGVQPPGGEDVGVEGHLAGGAAGVLQHHSGDGEAVPLQEAR<br>LGDLFRASQLQLKHQLSKTGERRSFSIVVKVMVINN
ZmGLP49	Zm00001eb00<br>5890_P001	MSATADGGSIGTGMSHRIIVALFLSAALCRCDPDLLLDYCVADTSSSQLHLNGLACIDPASARAEHFAT<br>SALSRASETETAYGFSVTVTSPASSLPGANAQGLAMARTDLAPGGLAPPHTHPRASEVALVLAGSVLV<br>GFADTSYRLYTQLLRAGEAFVVPRGMVHFMYNVDVAAPAVVLSGLNSQSPGAQLVPFSAFRTEPRVP<br>DEVLKMAFRINGQDVQRIQRNLGGSS
ZmGLP50	Zm00001eb04<br>0300_P001	MAPSKAVVAHLVVLLVPLLSLLPFSSHALTQDLCVANRLLPDTPSGYPCKPKGLVSSDDFYSDALARP<br>GPVIAPFNTSLASAAVKQLPGLNGLGISATRVDVRPGGGVPMHTHPEASEVMFVLEGTFSAGFISAET<br>NKAYVKSLKKGDLYVFPQGLLHFQFNTGNTTATAIAAYSNQNPGLQIAVYALFGNTLTVETVNKTTFV<br>TKEEVMTHEAQGPLRPVVSAILINARQD
ZmGLP51	Zm00001eb28<br>2040_P001	MAKMVLLCVLVSFLLMPLASLALTQDFCVADLTCSDTPAGYPCKSSVTANDFYFHGLAGQGKINPLI<br>KAAVTPAFVGQFPGVNGLGISAARLDIEVGGVVPLHTHPAGSELLFVTQGTVAAGFISSGSNTVYTKT<br>LYAGDIMVFPQGLLHYQYNAGTGAAVGLVAFSSPNPGLQITDFALFANNLPSAVVEKVTFLDDAQVK<br>KLKSVLGGSG
ZmGLP52	Zm00001eb19<br>7900_P001	MHHLWMHSFPISLFNPCSAPLFPSQKFQEMNTLLSHLCCCILILCIYAPTSATSDNPPLQDVCPMAPQG<br>ERRELSMNGFLCKHPSTILASDFKTLLLNHAGDLDSIARSSVSMVTAAEFPGLNTLGLSMARTDIAPY<br>GVVLPHSHPRASEMMFVHGGSVVVGFLDTEGRLFQKRLGEGEVFVFPRGLLHYVMNYGFGLATAFS<br>VLNSQNPGVVGVAHAMFFASDSDVVEGLMARMLKFGEMEVTGDNNITAGFPWAF
ZmGLP53	Zm00001eb06<br>0460_P002	MKVPVLLLLVSLCFSLALAWQTDTESGSGRPYHYGEESFRHWTRSRQGRFRVLERFTHELLEDAVGN<br>YRVAELEAAPRAFLQPSHYDADEVMFVKEGEGVIVLLRGGKRESFCVREGDVMVIPAGAVVYSANT<br>HQSEWFRVVMLLSPVVSTSGRFEEFFPIGGESPESFLSVFSDDVIQASFNTRREEWEKVFEKQSKGEIT<br>TASEEQIRELSRSCSRGGRSSRSEGGDSGSSSSKWEIKPSSLTDKKPTHSNSHGRHYEITGDECPHLRLL

		DMDVGLANIARGSMMAPSYNTRANKIAIVLKGQGYFEMACPHVSGGRSSPRRERGHGREEEEEREEEQGGGGGGQKSRSYRQVKSRIREGSVIVIPAGHPTALVAGEAAGVRGGRGAAGGPCHRSTEGRRLPARAPEPQGLVGVSSACLRCSGRSSASRPRRSRPPRW
ZmGLP54	Zm00001eb140880_P001	MANKLATTSLLAAVLLALAAPSLAGDPDMLQDICVADYKSLQGPLRLNGFPCKRPENVTADDFFSNALASPGSTCNALGSAVTPADVRTLPGLNTLGVSVSRIDYAPWGVNPPHVHPRATEVIFVLQGSLDVGFVAATATGGSRRPPRNSGGSPAAVLSAFDSQLPGTQPVAEALFGASPPVPTDVLARSFQVDGGLVEAIRSNFPPK
ZmGLP55	Zm00001eb294140_P001	MAKAELLLALVASLVVLPFSSLAVYQDFCIADLSAAATPGGYPCKPPKDVTADDFHYGGLATPGTALKPFKISLGSAVVTTFPGLNGLGISAARMVMVPGGVAPLHSHPGGTELIFVIEGSVVCGFISATLNRVYTKTLYAGDLMVLPQGQLHFQYSHGNTTAVTLSSFSSDNPGLQILDFALFANDLPTEVVNKVTNLDELQIVKLKALFGGRG
ZmGLP56	Zm00001eb171490_P001	MAASTYLLLVAFLALLTSQAIASDPSPLQDFCVADKHSPVNVNGFVCKDPMTVNADDFFKAAKLDQPRDTTKSKVGSNVTLINVMQLPGLNTLGISLARIDYAPLGQNPPHTHPRATEILTVLEGTLYVGFVTSNQADNTNKLFSKVLNKGDVFVFP
ZmGLP57	Zm00001eb095950_P001	MGRLSCTYTTVLVALALSAPLAALAGDPDILTDYIVPADANPGTTNGTFFTFTGFRAAMTMPMPNFMPTKATMAEFPALNGQSVSYVLLMFPAGGSVNPTHTHPRSAELLLLLDGALSVGFVDTAGNLFTQDLAAGDMFVFPKGTVHWQYNQGTQPAKALSAFGSAAPGLVSLPSTLFGASNIDDNVLATSFKTDVATIQKLKAGLHA
ZmGLP58	Zm00001eb040290_P001	MHTHPEASELIFLLEGTLFAGFISAETNKAYVKILKKGDLYVFPQGLLHFQFNTGNTTATAIAAYSNQNPGLQIAVYALFGNTLTVETVNKTTFVTKEEVMRLKDLFGQSSVPS
ZmGLP59	Zm00001eb133240_P001	MVMVPGGVAPLHSHPGGTELIFVIEGSVVSGFISATLNRVYTKTLDNPGLQILDFALFANDLPTEVVNKVTNLDELQIVKLKALFGGRG
ZmGLP60	Zm00001eb084000_P001	LQIVCFEVHADRNEKVFLAGADNVLQKLDRVAKALSFAAKAEEVDEVLGSRHEKGFLPGPEESGGHEEREQEEEEREERHGGRGVRERHRREEREKEEEEREGRHGRGRREEVVETLMRMVTARM
