# Supplementary material for: Genome-Wide Identification and Salinity Response Analysis of the Germin-like Protein (GLP) Gene Family in Puccinellia tenuiflora
Source: Plants (Basel). 2025 Jul 22;14(15):2259. doi: 10.3390/plants14152259 (PMC12348945; doi:10.3390/plants14152259)
Supplement: Supplementary file 1 [file plants-14-02259-s001.zip › Table S3.pdf]

Table S3. List of *GLP* orthologous gene pairs identified between alkaligrass (*Puccinellia tenuiflora*) and three angiosperm species, rice (*Oryza sativa*), maize (*Zea mays*), and soybean (*Glycine max*).

PutGLPs		Orthologous gene pairs	
Chromosome	Locus	Chromosome	Locus
Puccinellia tenuiflora vs *Oryza sativa*			
Chr01	PutGLP45	Chr03	LOC_Os03g58980.1 (OsGLP3-7)
Chr04	PutGLP34	Chr01	LOC_Os01g72290.1 (OsGLP1-4)
Chr04	PutGLP8	Chr08	LOC_Os08g08990.1 (OsGLP8-5)
Chr04	PutGLP4	Chr08	LOC_Os08g09040.1 (OsGLP8-9)
Chr05	PutGLP38	Chr05	LOC_Os05g19670.1 (OsGLP5-2)
Chr06	PutGLP40	Chr01	LOC_Os01g18170.1 (OsGLP1-2)
Chr06	PutGLP36	Chr01	LOC_Os01g14670.1 (OsGLP1-1)
Chr07	PutGLP54	Chr09	LOC_Os09g39510.1 (OsGLP9-1)
Chr07	PutGLP53	Chr09	LOC_Os09g39510.1 (OsGLP9-1)
Puccinellia tenuiflora vs *Zea mays*			
Chr01	PutGLP45	Chr01	Zm00001eb061410_P001 (ZmGLP43)
Chr03	PutGLP22	Chr02	Zm00001eb070560_P001 (ZmGLP34)
Chr03	PutGLP23	Chr02	Zm00001eb070560_P001 (ZmGLP34)
Chr04	PutGLP9	Chr010	Zm00001eb413250_P001 (ZmGLP25)
Chr04	PutGLP34	Chr03	Zm00001eb140880_P001 (ZmGLP54)
Chr04	PutGLP11	Chr04	Zm00001eb171650_P001 (ZmGLP8)
Chr04	PutGLP12	Chr04	Zm00001eb171630_P001 (ZmGLP23)
Chr06	PutGLP40	Chr03	Zm00001eb129440_P001 (ZmGLP7)
Chr06	PutGLP35	Chr03	Zm00001eb140880_P001 (ZmGLP54)
Chr06	PutGLP40	Chr08	Zm00001eb332460_P001 (ZmGLP19)
Chr07	PutGLP54	Chr02	Zm00001eb095950_P001 (ZmGLP57)
Puccinellia tenuiflora vs *Glycine max*			
Chr06	PutGLP36	Chr19	EU916269 (GmGER1)
