# Supplementary material for: Genome-Wide Identification and Salinity Response Analysis of the Germin-like Protein (GLP) Gene Family in Puccinellia tenuiflora
Source: Plants (Basel). 2025 Jul 22;14(15):2259. doi: 10.3390/plants14152259 (PMC12348945; doi:10.3390/plants14152259)
Supplement: Supplementary file 1 [file plants-14-02259-s001.zip › Table S4.pdf]

Table S4. Primer sequences were designed for both RT-qPCR analysis of *GLP* gene expression and vector construction of *PutGLP37* in *Puccinellia tenuiflora*.

Gene name	Forward primer (5'-3')	Reverse primer (5'-3')
PutGLP1	TAGCTTCTGATCCTGGCCCT	GAAGTCCTCGGCTGTCACAA
PutGLP2	TAGCTTCTGATCCTGGCCCT	GAAGTCCTCGGCTGTCACAA
PutGLP4	GACATGCCGAGAGACACCAA	CAATTCGCGCCAAGGAGATG
PutGLP13	CAAGGTCGGGTCCAACGTTA	CAGCTTGTTTGGGTTGGACG
PutGLP15	TGTCACGTCCAACCCAAACA	ACCAGGGTTTTGGCTGTTGA
PutGLP32	GGTCATCTCGGCCTTCAACA	GTCCTCCGTCGATCTGGAAC
PutGLP33	CGCCACCGAGATCATCTTCA	TGTTGAAGGCCGAGATGACC
PutGLP34	GCTCAACACCCTAGGCATGT	TCCCTCGAGCACGTAGATGA
PutGLP36	CATCGCCATGACGCTCTTTG	TGGCCTTGATCTTGTCCACC
PutGLP37	ACTACCTCCAGGACCTCTGC	TTGGAGAAGAAGTCGTCCGC
PutGLP38	CCTCGACGTCGGCTTCATAA	TCGCCGTTGTTCTTCTGGAA
PutGLP42	GCTCGAGGTGGGTTTCATCA	CCCTGTTTTGCTGGAAGTGC
PutGLP44	ATGGTGCACTTCCAGCTCAA	CCCTGATCCCAGACCCAAAC
PutGLP48	CTACTCCTTGGTGCTGGTGG	AGGCCTTGGTGATCGTGAAG
PutGLP54	GCATGCCGATGAACATCACC	CCTGGTGGAAACCTGAGCAT
PutActin	GGTAACATTGTGCTCAGTGGT<br>GG	AACGACCTTAATCTTCATGCT<br>GC
pET32a-<br>*PutGLP37*	GCCATGGCTGATATCGGATCCA<br>TGGCTCGGTCTTACTCTTCCA	TGCGGCCGCAAGCTTGTCGAC<br>GCCCTTCTTGGGCGCGAACT
pYES2-<br>*PutGLP37*	GCCGCCAGTGTGCTGGAATTC<br>ATGGCTCGGTCTTACTCTTCCA	TACATGATGCGGCCCTCTAGA<br>GCCCTTCTTGGGCGCGAACT
pCAMBIA1300<br>*-PutGLP37-*<br>*GFP*	 TTACAATTACAATTAGGATCCA<br>TGGCTCGGTCTTACTCTTCCA	 CATAGGCCTACTAGTTCTAGA<br>GCCCTTCTTGGGCGCGAACT
