# Supplementary figures and images for: Genome-Wide Identification and Salinity Response Analysis of the Germin-like Protein (GLP) Gene Family in Puccinellia tenuiflora
Source: Plants (Basel). 2025 Jul 22;14(15):2259. doi: 10.3390/plants14152259 (PMC12348945; doi:10.3390/plants14152259)

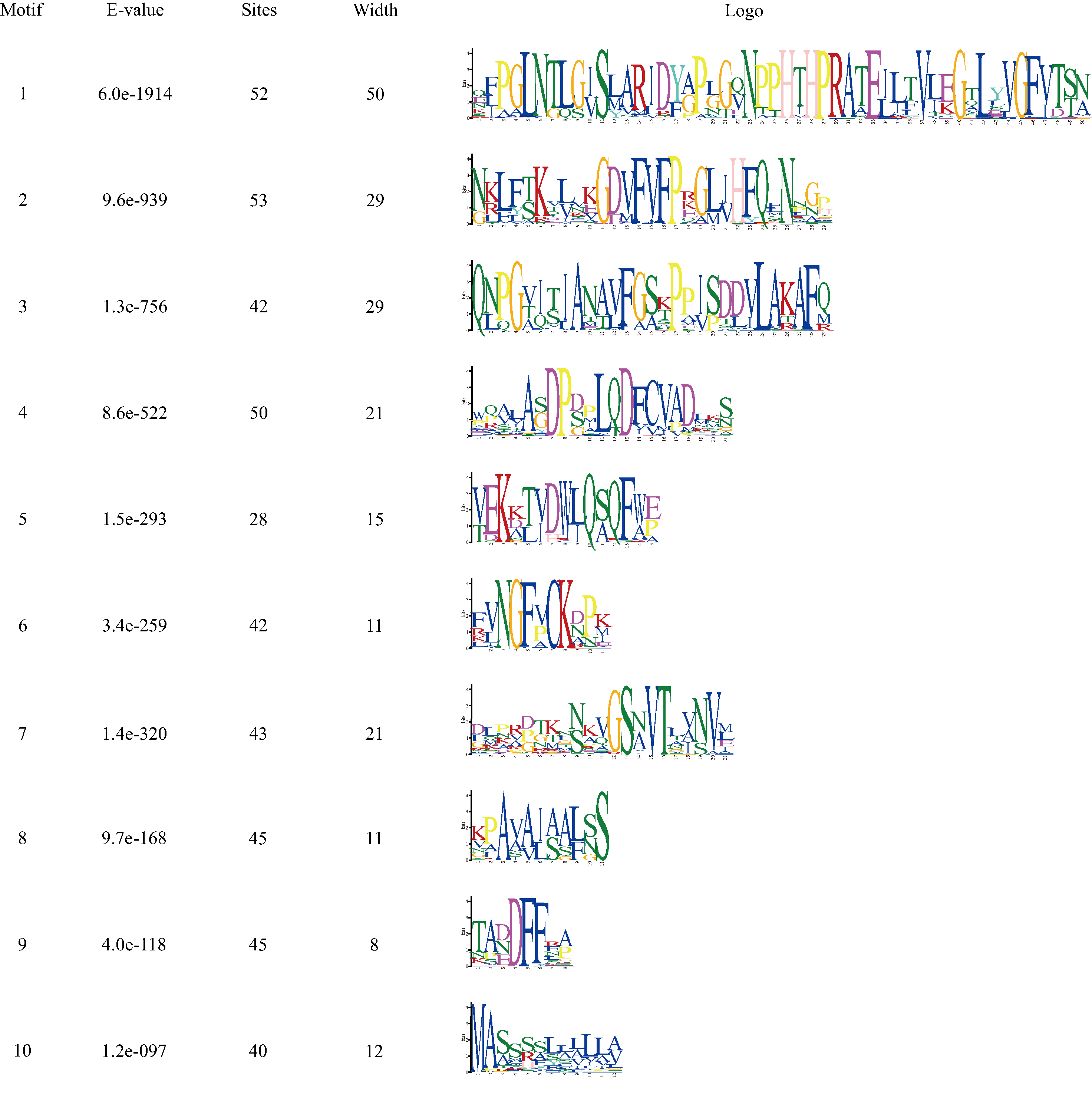

Supplement: Supplementary file 1 [file plants-14-02259-s001.zip › Figure S1.tif]

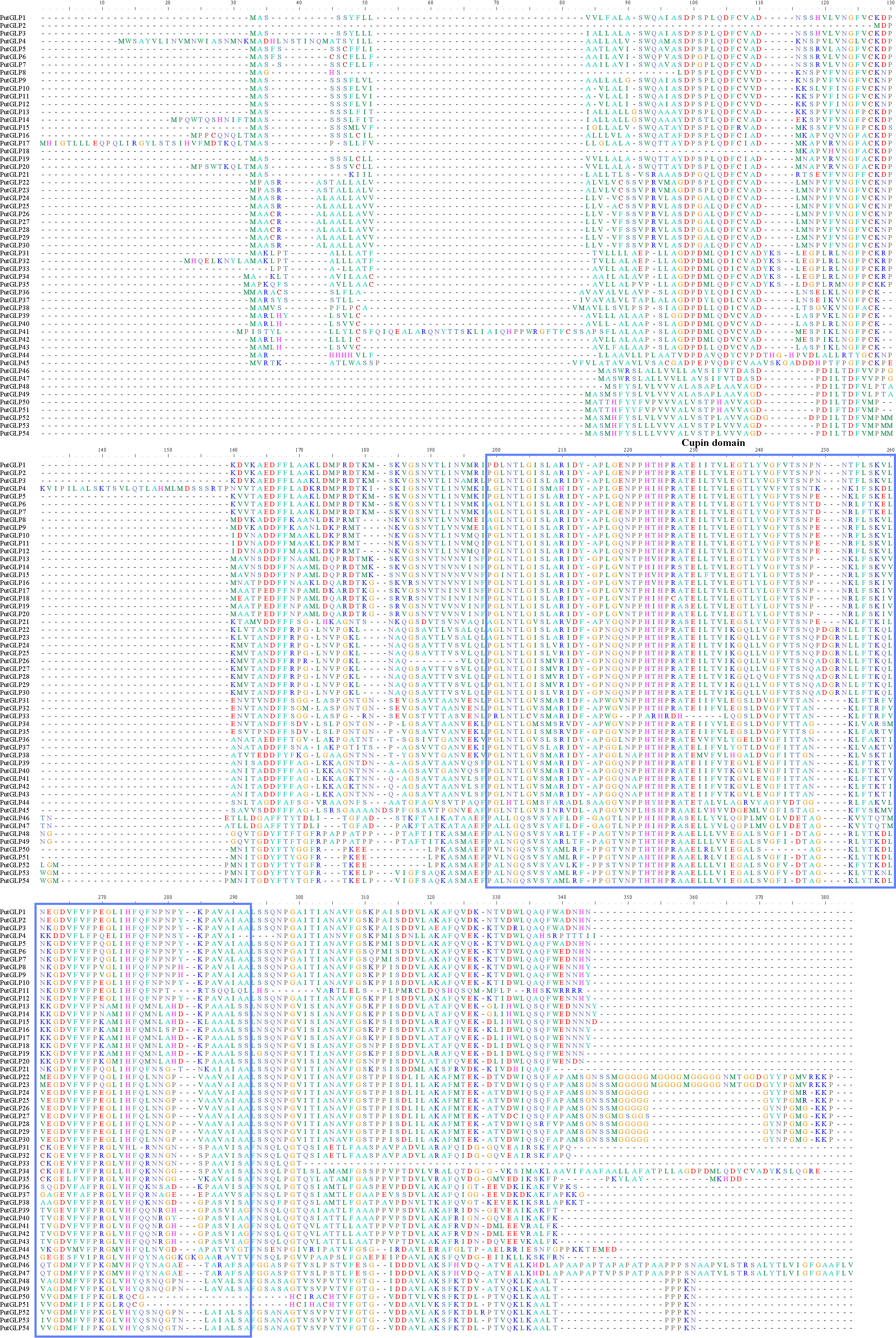

Supplement: Supplementary file 1 [file plants-14-02259-s001.zip › Figure S2.tif]

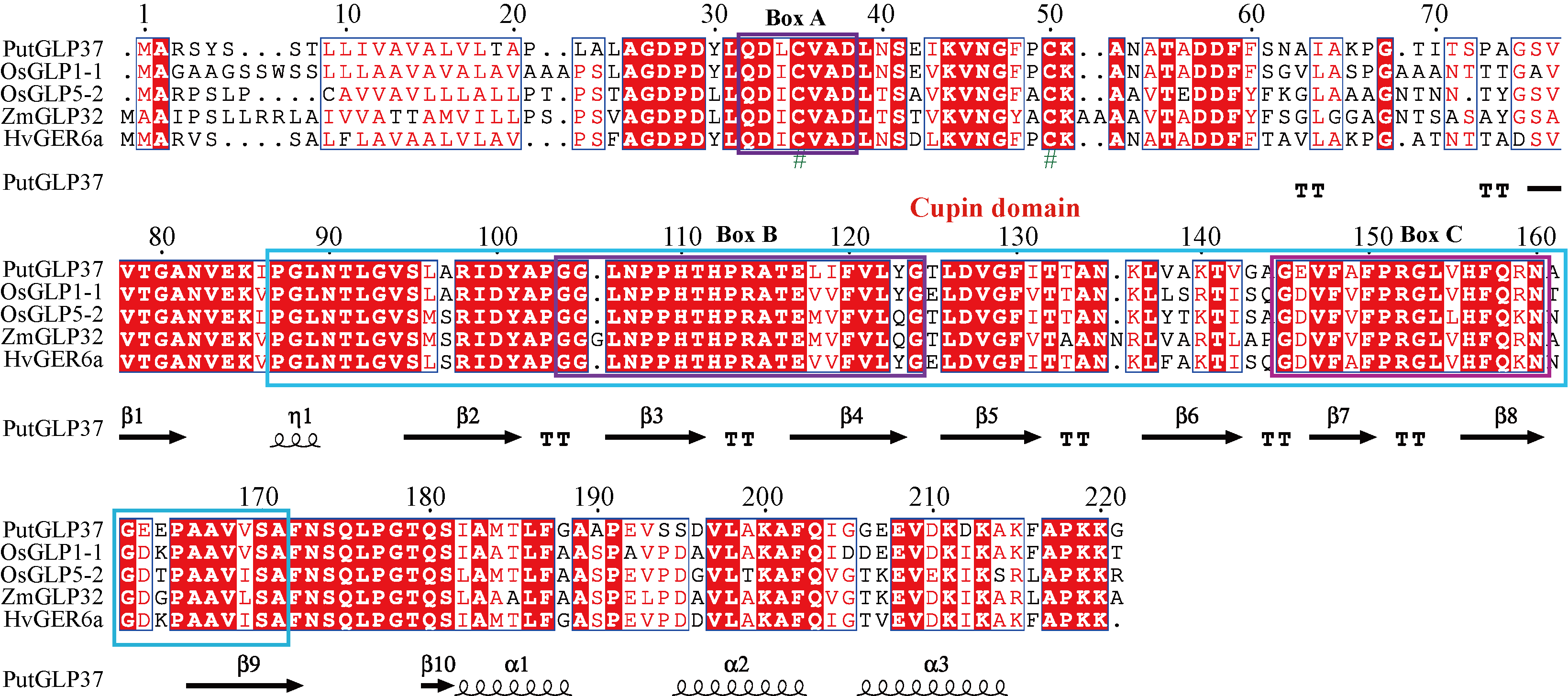

Supplement: Supplementary file 1 [file plants-14-02259-s001.zip › Figure S3.tif]

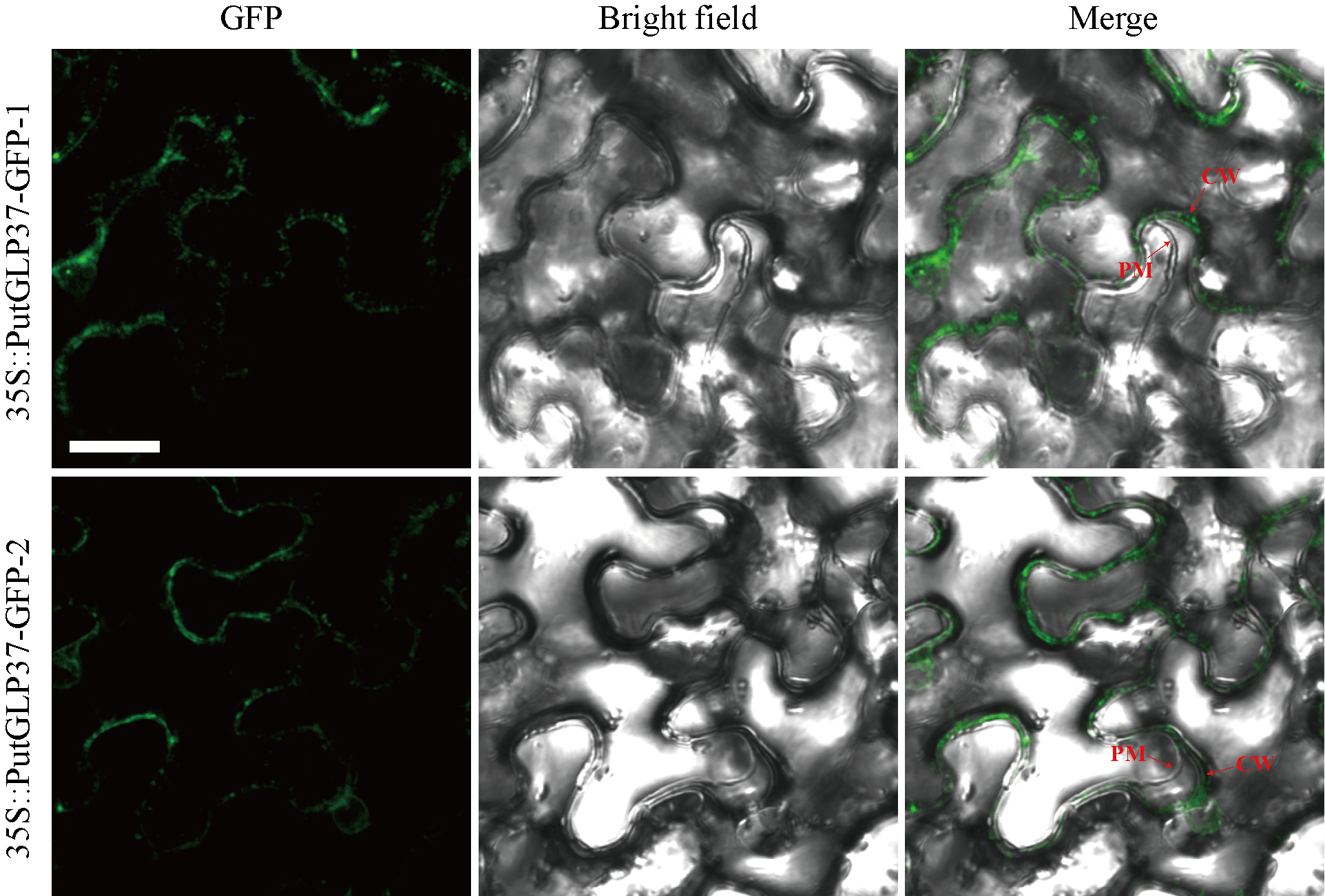

Supplement: Supplementary file 1 [file plants-14-02259-s001.zip › Figure S4.tif]

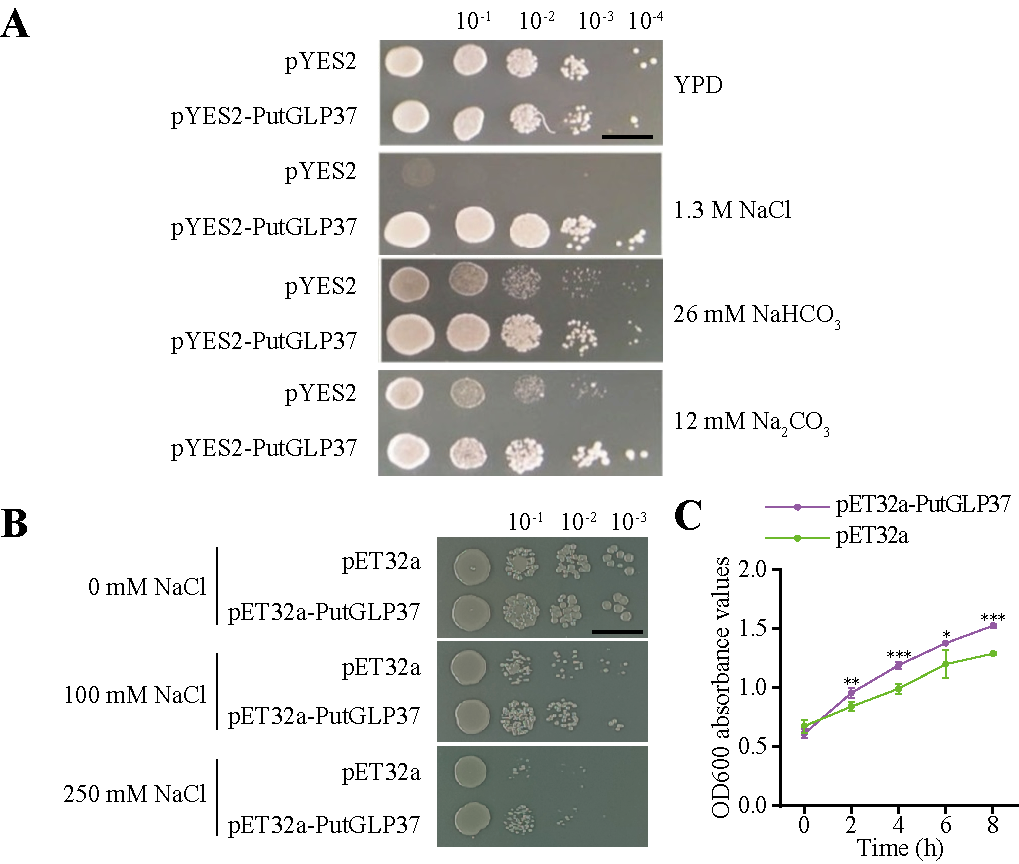

Supplement: Supplementary file 1 [file plants-14-02259-s001.zip › Figure S5.tif]
